# PU.1 target genes undergo Tet2-coupled demethylation and DNMT3b-mediated methylation in monocyte-to-osteoclast differentiation

**DOI:** 10.1186/gb-2013-14-9-r99

**Published:** 2013-09-12

**Authors:** Lorenzo de la Rica, Javier Rodríguez-Ubreva, Mireia García, Abul BMMK Islam, José M Urquiza, Henar Hernando, Jesper Christensen, Kristian Helin, Carmen Gómez-Vaquero, Esteban Ballestar

**Affiliations:** 1 Chromatin and Disease Group, Cancer Epigenetics and Biology Programme (PEBC), Bellvitge Biomedical Research Institute (IDIBELL), L’Hospitalet de Llobregat, Barcelona 08908, Spain; 2 Rheumatology Service, Bellvitge University Hospital (HUB), L’Hospitalet de Llobregat, Barcelona 08908, Spain; 3 Department of Experimental and Health Sciences, Barcelona Biomedical Research Park, Universitat Pompeu Fabra (UPF), Barcelona 08003, Spain; 4 Department of Genetic Engineering and Biotechnology, University of Dhaka, Dhaka 1000, Bangladesh; 5 Biotech Research and Innovation Center (BRIC), Center for Epigenetics University of Copenhagen, Ole Maaløes Vej 5, Copenhagen 2200, Denmark

## Abstract

**Background:**

DNA methylation is a key epigenetic mechanism for driving and stabilizing cell-fate decisions. Local deposition and removal of DNA methylation are tightly coupled with transcription factor binding, although the relationship varies with the specific differentiation process. Conversion of monocytes to osteoclasts is a unique terminal differentiation process within the hematopoietic system. This differentiation model is relevant to autoimmune disease and cancer, and there is abundant knowledge on the sets of transcription factors involved.

**Results:**

Here we focused on DNA methylation changes during osteoclastogenesis. Hypermethylation and hypomethylation changes took place in several thousand genes, including all relevant osteoclast differentiation and function categories. Hypomethylation occurred in association with changes in 5-hydroxymethylcytosine, a proposed intermediate toward demethylation. Transcription factor binding motif analysis revealed an over-representation of PU.1, NF-κB, and AP-1 (Jun/Fos) binding motifs in genes undergoing DNA methylation changes. Among these, only PU.1 motifs were significantly enriched in both hypermethylated and hypomethylated genes; ChIP-seq data analysis confirmed its association to both gene sets. Moreover, PU.1 interacts with both DNMT3b and TET2, suggesting its participation in driving hypermethylation and hydroxymethylation-mediated hypomethylation. Consistent with this, siRNA-mediated PU.1 knockdown in primary monocytes impaired the acquisition of DNA methylation and expression changes, and reduced the association of TET2 and DNMT3b at PU.1 targets during osteoclast differentiation.

**Conclusions:**

The work described here identifies key changes in DNA methylation during monocyte-to-osteoclast differentiation and reveals novel roles for PU.1 in this process.

## Background

DNA methylation plays a fundamental role in differentiation as it drives and stabilizes gene activity states during cell-fate decisions. Recent reports have shown a close relationship between the participation of transcription factors during differentiation and the generation of cell type-specific epigenetic signatures [1-3]. Several mechanisms explain the co-occurrence of DNA methylation changes and transcription factor binding, including the active recruitment of enzymes involved in DNA methylation deposition, interference, or alternative use of the same genomic regions. One of the best models for investigating these mechanisms is the hematopoietic differentiation system given the profound knowledge on the transcription factors implicated at different stages. Many studies have focused on hematopoiesis in order to learn about the type, distribution, and role of epigenetic changes, particularly DNA methylation during differentiation. However, the role of DNA methylation changes and the mechanisms participating in their acquisition in terminal differentiation processes remain elusive, even though these are among the most important since they produce functional cell types with very specific roles.

A singular differentiation process within the hematopoietic system is represented by differentiation from monocytes (MOs) to osteoclasts (OCs), which are giant, multinucleated cells that are specialized in degrading bone [4]. OCs differentiate from monocyte/macrophage progenitors following M-CSF [5] and RANKL [6] stimulation. Osteoclastogenesis requires cell fusion, cytoskeleton re-organization [7] and the activation of the specific gene sets necessary for bone catabolism. The signaling pathways activated after M-CSF and RANKL induction have been extensively described, and act through TRAF-6 [8,9], immunoreceptor tyrosine-based activation motif (ITAM) [10] adaptors DAP12 [11] and FcRγ [12] associated with their respective receptors, TREM-2 [13] and OSCAR, as well as calcium oscillations [14]. Signals end in the activation of NF-kB, MAPK, and c-Jun, leading to the activation of NFATc1 [15], the master transcription factor of osteoclastogenesis, together with PU.1 and MITF [16], which is already present in the progenitors. These transcription factors bind to the promoter and help upregulating OC markers such as dendritic cell-specific transmembrane protein (*DC-STAMP*/*TM7SF4*) [17], tartrate-resistant acid phosphatase (*TRACP/ACP5*) [18], cathepsin K (*CTSK*) [19], matrix metalloproteinase 9 (*MMP9*) [20], and carbonic anhydrase 2 (*CA2*).

OC deregulation is involved in several pathological contexts, either in the form of deficient function, as in the case in osteopetrosis [21], or aberrant hyperactivation, as in osteoporosis [22]. These cells are also involved in autoimmune rheumatic disease. For instance, in rheumatoid arthritis aberrantly activated OCs are major effectors of joint destruction [23]. Moreover, OCs cause bone complications in several diseases, such as multiple myeloma [24], prostate cancer, and breast cancer [25], and there is also a specific tumor with OC origin, the giant cell tumor of bone [26].

*In vitro* generation of OCs allows this cell type to be investigated, whereas isolating primary bone OCs for this purpose is very difficult. MOs stimulated with RANKL and M-CSF generate functional OCs [27], which degrade bone and express OC markers [28]. As indicated, the involvement of transcription factors in this model has been well studied, however very few reports have analyzed the role of epigenetic changes during osteoclastogenesis, and these focus mainly on histone modifications [29,30]. Given the relationship between transcription factors and DNA methylation, we hypothesized that examining DNA methylation changes would provide clues about the involvement of specific factors in the dynamics and hierarchy of these changes in terminal differentiation.

In this study, we compared the DNA methylation profiles of MOs and derived OCs following M-CSF and RANKL stimulation. We found that osteoclastogenesis was associated with the drastic reshaping of the DNA methylation landscape. Hypermethylation and hypomethylation occur in many relevant functional categories and key genes, including those whose functions are crucial to OC biology, like *CTSK*, *ACP5*, and *DC-STAMP*. Hypomethylation occurred early, concomitantly with transcription changes, was DNA replication-independent and associated with a change in 5-hydroxymethylcytosine, which has been proposed as an intermediate in the process of demethylation. Inspection of transcription factor binding motif over-representation in genes undergoing DNA methylation changes revealed the enrichment of the PU.1 binding motif in hypermethylated genes and AP-1, NF-kB, and also PU.1 motifs among hypomethylated genes. In fact, analysis of PU.1 ChIPseq data showed its general association to a high number of both hypo- and hypermethylated sites. Chromatin immunoprecipitation assays and immunoprecipitation experiments suggested a potential novel role for PU.1 recruiting DNMT3B to hypermethylated promoters, and TET2, which converts 5-methylcytosine to 5-hydroxymethylcytosine, to genes that become demethylated. This has been demonstrated by performing siRNA-mediated downregulation of PU.1 which partially impaired DNA methylation, expression, and recruitment of TET2 and DNMT3B to PU.1 targets, supporting the participation of PU.1 in the acquisition of DNA methylation changes at their target sites.

## Results

### Cell differentiation and fusion in osteoclastogenesis are accompanied by hypomethylation and hypermethylation of key functional pathways and genes

**Figure 1:**
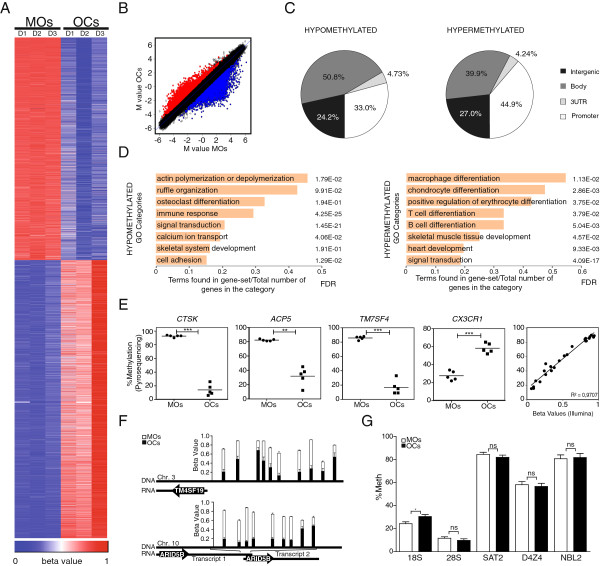
**High-throughput methylation comparison between monocytes (MOs) and derived osteoclasts (OCs). (A)** Heatmap including the data for three paired samples of MOs (MO D1, D2, D3) and their derived OCs (OC D1, D2, D3) harvested on day 21. The heatmap includes all CpG-containing probes displaying significant methylation changes (8,028 in total with FC ≥2 or FC ≤0.5; *P* ≤0.01 and FDR ≤0.05) (Additional file 2). Scale shown at the bottom, whereby beta values (that is, the ratio of the methylated probe intensity to the overall intensity, where overall intensity is the sum of methylated and unmethylated probe intensities) ranging from 0 (unmethylated, blue) to 1 (completely methylated, red). **(B)** Scatterplot showing the mean methylation profile of three matching MO/OC pairs. Genes with significant differences (FC >2, FDR <0.05) in averaged results from the three pairs of samples are highlighted in red (hypermethylated) or blue (hypomethylated). **(C)** Distribution of differentially methylated CpGs among genomic regions (promoter, gene bodies, 3′UTR, and intergenic) in different subsets of CpGs (hypomethylated, hypermethylated). **(D)** Gene ontology enrichment analysis of hypomethylated and hypermethylated CpGs showing the most important categories. **(E)** Technical validation of the array data by bisulfite pyrosequencing of modified DNA. BS pyrosequencing of three representative hypomethylated genes (*ACP5*, *CTSK*, and *TM7SF4*) and one hypermethylated gene (*CX3CR1*) from the array data are shown. A representation showing the excellent correlation between array data (beta values) and pyrosequencing data (% methylation) including the data for the four genes (right panel). **(F)** Cluster analysis of contiguous differentially methylated regions (<500 bp). Two examples of regions with more than nine consecutive CpGs differentially methylated are shown. **(G)** Analysis of methylation levels in repetitive elements (Sat2, D4Z4, NBL2) and ribosomal RNA genes (18S and 28S regions) as obtained from bisulfite sequencing analysis.

To investigate the acquisition of DNA methylation changes during monocyte-to-osteoclast differentiation we first obtained three sets of matching samples corresponding to MOs (CD14+ cells) from peripheral blood and OCs derived from the same CD14+ cells, 21 days after the addition of M-CSF and RANKL. The quality of mature, bone-resorbing OCs obtained under these conditions was confirmed by several methods, including the presence of more than three nuclei in TRAP-positive cells (in some cases, up to 40 nuclei per cell were counted), the upregulation of OC markers, such as *CA2*, *CTSK*, *ACP5*/*TRACP*, and *MMP9*, and downregulation of the monocytic gene *CX3CR1* (Additional file 1). At 21 days, over 84% of the nuclei detected in these preparations could be considered to be osteoclastic nuclei (in polykaryons, nuclei and not cells were counted) (Additional file 1). We then performed DNA methylation profiling using bead arrays that interrogate the DNA methylation status of >450,000 CpG sites across the entire genome covering 99% of RefSeq genes. Statistical analysis of the combined data from the three pairs of samples revealed that 3,515 genes (8,028 CpGs) displayed differential methylation (FC ≥2 or FC ≤0.5; false discovery rate (FDR) ≤0.05). Specifically, we identified 1,895 hypomethylated genes (3,597 CpG sites) and 2,054 hypermethylated genes (4,429 CpGs) (Figure 1A and Additional file 2). Changes corresponding to the average three pairs of monocytes/osteoclasts (Figure 1B) were almost identical to the pattern obtained for each individual pair of samples (Additional file 3), highlighting the specificity of the differences observed.

Over one-third of the differentially methylated CpG-containing probes (33% for hypomethylated CpGs, 45% for hypermethylated CpGs) mapped to gene promoters, the best-described regulatory region for DNA methylation, although DNA methylation changes also occurred at a similar scale in gene bodies (51% for hypomethylated CpGs, 40% for hypermethylated CpGs) (Figure 1C). Gene ontology analysis of hypomethylated CpGs revealed significant enrichment (FDR ≤0.05) for a variety of functional categories of relevance in OC differentiation and function (Figure 1D). We observed very high significance for terms like immune response (FDR = 4.25E-25) and signal transduction (FDR = 1.45E-21), but also more specific categories such as ruffle organization (FDR = 9.91E-2), calcium ion transport (FDR = 4.6E-2), and OC differentiation (FDR = 1.94E-1). In the case of hypermethylated genes, we also found highly significant enrichment of signal transduction (FDR = 4.09E-17), and enrichment of categories related to other hematopoietic cell types, suggesting that hypermethylation and associated silencing take place in gene sets that become silent in differentiated OCs (Figure 1D). Together, these data indicate that DNA hypomethylation is targeted to genomic regions that are activated during osteoclastogenesis, and hypermethylation silences alternative lineage genes that are not expressed in OCs.

Remarkably, among the group of hypomethylated genes (Additional file 2), we identified changes in several of the archetypal OC genes near their transcription start sites. For example, *CTSK*, the lysosomal cysteine proteinase involved in bone remodeling and resorption, is hypomethylated more than 60%. The *ACP5/TRACP* gene is hypomethylated around 47%. Finally, *TM7SF4*, which encodes for DC-STAMP, a seven-pass transmembrane protein involved in signal transduction in OCs and dendritic cells, undergoes 59% hypomethylation. We also observed significant hypomethylation at the osteoclast-specific transcription factor gene *NFATC1*, although in this case hypomethylation occurred at CpG sites located in its gene body region. Conversely, *CX3CR1*, an important factor for MO adhesion to blood vessels that is downregulated during osteoclastogenesis, displayed an increase in methylation of over 28% (Additional file 2).

To confirm that differences in DNA methylation identified between MOs and OCs were robust, we carried out bisulfite genomic pyrosequencing of the aforementioned selection of genes, looking at CpG sites corresponding to the oligonucleotide probe represented in the methylation array. In all cases, bisulfite pyrosequencing confirmed the results of the beadchip array (Figure 1E and Additional file 4). This analysis showed a very close correlation between the array and the pyrosequencing data (R^2^ = 0.9707) (Figure 1E).

We also investigated the coordinated hypomethylation or hypermethylation of adjacent CpGs by analyzing the different sequence window lengths (from 500 bp to 1,000,000 bp). With the largest sequence windows we were able to observe the coordinated hypermethylation of multiple CpGs across several genes, like those in the HOXA gene cluster. However, the majority of CpGs undergoing coordinated methylation changes were identified within the single gene level. By analyzing CpGs that are concomitantly deregulated within a 500-bp window, we identified several genes displaying coordinated hypomethylation or hypermethylation of many CpG sites (Additional file 5). Among these, we identified several CpG clusters in genes potentially involved in OC function and/or differentiation, including 10 CpGs at the promoter of the *TM4SF19* gene, also known as OC maturation-associated gene 4 protein, and nine CpGs in the gene body of *ARID5B*, the AT-rich interactive domain 5B (MRF1-like) (Figure 1F).

To examine the specificity of the DNA methylation changes further we performed bisulfite sequencing of repetitive elements (Sat2, D4Z4, and NBL2 repeats) and ribosomal RNA genes (Figure 1G and Additional file 3). We also performed genome-wide amplification of unmethylated DNA Alu repeats (AUMA), the most common family of repetitive elements that are present in tandem or interspersed in the genome [31]. These experiments showed no significant DNA methylation changes in any of these repetitive elements (Additional file 3), reinforcing the notion of the high specificity of hypomethylation and hypermethylation of the identified gene sets.

### Hypomethylation is replication-independent and involves changes in 5-hydroxymethylcytosine

To investigate the dynamics of DNA methylation in relation to gene expression changes we first examined how DNA methylation changes are associated with expression changes and then compared the dynamics of DNA methylation and expression changes.

**Figure 2:**
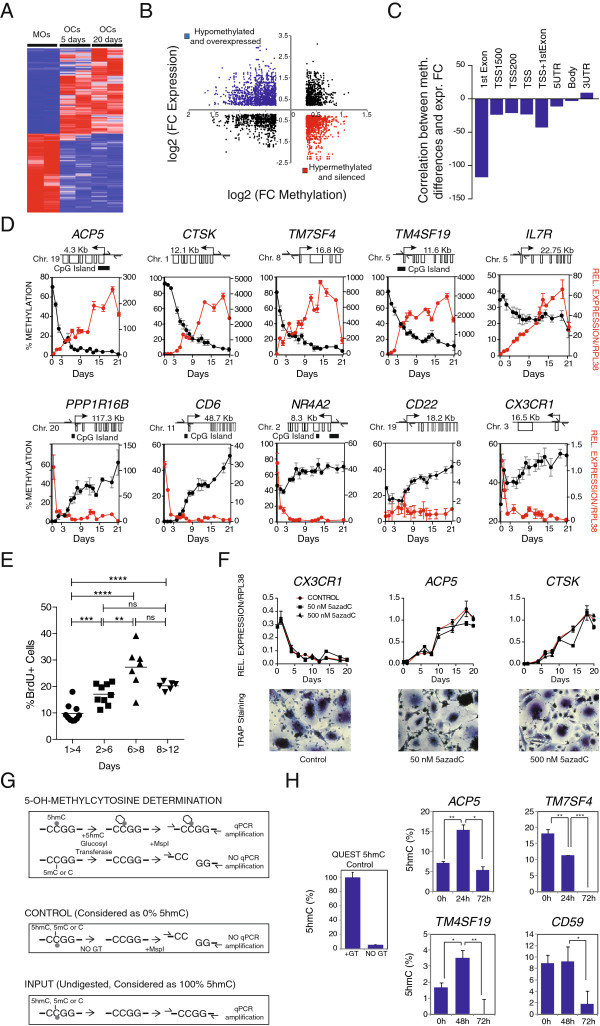
**Dynamics of DNA methylation and its relationship with expression changes. (A)** Heatmap showing expression levels on 0, 5, and 20 days for genes displaying significant methylation and expression changes (4,753 in total with FC ≥2 or FC ≤0.5; *P* ≤0.01 and FDR ≤0.05) (Additional file 7). **(B)** catterplots showing the relationship between the log_2_-transformed FC in expression and the log_2_-transformed FC in DNA methylation. Sixty-two percent of the hypomethylated genes are overexpressed (in blue); 55% of the hypermethylated genes are repressed (in red). **(C)** Correlation between methylation and expression data (slope from the linear regression between DNA methylation differences versus expression differences) for all differentially methylated genes organized by genomic location (first exon, TSS, 5′UTR, gene body, 3′UTR). **(D)** DNA methylation and expression dynamics of selected loci during monocyte-to-osteoclast differentiation. Methylation percentage determined by bisulfite pyrosequencing. Quantitative RT-PCR data relative to RPL38. DNA methylation and expression data are represented with a black line and a red line, respectively. **(E)** BrdU assay showing the percentage of replicating cells at different times. From days 1 to 4, only 9.46% of cells divide. **(F)** Effects of 5azadC treatment (50 nM, 500 nM) on osteoclastogenesis monitoring *ACP5*, *CTSK*, and *CX3CR1* levels and TRAP staining over time. **(G)** Workflow for testing the presence of 5 hydroxymethylcytosine in hypomethylated genes. DNA was treated with a 5hmC-specific glucosyltransferase. Cytosines bearing a 5-hydroxymethyl are protected against MspI digestion, and the surrounding region can be amplified by qPCR. When no 5hmC is present, glucose is not transferred to C, DNA is cleaved at CCGG sites, and there is less qPCR amplification. Several controls are used to set the 0% and 100% content of 5hmC. **(H)** 5hmC content in several of the CpGs that are rapidly demethylated after RANKL and M-CSF stimulation of OC precursors.

We used osteoclastogenesis expression data (available from the ArrayExpress database under accession number E-MEXP-2019) on 0, 5, and 20 days [32]. Our analysis showed that most changes occurred within the first 5 days, since the expression changes between 0 and 5 days were very similar to those observed between 0 and 20 days, and very few genes changed between 5 and 20 days (Figure 2A and Additional file 6). The 0-to-20-day comparison showed that 2,895 genes were upregulated (FC >2; FDR <0.05) and 1,858 were downregulated (FC <0.5; FDR <0.05). We found different relationships between DNA methylation changes and gene expression (Figure 2B). An inverse relationship between DNA methylation and gene expression was mainly observed for changes occurring in CpGs in the proximity of the TSS and within the first exon (Figure 2C) and it was less frequent in those at gene bodies and 3′UTR (Figure 2C). Comparing DNA methylation and expression data revealed that 452 genes were both hypomethylated and overexpressed and 280 genes were both hypermethylated and repressed at the selected thresholds (Additional file 7). We selected a panel of 10 genes from those undergoing hypomethylation and hypermethylation to investigate the dynamics of DNA methylation and expression changes, and performed bisulfite pyrosequencing and quantitative RT-PCR over the entire osteoclastogenesis for three sets of samples (Figure 2D). We found that the promoters of genes like *ACP5*, *CTSK*, *TM7SF4*, and *TM4SF19* rapidly became hypomethylated following RANKL and M-CSF stimulation (Figure 2D, top). In fact, around 60% of the entire range of hypomethylation occurred between days 0 and 4. Changes in mRNA levels occurred at a similar pace or, in some cases, in an even more gradual manner and were slightly delayed with respect to changes in DNA methylation. In contrast, hypermethylated genes like *PPP1R16B*, *CD6*, and *NR4A2* (Figure 2D, bottom) displayed loss of expression before experiencing an increase in DNA methylation, highlighting the different dynamics and mechanisms involved in hypomethylation and hypermethylation events.

It is well established that osteoclastogenesis occurs in the absence of cell division. We tested the levels of cell division in our monocyte-to-osteoclast differentiation experiments by treating cells with BrdU pulses. Consistent with previous observations, fewer than 9.8% were found to be BrdU-positive between 1 and 4 days, confirming the virtual absence of replication (Figure 2E and Additional file 8). This implies that the large DNA methylation changes observed in this time period are independent of DNA replication. This conclusion is also supported by the fact that treatment with 5-Aza-2′-deoxycytidine (5azadC), a pharmacological compound that results in replication-coupled DNA demethylation [33], had no significant effect on osteoclastogenesis (Figure 2F).

The existence of DNA methylation changes in the absence of replication is particularly significant for genes undergoing demethylation, given the controversy around active DNA demethylation mechanisms. In this context, recent studies have drawn attention towards a family of enzymes, the Tet proteins, which convert 5-methylcytosine (5mC) to 5-hydroxymethylcytosine (5hmC) [34,35] and other modified forms of cytosine, 5-formylcytosine (5fC) and 5-carboxylcytosine (5caC) [36]. 5hmC, 5fC, and 5caC may represent intermediates in an active demethylation pathway that ultimately replaces 5mC with cytosine in non-dividing cells [37,38]. To establish the potential involvement of these mechanisms, we here focused on the 5hmC levels at early time points in several of the genes that are hypomethylated during osteoclastogenesis, using a method that cleaves DNA that has C, 5mC, but not the glucosyl-5hmC produced as a result of treatment with the 5-hydroxymethylcytosine specific glucosyltransferase enzyme (Figure 2G). For several genes that become hypomethylated, like *ACP5* and *TM4SF19*, we observed an initial increase in 5hmC levels followed by a slight but significant decrease (Figure 2H). In other genes, like *TM7SF4* and *CD59*, of there were high levels of 5hmC before the addition of RANKL/M-CSF as if these genes were already primed for demethylation. In any case, our results suggested the participation of hydroxymethylation, and therefore the activity of Tet proteins, in genes that undergo a reduction in DNA methylation.

### Sequences undergoing DNA methylation changes are enriched for binding motifs for AP-1, NF-kB, and PU.1, key transcription factors in osteoclastogenesis

**Figure 3:**
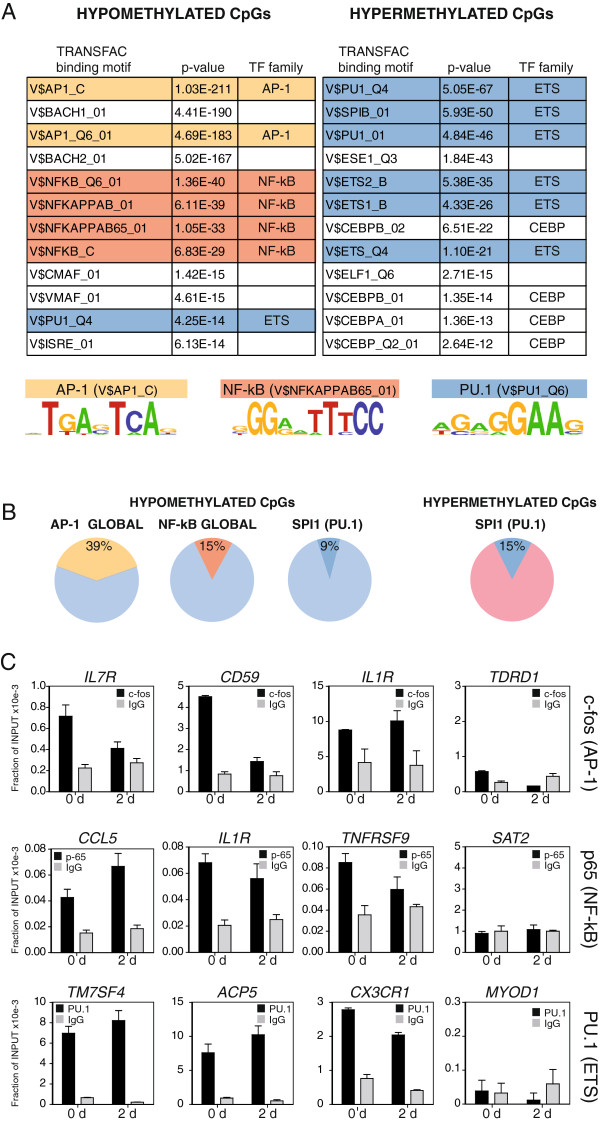
**Association of transcription factors with DNA methylation changes during moncoyte to OC differentiation. (A)** Significant enrichment of predicted TF (TRANSFAC motif) in hypo-/hypermethylated CpG sites regions. A 500-bp window centered around the hypo-/hypermethylated CpG sites was tested. The name of the transcription factor binding motif, the *P* value and the TF family are provided. Below we show three of the motifs that have a higher representation in this analysis. **(B)** Diagrams showing the percentage of hypo-/hypermethylated CpGs with AP-1, NF-kB, and PU.1 binding sites relative to the total number of hypo-/hypermethylated CpGs. **(C)** Quantitative ChIP assays showing the binding of three selected transcription factors (p65 NF-kB subunit, Fos, and PU.1 to target genes selected by the presence of the putative binding motifs according to the TRANSFAC analysis). Samples were analyzed at 0 and 2 days after RANKL/M-CSF stimulation. We used Sat2 repeats and the TRDR1 MyoD1 promoter as negative control sequences.

Different studies have recently shown that transcription factor binding events are associated with changes in the DNA methylation profiles and the response to different situations [2,39,40]. To address this further, we first investigated the potential over-representation of transcription factor binding motifs among the sequences undergoing DNA methylation changes during OC differentiation using the TRANSFAC database and focusing on a region of 500 bp around the CpG sites identified as undergoing hypomethylation or hypermethylation. We noted highly significant overrepresentation of a small selection of transcription factor binding motifs for genes that undergo hypomethylation or hypermethylation (Figure 3A). We observed that the over-representation of binding motifs was very specific to the direction of the DNA methylation change (hypomethylation or hypermethylation).

In the case of hypomethylated genes, we observed highly significant enrichment of binding motifs of the AP-1 family and NF-kB subunits (Figure 3A). We also observed enrichment of PU.1 (FDR 1.07E-12). In fact, 39% of all hypomethylated genes had binding motifs for AP-1, 15% genes had NF-kB binding motifs, and another 15% genes had binding motifs for PU.1 or other ETS-related factors (PU.1 alone, 9%) (Figure 3B). As aforementioned, these three groups of TFs play critical roles in osteoclastogenesis [41]. For instance, c-Fos, a component of the dimeric TF AP-1, regulate the switch between monocytes/macrophages and OC differentiation. Fra-1 is downstream to c-Fos, whereas PU.1 and NF-kB are upstream. NF-kB is critical in the expression of a variety of cytokines involved in OC differentiation. In the case of hypermethylated genes, we identified even greater enrichment of the binding motifs of ETS-related transcription factors, especially PU.1 (Figure 3A). In fact, the PU.1 binding motif is present in 15% of all hypermethylated genes (Figure 3B). Other motifs of ETS-related transcription factors from our list of hypermethylated genes included SPIB, ESE1, ETS1, ETS2, and others (Figure 3A). Much lower or insignificant levels of enrichment were obtained for AP-1 family members and NF-kB subunits among the hypermethylated genes. Previous studies have shown that genes that become methylated during hematopoietic differentiation are characterized by ETS transcription factors [2]. This appears to be particularly relevant in monocytic differentiation [1]. Interestingly, most of the reports about the role of PU.1 in osteoclastogenesis are associated with the activation of osteoclast-specific genes. However, in relation with methylation changes, PU.1 appears to be better correlated with those changes in the direction of repression. Overall, the analysis of transcription factor motifs showed that several of the factors associated with osteoclastogenesis had a significant over-representation of their binding motifs among the sets of hypo-and hypermethylated genes (Additional file 9).

To confirm the association of some of these factors with genes that become hypo- and hypermethylated we performed chromatin immunoprecipitation (ChIP) assays with a selection of transcription factors including PU.1, the NFkB subunit p65 and c-Fos, given their known role in osteoclastogenesis as well as the presence of binding motifs for them around hypo- and hypermethylated genes (in the case of c-Fos, it was chosen as a component of the dimeric TF AP-1). To select candidate genes we considered genes with motifs for these three factors among the list of hypo- and hypermethylated genes. For instance, genes that become hypomethylated and have binding sites for p65 include *CCL5*, *IL1R*, and *TNFR5SF*. In the case of transcription factor c-Fos, we looked at genes that become hypomethylated like *IL7R*, *CD59*, and *IL1R*. In the case of PU.1, we chose key genes with PU.1 binding near the differentially methylated CpG, including *ACP*5 and *TM4SF7* (hypomethylated) and *CX3CR1* (hypermethylated). ChIP assays demonstrated the interaction of these factors with most of the aforementioned promoters, even before the stimulation with M-CSF and RANKL (Figure 3C), as if these genes were primed by these factors in monocytes. No binding was observed in control sequences like Sat2 repeats and the *MYOD1* and *TDRD1* promoters. Interestingly, in the case of PU.1, with both hypo- and hypermethylated genes displaying binding at 0 days, genes becoming demethylated showed a slight increase in PU.1 binding, whereas hypermethylated genes showed a slight decrease in PU.1 association (Figure 3C).

### PU.1 recruits DNMT3b and TET2 to hypermethylated and hypomethylated genes

**Figure 4:**
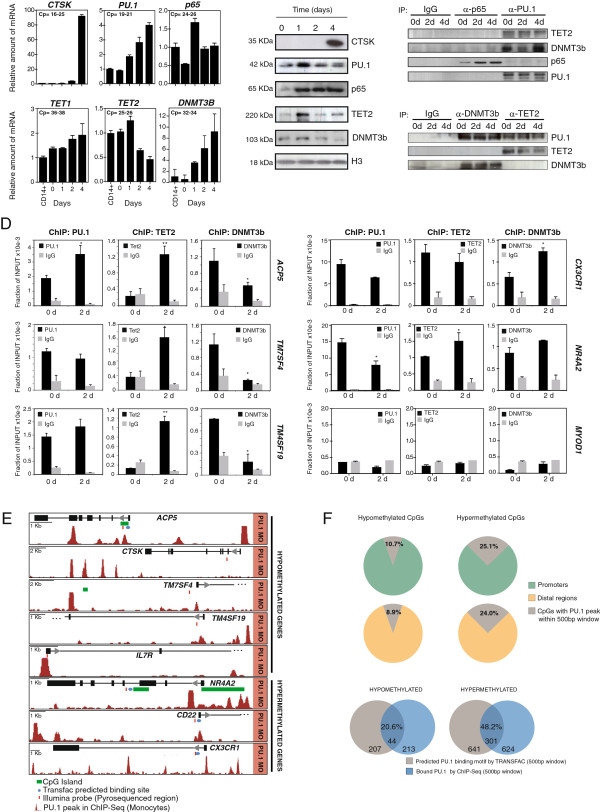
**Interactions between PU.1 and DNTM3b and TET2 and association with promoters undergoing DNA methylation changes. (A)** Quantitative RT-PCR analysis for *CTSK*, *PU.1*, *p65 NF-kB*, *TET1*, *TET2*, and *DNMT3B* during osteoclastogenesis. **(B)** Western blot for the same factors indicated above. **(C)** Immunoprecipitation experiment of p65 and PU.1 with DNMT3B and TET2 at 0, 2, and 4 days after RANKL and M-CSF stimulation. IgG used as a negative control. Reciprocal immunoprecipitation experiments in the bottom panel. **(D)** Quantitative ChIP assays showing PU.1, TET2, and DNMT3b binding to hypomethylated genes (*ACP5*, *TM7SF4*, *TM4SF19*) and hypermethylated genes (*CX3CR1*, *NR4A2*), all direct PU.1 targets, and a negative control *(MYOD1* promoter) without PU.1 target sites. The experiment was performed with three biological triplicates but only one experiment is shown. T-student test comparing binding of each antibody between 0 days *vs.* 2 days was performed: * corresponds to *P* value <0.05; ** means *P* value <0.01; *** means *P* value <0.001. **(E)** Examples showing PU.1 binding (from ChIPseq data, GSE31621) to the region neighboring hypo- and hypermethylated CpGs. The PU.1 binding motif location is presented as a horizontal blue dot and the CpG displaying differential methylation (Illumina probe) between MO and OC is marked with a red bar. **(F)** Analysis of ChIPseq analysis for PU.1 and comparison to TRANSFAC predictions. Top panel: proportion of the CpG-containing probes displaying DNA methylation changes that have peaks for PU.1 binding in the same 500-bp window. Diagrams are separated in the hypo- and hypermethylated sets and in promoter and distal regions (gene bodies, 3′UTR, and intergenic regions). Bottom panel, Venn diagrams showing the overlap of PU.1 targets from ChIPseq data (GEO accession number: GSE31621) in MOs and TRANSFAC prediction for PU.1, both using a window of 500 pb centered by the CpG displaying significant methylation changes.

To investigate the potential role of the aforementioned transcription factors in the acquisition of DNA methylation changes we chose PU.1 and NF-kB (p65 subunit) as two representative examples. We first checked their expression levels during osteoclastogenesis, by carrying out qRT-PCR and western blot assays. mRNA and protein analysis (Figure 4A and 4B) both confirmed the expression of these factors. PU.1 revealed an increase at the mRNA levels, although there was no change at the protein level. In the case of p65 NF-kB we only observed a clear increase at the protein level (Figure 4B). In parallel, we also confirmed the presence of DNMT3b, a *de novo* DNA methyltransferase, and the ten eleven translocation (TET) protein TET2, as enzymatic activities potentially related with DNA demethylation (Figure 4A and 4B). TET proteins are responsible for conversion of 5mC in 5hmC [34], 5fC, and 5cac [36]. Recent evidences support a role for TET-dependent active DNA demethylation process [42,43]. We focused on TET2 given their high levels in hematopoietic cells of myeloid origin [44,45]. Also, we have recently reported that TET2 plays a role in derepressing genes in pre-B cell to macrophage differentiation [44], and recent data shows that TET2 is required for active DNA demethylation in primary human MOs [45]. In fact TET1 and TET3 were undetectable in western blot (not shown) and qRT-PCR evidenced their low levels in this cell type (Figure 4A, only shown for TET1).

The confirmed binding of factors like PU.1 and the p65 subunit of NF-kB to hypo- and hypermethylated genes (Figure 3C) raised the possibility of their potential direct interaction with factors involved in maintaining the DNA methylation homeostasis. Some of these interactions have already been explored. For instance, PU.1 physically interacts with the *de novo* DNA methyltransferases DNMT3A and DNMT3B [46]. Such an interaction, if it occurred in osteoclastogenesis, could provide a potential mechanism to explain how PU.1 target genes become hypermethylated. One would expect that these transcription factors could also interact with factors participating in demethylation processes. Our previous results suggested the existence of 5hmC enrichment in genes that become hypomethylated, and therefore it is reasonable to test whether NF-kB p65 and PU.1 interact with Tet proteins, the enzymes catalyzing hydroxylation of 5mC.

We therefore tested the recruitment by NF-kB p65 and PU.1 of both DNMT3b and TET2 by carrying out immunoprecipitation assays with osteoclastogenesis samples 0, 2, and 4 days after stimulation with M-CSF and RANKL. Our results showed that PU.1 directly interacted with both DNMT3b and TET2 (Figure 4C). It is plausible that these two interactions may involve different subpopulations of PU.1, for instance with specific post-translational modifications like Ser phosphorylation. However, we did not address this aspect at this point. In the case of NF-kB, we did not observe binding with either of these factors (Figure 4C). This could perhaps be explained by the fact that p65 is shuttling back to the cytoplasm much of the time.

To confirm the interaction between PU.1 and DNMT3b and TET2, we performed reciprocal immunoprepitation experiments with anti-DNMT3b and anti-TET2. These confirmed the direct interaction with PU.1 (Figure 4C). Our results suggested that PU.1 may play a dual coupling transcription factor that can interact with the DNA methyltransferases and enzymes perhaps participating in, or leading to, demethylation. It is likely that other factors participate in the recruitment of these enzymes, however at this stage we focused on PU.1 because of its ability to bind both DNMT3b and Tet2 and its association with both hyper- and hypomethylated sequences.

We then investigated the dual role of PU.1 in recruiting TET2 and DNMT3b to promoters. To this end we performed chromatin immunoprecipitation assays with PU.1, TET2, and DNMT3b in MOs at 0 and 2 days following stimulation with M-CSF/RANKL. We amplified gene promoters with predicted binding sites for PU.1 that become both demethylated (*ACP5*, *TMS7SF4*, and *TM4SF19*) as well as hypermethylated (*CX3CR1* and *NR4A2*) and used a non-target of PU.1 (*MYOD1*) as negative control. Our analysis showed binding of PU.1 at both 0 and 2 days (Figure 4D). For genes that become hypomethylated, we observed an increased recruitment of TET2 at these promoters after 2 days, whereas DNMT3b was initially enriched but its association with these promoters was lost after M-CSF and RANKL stimulation (Figure 4D). In genes that become hypermethylated (*CX3CR1* and *NR4A2*), we also observed association of PU.1 at both 0 and 2 days. However we again observed a slight decrease at 2 days together. We also observed increased recruitment of DNMT3b at 2 days after M-CSF/ RANKL stimulation. We did not observe association of PU.1, DNMT3b, and TET2 in the negative control for PU.1 binding, the MyoD promoter.

To evaluate the extent to which hypo- and hypermethylated genes correlate with PU.1 occupancy, we used our DNA methylation data and PU.1 ChIPseq data (GSE31621) obtained in MOs [1]. Most of the individual example genes previously analyzed displayed PU.1 binding overlapping or in the proximity of the CpG sites undergoing a methylation change (Figure 4E). To systematize the analysis we used a window of 500 bp centered around the CpG displaying DNA methylation changes. Under these conditions we found that 10.7% of all hypomethylated CpGs located in promoter regions genes and 25.1% of all hypermethylated CpGs located in promoter regions had PU.1 peaks within this 500-bp window (Figure 4F). These numbers were similar when focusing on CpGs located in distal regions (Figure 4F). We also compared the ChIPseq data to the prediction by TRANSFAC analysis and observed that the overlap between the two sets of list was around 20.6% for hypomethylated genes and 46.9% for hypermethylated genes, again using the same 500 bp for both datasets (Figure 4F). These analyses reinforced the notion of PU.1 associated with a high number of genes undergoing DNA methylation changes; however, it also reveals the weakness in the predictive power of TRANSFAC motif searches and the need of experimental validation of its results.

### Dowregulation of PU.1 in MOs impairs activation of OC markers, hypomethylation, and recruitment of DNMT3b and TET2

**Figure 5:**
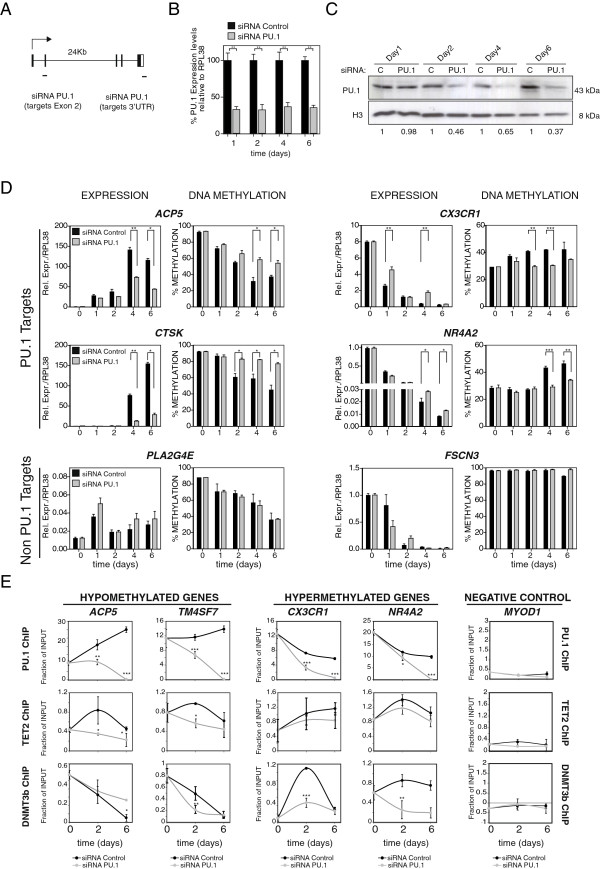
**PU.1 has a direct role in leading DNA methylation changes at their targets. (A)** Scheme depicting the two regions of the SPI1 gene (PU.1) (exon 2 and 3′UTR) targeted by the two siRNAs used in this study. **(B)** Effects of siRNA experiments on PU.1 levels at 1, 2, 4, and 6 days as analyzed by qRT-pCR. **(C)** Effects of siRNA experiments on PU.1 levels at 1, 2, 4, and 6 days as analyzed by western blot **(D)** Effects of PU.1 downregulation on expression and methylation of PU.1-target genes that become demethylated (*ACP5*, *CTSK*), genes that become hypermethylated (*CX3CR1*, *NR4A2*) and non-pU.1 target genes, *PLA2G4E*, which becomes also overexpressed and demethylated, and *FSCN3*, which is hypermethylated and does not undergo loss of methylation during osteoclastogenesis. **(E)** ChIP assays showing the effects of PU.1 downregulation in its recruitment, together with TET2 and DNMT3b binding to the same genes. Data were obtained at 0, 2, and 6 days after M-CSF /RANL stimulation. To simplify the representation negative control assays with IgG for each time point have been substracted to the experiments with each antibody. We have used the *MYOD1* promoter as a negative control (data without substracting the background is presented in Additional file 10). The experiment was performed with three biological triplicates but only one experiment is shown. Error bars correspond to technical replicates. Some of them are smaller than the data point icon. T-student test was performed: * corresponds to *P* value <0.05; ** means *P* value <0.01; *** means *P* value <0.001.

To investigate a potential causal relationship between PU.1 and DNA methylation changes in monocyte-to-osteoclast differentiation we investigated the effects of ablating PU.1 expression in MOs. We therefore downregulated PU.1 levels in MOs using transient transfection experiments with a mix of two siRNAs targeting exon2 and the 3′UTR of PU.1 (Figure 5A). In parallel, we used a control siRNA. Following transfection we stimulated differentiation using RANKL/M-CSF. In these conditions, we checked by qRT-PCR and western blot the effects on PU.1 levels at 1, 2, 4, and 6 days following RANKL/M-CSF stimulation of MOs and confirmed the PU.1 downregulation close to 60% (Figure 5B and 5C). We then observed that the upregulation of genes like *ACP5* and *CTSK* (both PU.1-direct targets) was partially impaired (Figure 5D). In the case of genes like *CX3CR1* and *NR4A2* we determined that downregulation was also impaired in PU.1-siRNA treated MOs. Interestingly, we also analyzed two genes that are not direct PU.1 targets, one upregulated and hypomethylated during osteoclastogenesis (*PLA2G4E*) and a second one, highly methylated, that does not experience DNA methylation changes during OC differentiation (*FSCN3*). PU.1-siRNA treatment had only small effects on gene expression changes during osteoclast differentiation (perhaps due to indirect effects) when compared to control siRNA, confirming the specificity of the changes observed for the other genes (Figure 5D, bottom).

We then tested the effects of PU.1 downregulation in DNA methylation changes. We looked at both PU.1-target genes that become hypomethylated and hypermethylated. In both cases, we observed that downregulation of PU.1 impaired the acquisition of DNA methylation changes, in contrast with the changes observed for control siRNA-treated MOs (Figure 5D). In the case of *TM7SF4*, one of the key genes undergoing hypomethylation, we did not detect an effect of PU.1 downregulation on its DNA methylation dynamics (Additional file 10). However, this could perhaps be explained because this gene undergoes changes before downregulation of PU.1 by siRNA is effective, within day 1 (Additional file 10) and suggests the participation of other factors in this process. At any rate, the observed effects only occurred in PU.1 targets. It did not affect genes that are not targeted by PU.1 (*PLA2G4E*, *FSCN3*). In the case of *PLA2G4E,* PU.1-siRNA treatment did not impair the loss of methylation that occurred in the control experiment. For *FSCN3*, we observed no loss of methylation in any case (Figure 5D).

Finally, we compared the effect of PU.1 downregulation in the recruitment of DNMT3b and TET2 to hyper- and hypomethylated promoters (Figure 5E and Additional file 10). As expected, we observed that PU.1 dowregulation resulted in a decrease of the levels of PU.1 associated with the promoters of both hypo- and hypermethylated genes. Most importantly, it also reduced the association of DNMT3b and TET2 reinforcing the notion of the role of these factors and their association with PU.1 in the DNA methylation changes occurring at these CpG sites (Figure 5E). The time course analysis (at 2 and 6 days) of these results also revealed a complex dynamics for the PU.1, TET2, and DNMT3b interactions with their target genes, particularly in the case of hypermethylated genes. It is possible that perturbation of PU.1 levels could be compensated by additional factors that participate in the acquisition of DNA methylation changes of these genes. These aspects will need to be further investigated.

## Discussion

Our results provide evidence of the participation of transcription factors, focusing on PU.1, in determining changes in DNA methylation during monocyte-to-osteoclast differentiation. First, a detailed analysis of the sequences undergoing DNA methylation changes produced evidences of the participation of several transcription factors, given the specific over-representation of certain motifs in hypo- and hypermethylated genes. This initial analysis was validated in several candidate genes and using ChIPseq data for human primary monocytes [1]. Second, further analyses on one these candidate transcription factors, PU.1, and manipulation of its levels revealed a novel role for this factor in mediating DNA methylation changes during osteoclastogenesis, by direct binding of both DNMT3B and TET2.

In general, DNA methylation changes in differentiation or any other dynamic process are of interest for two reasons: (1) these changes are generally associated with gene expression changes, particularly when associated with promoters or gene bodies, and reveal aspects intrinsic to identity and function of the corresponding cell types; (2) they can be considered as epigenetic footprints that, despite not necessarily being associated with an expression or organizational change, reveal a change in the milieu of a particular CpG and therefore can be used to trace the participation of specific transcription factors or other nuclear elements in that environment/neighborhood. This information can then be used to reconstruct cell signaling events, transcription factors involved and mechanisms participating in differentiation. In this sense, our data show that DNA methylation changes are involved in the differentiation dynamics and stabilization of the OC phenotype since they are concomitant with, or even precede, expression changes. These data are closely correlated with gene expression changes, and a majority of genes that undergo hypomethylation or hypermethylation at their promoters or gene bodies also experience a change in expression, although the relationship varies between different gene sets. Finally, gene ontology analysis reveals that all relevant functional categories and the majority of key genes for differentiation or the activity of functional OCs undergo DNA methylation changes and that genes within all relevant functional categories undergo DNA methylation changes.

Our study suggests that both hypomethylation and hypermethylation events are equally important. Hypomethylation events, in many cases associated with gene activation, affect genes that are specific to this differentiation process or are related with the function of differentiated OCs. In contrast, the identity of genes affected by hypermethylation events is less closely correlated with OC function, given that most of them are related with gene repression. In fact, we found that hypermethylation affects genes that are specific to other cellular types. Given that osteoclastogenesis involves cell fusion and the generation of highly polyploid cells, we had speculated whether the existence of redundant copies of genetic material could lead to massive gene repression, and the silencing of extra copies. However, hypermethylation does not seem to be predominant over hypomethylation. The two activities are very specific to particular gene sets and there are no indications of changes in repetitive elements.

A number of transcription factors are essential for OC formation. Some of these factors are involved in various differentiation processes. Among these, PU.1, c-Fos, NF-kB, and other factors are essential for osteoclastogenesis. In fact, NF-kB- and PU.1-deficient mice show a macrophage differentiation failure, and osteoclastogenesis is inhibited at an early stage of differentiation. c-Fos is a component of the dimeric TF AP-1, which also includes FosB, Fra-1, Fra-2, and Jun proteins such as c-Jun, JunB, and JunD. Other key factors involved in OC differentiation include C/EBPα [47] and Bach1 [48]. Osteoclastogenesis also depends on the activity of more specific transcription factors like NFATc1 and MITF. Interestingly, the analysis of the presence of transcription factor binding sites in sequences that undergo DNA methylation changes shows a significant enrichment in binding motifs of transcription factors that are key in OC differentiation, some of which we have validated for a selection of putative target genes.

One of the most interesting factors in this process is the ETS factor PU.1. In fact, PU.1 is the earliest molecule known to influence the differentiation and commitment of precursor myeloid cells to the OC lineage. PU.1 functions in concert with other transcription factors, including c-Myb, C/EBPα, cJun, and others, to activate osteoclast-specific genes.

Our results reveal two hitherto undescribed roles for PU.1 in the context of monocyte-to-OC differentiation. First, we have identified the association of PU.1 with genes that become repressed through hypermethylation and describe its direct interaction with DNMT3b in the context of osteoclastogenesis. Second, we identify a novel interaction between PU.1 and TET2 and their association with genes that become demethylated. Our study shows that PU.1 may act as a dual adaptor during osteoclastogenesis, in the directions of hypomethylation and hypermethylation. This is compatible with previous data on genome wide DNA methylation profiling comparing cell types across the hematopietic differentiation system where an over-representation of ETS transcription factor binding sites was found [2]. In monocyte-to-osteoclast differentiation, PU.1 is best known for its role in the activation of osteoclast-specific genes. However, studies in other models have previously shown that PU.1 can participate in the repression of genes in concert with elements of the epigenetic machinery. For instance, PU.1 is known to generate a repressive chromatin structure characterized by H3K9me3 in myeloid and erythroid differentiation [49]. Also, PU.1 has been shown to act in concert with MITF to recruit co-repressors to osteoclast-specific in committed myeloid precursors capable of forming either macrophages or OC [50]. Moreover, previous studies have shown that PU.1 can form a complex with DNMT3a and DNMT3b [46]. However, this is the first report where the association between PU.1 and DNMTs in association with gene repression is shown in this context.

Moreover, our findings constitute the first report where the binding of PU.1 to TET2 has been described. Several recent reports have pointed at TET2-mediated hydroxylation of 5-methylcytosine as an intermediate step towards demethylation [51] and our data show changes in 5hmC at genes that become demethylated in osteoclastogenesis, reinforcing the possibility that PU.1-mediated recruitment of TET2 is leading to 5hmC-mediated demethylation. However the detailed mechanisms that couple hydroxylation of 5mC and demethylation are still objects of debate.

**Figure 6:**
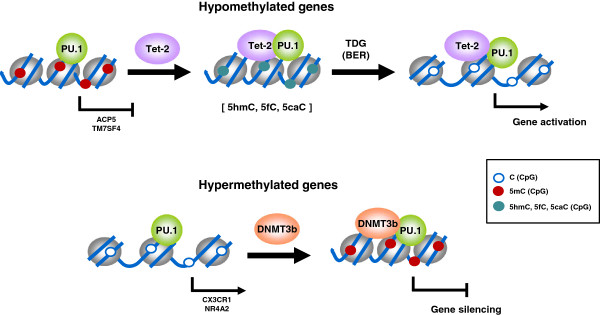
**Model showing a simplified diagram proposing the recruitment of TET2 and DNMT3b by PU.1 to its target genes that become hypo- or hypermethylated, respectively, during osteoclastogenesis.** Genes that become hypomethylated exchange PU.1-DNMT3b by PU.1-TET2 (although whether pre-existing subpopulations of these associations may exist or, alternatively, post-translational or another mechanisms may mediate exchange of TET2 and DNMT3b; this is not elucidated at present). TDG is likely to mediate conversion of 5hmC/5fmC/5caC to demethylated cytosine. Hypermethylated genes experience an increase in the binding of DNMT3b as differentiation to OCs is triggered.

The manipulation of PU.1 levels by using siRNAs has shown that PU.1 has a direct role in recruiting DNMT3b and TET2 to its target promoters, as well as showing how impaired association of PU.1 results in defective acquisition of DNA methylation changes in both directions as well as reduced effect on gene expression changes. Therefore, our data reveal a novel role of PU.1 as a dual adaptor with the ability to bind both epigenetically repressive and epigenetically activating events and targeting DNA methylation changes in both directions (Figure 6). The incomplete impairment of DNA methylation and expression changes, as well as partial loss of Tet2 and DNMT3b following PU.1 knock-down indicates that additional transcription factors are also participating in this process. In future studies, it will also be interesting to identify the mechanisms that operate in the specific recruitment of PU.1-TET2 to genes that become demethylated, and to determine how PU.1-DNMT3b is recruited to genes that become hypermethylated. It is likely that specific transcription factors play a role, and specific post-translational modifications in PU.1 may participate in the coupling of its associated complexes to specific factors. In this context, Ser phosphorylation of PU.1 has already been shown to play a role in its recruitment to promoters [52] and could also participate in discriminating interaction with epigenetic modifiers.

Our study has allowed us to identify key DNA methylation changes during OC differentiation and has revealed an implication of PU.1 in the acquisition of DNA methylation and expression changes as well as identifying novel interactions with DNMT3b and TET2.

## Conclusions

Our study of the DNA methylation changes in monocyte-to-osteoclast differentiation reveals the occurrence of both hypomethylation and hypermethylation changes. These changes occur in the virtual absence of DNA replication suggesting the participation of active mechanisms, particularly relevant for hypomethylation events, for which the mechanisms are still subject of debate. Also, when comparing the dynamics of DNA methylation and expression changes, hypomethylation occurs concomitant or even earlier than expression changes. In contrast, for the majority of genes becoming hypermethylated, hypermethylation follows expression changes. Hypomethylation takes place in relevant functional categories related with OC differentiation and most of the genes that are necessary for OC function undergo hypomethylation including *ACP5*, *CTSK*, and *TM7SF4* among others. The analysis of over-representation of transcription factor binding motifs reveals the enrichment of specific motifs for hypomethylated and hypermethylated genes. Among these, PU.1 and other ETS-related binding motifs are highly enriched in both hypomethylated and hypermethylated genes. We have demonstrated that PU.1 is bound to both hypo- and hypermethylated promoters and that it is able to recruit both DNMT3b and TET2. Most importantly, downregulation of PU.1 with siRNAs not only shows a reduction in the recruitment of these two enzymes to PU.1 target genes but also results in a specific reduction in the acquisition of DNA methylation and expression changes at those targets. Our results demonstrate a key role of PU.1 in driving DNA methylation changes during OC differentiation.

## Materials and methods

### Differentiation of OCs from peripheral blood mononuclear cells

Human samples (blood) used in this study came from anonymous blood donors and were obtained from the Catalan Blood and Tissue Bank (Banc de Sang i Teixits) in Barcelona as thrombocyte concentrates (buffy coats). The anonymous blood donors received oral and written information about the possibility that their blood would be used for research purposes, and any questions that arose were then answered. Prior to obtaining the first blood sample the donors signed a consent form at the Banc de Teixits. The Banc de Teixits follows the principles set out in the WMA Declaration of Helsinki. The blood was carefully layered on a Ficoll-Paque gradient (Amersham, Buckinghamshire, UK) and centrifuged at 2,000 rpm for 30 min without braking. After centrifugation, peripheral blood mononuclear cells (PBMCs), in the interface between the plasma and the Ficoll-Paque gradient, were collected and washed twice with ice-cold PBS, followed by centrifugation at 2,000 rpm for 5 min. Pure CD14+ cells were isolated from PBMCs using positive selection with MACS magnetic CD14 antibody (Miltenyi Biotec). Cells were then resuspended in α-minimal essential medium (α-MEM, Glutamax no nucleosides) (Invitrogen, Carlsbad, CA, USA) containing 10% fetal bovine serum, 100 units/mL penicillin, 100 μg/mL streptomycin and antimycotic and supplemented with 25 ng/mL human M-CSF and 50 ng/mL hRANKL soluble (PeproTech EC, London, UK). Depending on the amount needed, cells were seeded at a density of 3 · 10^5^ cells/well in 96-well plates, 5 · 10^6^ cells/well in 6-well plates or 40 · 10^6^ cells in 10 mm plates and cultured for 21 days (unless otherwise noted); medium and cytokines were changed twice a week. The presence of OCs was checked by tartrate-resistant acid phosphatase (TRAP) staining using the Leukocyte Acid Phosphatase Assay Kit (Sigma-Aldrich) according to the manufacturer’s instructions. A phalloidin/DAPI stain allowed us to confirm that the populations were highly enriched in multinuclear cells, some of them containing more than 40 nuclei. We used several methods to determine that on day 21 almost 85% of the nuclei detected were ‘osteoclastic nuclei’ (in polykaryons, nuclei and not cells were quantified). OCs (TRAP-positive cells with more than three nuclei) were also analyzed at the mRNA level: upregulation of key OC markers (*TRAP/ACP5*, *CA2*, *MMP9*, and *CTSK*) and the downregulation of the MO marker *CX3CR1* were confirmed.

### Treatment of MOs with 5-aza-2-deoxycytidine

In some cases we performed monocyte-to-osteoclast differentiation experiments in the presence of different subtoxic concentrations of the DNA replication-coupled demethylating drug 5-aza-2-deoxycytidine (at 50 nM, 500 mM) for 72 h.

### Visualization of OCs with phalloidin and DAPI staining

PBMCs or pure isolated CD14+ cells were seeded and cultured in glass Lab-Tek Chamber Slides (Thermo Fisher Scientific) for 21 days in the presence of hM-CSF and hRANKL. OCs were then washed twice with PBS and fixed (3.7% paraformaldehyde, 15 min). Cells were permeabilized with 0.1% (V/V) Triton X-100 for 5 min and stained for F-actin with 5 U/mL Alexa Fluor^®^ 647-Phalloidin (Invitrogen). Cells were then mounted in Mowiol-DAPI mounting medium. Cultures were visualized by CLSM (Leica TCP SP2 AOBS confocal microscope).

### DNA methylation profiling using universal bead arrays

Infinium HumanMethylation450 BeadChips (Illumina, Inc.) were used to analyze DNA methylation. This array allows interrogating >485,000 methylation sites per sample at single-nucleotide resolution, covering 99% of RefSeq genes, with an average of 17 CpG sites per gene region distributed across the promoter, 5′UTR, first exon, gene body and 3′UTR. It covers 96% of CpG islands, with additional coverage in CpG island shores and the regions flanking them. DNA samples were bisulfite converted using the EZ DNA methylation kit (Zymo Research, Orange, CA, USA). After bisulfite treatment, the remaining assay steps were performed following the specifications and using the reagents supplied and recommended by the manufacturer. The array was hybridized using a temperature gradient program, and arrays were imaged using a BeadArray Reader (Illumina, Inc.). The image processing and intensity data extraction software and procedures were those previously described [53]. Each methylation data point is obtained from a combination of the Cy3 and Cy5 fluorescent intensities from the M (methylated) and U (unmethylated) alleles. Background intensity computed from a set of negative controls was subtracted from each data point. For representation and further analysis we used both Beta values and M values. The Beta-value is the ratio of the methylated probe intensity and the overall intensity (sum of methylated and unmethylated probe intensities). The M value is calculated as the log2 ratio of the intensities of methylated probe versus unmethylated probe. The Beta value ranges from 0 to 1 and is more intuitive and was used in heatmaps and in comparisons with DNA methylation percentages from bisulfite pyrosequencing experiments, however for statistics purposes it is more adequate the use of M values [54].

### Detection of differentially methylated CpGs

The approach to select differentially methylated CpGs was implemented in R [55], a well-known language in statistical computing. In order to process Illumina Infinium HumanMethylation450 methylation data, we used the methods supplied in limma [56], genefilter, and lumi [57] packages from Bioconductor repository. Previous to statistical analysis, a pre-process stage is applied, the main steps are: (1) color balance adjustment, that is, normalization between two color channels; (2) performing quantile normalization based on color balance adjusted data; and (3) variance filtering by IQR (interquartile range) using 0.50 for threshold value. Subsequently, for statistical analysis, eBayes moderated t-statistics test was carried out from limma package [56]. Specifically, a paired limma was performed as designed in IMA package [58]. To choose significant differences in methylated CpGs several criteria have been proposed. In this study, we considered a probe as differentially methylated if: (1) it has a fold-change >2 for hypermethylated and <0.5 hypomethylated; and (2) the statistical test was significant (*P* value <0.01 and FDR <0.05).

### Identification of genomic clusters of differentially methylated CpGs

A clustering method was applied to the differenced methylated CpGs from charm package [59]. We re-implemented the code to invoke the main clustering function using genomic CpG localization: identify differentially methylated regions (DMRs) by grouping differentially methylated probes (DMPs). The maximum allowable gap between probe positions for probes to be clustered into the same region was set to 500 bp. It has been shown that in many cases methylation changes are observed over a range of CpGs, which may be identified for instance at shores close to transcription starting sites. We considered that DMR are more robust signals than DMPs. In this analysis, the considered list of CpGs attains a *P* value <0.01 and FDR <0.05.

### Bisulfite sequencing and pyrosequencing

We used bisulfite pyrosequencing to validate CpG methylation changes resulting from the analysis with the Infinium HumanMethylation450 BeadChips. Bisulfite modification of genomic DNA isolated from MOs, OCs, and samples from time course or PU.1-knockdown experiments was carried out as described by Herman et al. [60]. A total of 2 μL of the converted DNA (corresponding to approximately 20–30 ng) were then used as a template in each subsequent PCR. Primers for PCR amplification and sequencing were designed with the PyroMark^®^ Assay Design 2.0 software (Qiagen). PCRs were performed with the HotStart Taq DNA polymerase PCR kit (Qiagen), and the success of amplification was assessed by agarose gel electrophoresis. PCR products were pyrosequenced with the Pyromark™ Q24 system (Qiagen). In the case of repetitive elements (Sat2, D4Z4, NBL2, 18S rRNA, and 28 rRNA) we performed standard bisulfite sequencing of a minimum of 10 clones. Results from bisulfite pyrosequencing and sequencing of multiple clones are presented as a percentage of methylation. All primer sequences are listed in Additional file 11. Raw data for bisulfite sequencing of all samples is presented in Additional file 4.

### Gene expression data analysis and comparison of DNA expression data *versus* DNA methylation data

In order to compare expression data *versus* methylation data, we used CD14+ and OC expression data from ArrayExpress database [61] under the accession name (E-MEXP-2019) from a previous publication [32]. Affymetrix GeneChip Human Genome U133 Plus 2.0 expression data was processed using limma [56] and affy [62] packages from bioconductor. The preprocessing stage is divided into three major steps: (1) background correction; (2) normalization; and (3) reporter summarization. Here, the expresso function in the affy package was chosen for preprocessing. Thus, the RMA method [63] was applied for background correction. Then, a quantile normalization was performed. In addition, we introduced a specific step for PM (perfect match probes) adjustment, utilizing the PM-only model based expression index (option ‘pmonly’). And finally, for summarization step, the median polish method was taken. Next, as previously in the methylation analysis, a variance filtering by IQR using 0.50 for threshold value was executed. After preprocessing, a statistical analysis was applied, using eBayes moderated t-statistics test from limma package. Subsequently, expression genes matching to methylated genes were studied. Genes differentially expressed between MOs and Mo-OCs groups were selected with a criteria of *P* value <0.01 and FDR <0.05 as calculated by Benjamini-Hochberg and a fold-change of expression >2 or <0.5. Validation of expression data was performed by quantitative RT-PCR. All primer sequences are listed in Additional file 11.

### Gene ontology analysis

Gene ontology (GO) was analyzed with the FatiGO tool [64], which uses Fisher’s exact test to detect significant over-representation of GO terms in one of the sets (list of selected genes) with respect to the other (the rest of the genome). Multiple test correction to account for the multiple hypotheses tested (one for each GO term) was applied to reduce false-positive results. GO terms with adjusted values of *P* <0.05 were considered significant.

### Analysis of transcription factor binding

We used the STORM algorithm [65] to identify potential over-representation of transcription factor motifs in the 500-bp region around the center of the hypomethylated/hypermethylated CpG sites (as well as for all other CpGs-containing probes contained in the array) assuming cutoff values of *P* = 0.00002 (for hypo-/hypermethylated probes) and 0.00001 (for all other probes), using position frequency matrices (PFMs) from the TRANSFAC database (Professional version, release 2009.4) [66]. Enrichment analysis of predicted TF in the probes of significant hypomethylated probes (*n* = 421) was conducted using GiTools [67,68]. We calculated two-tailed probabilities, and a final adjusted FDR *P* value (with 0.25 cutoff) was considered statistically significant.

We downloaded PU.1 ChIPseq data for CD14+ MOs generated by Michael Rehli’s laboratory [1] from the Gene Expression Omnibus (GSE31621). The genomic locations of the calculated peaks were mapped to GRCh37.p10 human alignment obtained from Biomart [69], by using bedtools (intersect function) in order to obtain the PU.1 occupied genes. To determine whether a given CpG (from the Illumina bead array) was positive for PU.1 binding, we used the same 500-bp window used for TRANSFAC analysis.

### Graphics and heatmaps

All graphs were created using Prism5 Graphpad. Heatmaps were generated from the expression or methylation data using the Genesis program (Graz University of Technology) [70].

### BrdU proliferation assays

BrdU was used at a final concentration of 300 μm, as previously described. On the days specified, BrdU pulsing solution was added to each well for 2 to 4 days. For confocal microscopy of monocyte-to-osteoclast differentiation samples, CD14+ cells were seeded on Millicell EZ 8-well glass slides (Millipore) and cultured in differentiation media. At different times BrdU was added to the medium and after 2 to 4 days cells were fixed (4% paraformaldehyde, 30 min, RT), permeabilized (PBS-BSA-Triton X-100 0.8% (PBT), 10 min, RT) and treated with HCl 2 N for 30 min. After DNA opening, HCl was neutralized by two 5-min washes with NaBo (0.1 M, pH 8.5) and two 5-min washes with PBT. Cells were incubated with anti-BrdU antibody (18 h at 4°C, 1:1,000 dilution) and an anti-mouse Alexa-568 conjugated antibody was added to visualize the BrdU-positive nuclei. A phalloidin incubation step and Mowiol-DAPI mounting media were used.

### Transfection of primary human MOs

We used two different Silencer^®^ select pre-designed siRNAs against human PU.1 (one targeting exon 2 and another targeting the 3′UTR) and a Silencer^®^ select negative control to perform PU.1 knockdown experiments in peripheral blood MOs. We used Lipofectamine RNAiMAX Transfection Reagent (Invitrogen) for efficient siRNA transfection. mRNA and protein levels were examined by quantitative RT-PCR and western blot at 1, 2, 4, and 6 days after siRNA transfection. In this case MO samples were prepared by incubating PBMCs in plates in α-MEM without serum for 30 min and washing out the unattached cells. Under these conditions over 80% are MOs. This alternative protocol was used for increased viability following transfection. These experiments were performed with three biological replicates.

### Chromatin immunoprecipitation (ChIP) assays and immunoprecipitation experiments

Immunoprecipitation was performed by standard procedures in CD14+ cells at 0, 2, and 4 days after treatment with M-CSF and RANKL. Cell extracts were prepared in 50 mM Tris–HCl, pH 7.5, 1 mM EDTA, 150 mM NaCl, 1% Triton-X-100, and protease cocktail inhibitors (Complete, Roche Molecular Biochemicals). Cellular extracts and samples from immunoprecipitation experiments were electrophoresed and western blotted following standard procedures.

For chromatin immunoprecipitation (ChIP) assays, CD14+ at 0, 2, and 4 days after treatment with M-CSF and RANKL were cross-linked with 1% formaldehyde and subjected to immunoprecipitation after sonication. ChIP experiments were performed as described [44]. Analysis was performed by real-time quantitative PCR. Data are represented as the ratio of the bound fraction over the input for each specific factor. We used a mouse monoclonal antibody against the TET2 N-t for ChIPs and a rabbit polyclonal antibody against TET2 for western blot. For DNMT3b we used a rabbit polyclonal against amino acids 1–230 of human DNMT3b (sc-20704, Santa Cruz Biotechnology). We also used a rabbit polyclonal against the C-t of PU.1 (sc-352, Santa Cruz Biotechnology), a rabbit polyclonal against the N-t of c-Fos (sc-52, Santa Cruz Biotechnology) and a rabbit polyclonal against the C-t of NF-kB p65 (sc-372, Santa Cruz Biotechnology). IgG was used as a negative control. Primer sequences were designed to contain either predicted or known TF binding (from TRANSFAC or ChIPseq data) as close as possible from the CpG undergoing methylation changes. Primer sequences are shown in Additional file 11. These experiments were performed with three biological replicates.

### 5hmC detection

5hmC was analyzed using the Quest 5hmC Detection system (Zymo). Genomic DNA was treated with a specific 5hmC glucosyltransferase (GT) or left untreated (No GT, 0% 5hmC). DNA was then digested with MspI (100U) at 37°C overnight, followed by column purification. The MspI-resistant fraction (bearing the glucosile group, and therefore the original 5hmC) was quantitated by qPCR using primers designed around at least one MspI site (CCGG), and normalized to the amplification of the same region in the original DNA input. The amplification obtained in the untreated (no GT, MspI sensible) was then substracted to the samples in order to calculate the level of 0% 5hmC. The resulting values were the percentage of 5hmC present in each of the samples. Primer sequences are shown in Additional file 11.

### Amplification of unmethylated Alus

This method, aiming at the amplification of unmethylated Alus (AUMA), was performed as described [31,39]. Products were resolved on denaturing sequencing gels. Bands were visualized by silver staining the gels. AUMA fingerprints were visually checked for methylation differences between bands in different samples.

## Abbreviations

5azadC: 5-aza-2′-deoxycytidine; 5hmC: 5-hydroxymethylcytosine; 5mC: 5-methylcytosine; AUMA: Amplification of unmethylated Alu repeats.

## Competing interests

The authors declare that they have no competing interests.

## Authors’ contributions

LR and EB conceived experiments; LR, MG, JR-U, and HH performed experiments; AI and JMU performed biocomputing analysis; LR, JMU, JC, KH, CGV, and EB analyzed the data; EB wrote the paper. All authors read and approved the final manuscript.

## Supplementary Material

Additional file 1 **M-CSF and RANKL-induced monocyte-to-osteoclast differentiation. (A)** Visualization of the formation of the actin ring and the generation of polykaryons in monocyte (MO) to osteoclast (OC) differentiation with phalloidin and DAPI staining. **(B)** TRAP (Tartrate resistant acid phosphatase-OC marker) staining in MO and OC preparations, showing this activity only in OCs. Determination of the typical percentage of osteoclastic nuclei present in the preparations used for the experiments; over 84% efficiency was achieved at 21 days. **(C)** Upregulation of OC specific markers (*CA2*, *CTSK*, *MMP9*, *ACP5*) was checked by qPCR; downregulation of a monocyte specific gene (*CX3CR1*) was also monitored. Click here for file

Additional file 2 List of hypomethylated and hypermethylated genes during monocyte to osteoclast differentiation (FC <0.5 (hypomethylated, sheet 1) or FC >2. Click here for file

Additional file 3 **(A) Scatterplots showing DNA methylation profiles of matching MO/OC pairs.** Genes with significant differences (FC >2, FDR <0.05) in averaged results from three samples are highlighted in red (hypermethylated) or blue (hypomethylated). Three panels corresponding for each of the three individual comparisons of MO/OC pairs (D1, D2, and D3) are shown. **(B)** Bisulfite sequencing analysis of repetitive sequences performed on monocytes (day 0) and osteoclasts (day 21) from three different donors (donor A, donor B, and donor C), showing no relevant differences in the DNA methylation levels. **(C)** AUMA (amplification of unmethylated Alus) analysis of two independent monocyte-to-osteoclast differentiation experiments. Graphs correspond to the scanned intensities of the bands obtained with two different sets of primers. No significant differences are observed. Click here for file

Additional file 4 **Individual raw data corresponding to bisulfite pyrosequencing and standard bisulfite sequencing of individual MO and OC samples (Figure **1**E), time course methylation data (Figure **2**D, E) and PU.1 siRNA experiments (Figure **5**D).** Data are presented as supplied by PyroMark^®^ Assay Design Software 2.0 for PyroMark Q96 MD (Qiagen), which automatically generates methylation percentages in a datasheet format. Click here for file

Additional file 5 **Clusters of consecutive CpGs hypomethylated (-) or hypermethylated (+) in OC *vs.* MO.** Click here for file

Additional file 6 Differentially expressed genes between Mos, OC samples at 5 days and OC samples at 20 days after RANKL/M-CSF stimulation (FC >2, FC <0.5; FDR <0.05). Click here for file

Additional file 7 List of genes with an inverse relationship between DNA methylation and expression change (FC <0.5 orFC >2; FDR <0.05 for both DNA methylation and expression data). Click here for file

Additional file 8 **(A) Scheme showing the BrdU pulses added to monocytes differentiating into osteoclasts. (B)** Representative immunofluorescence images at the selected time points showing BrdU positive cells. **(C)** Representation of the time scale where DNA demethylation occurs during osteoclast differentiation, together with the cell division observed at later time points. Click here for file

Additional file 9 **Osteoclast differentiation scheme showing transcription factors that are known to be involved in monocyte-to-osteoclast differentiation.** We have in red or blue the presence of binding motifs for those factors (according to TRANSFAC analysis) among the sequences surrounding the CpGs that become hypo- or hypermethylated. Those arising from our analysis are highlighted in red and blue (associated with hypermethylation and hypomethylation, respectively). Click here for file

Additional file 10 **(A) ChIP assays showing the effects of PU.1 downregulation in its recruitment, together with TET2 and DNMT3b binding to the same genes.** Data were obtained at 0, 2, and 6 days after M-CSF/RANL stimulation. **(B)** We have used the MYOD1 promoter as a negative control. **(C)** Effects of PU.1 downregulation on expression and methylation of PU.1-target gene TM7SF4. Click here for file

Additional file 11 List of primers. Click here for file

## Data access

Methylation array data for this publication has been deposited in NCBI’s Gene Expression Omnibus and is accessible through GEO Series accession number GSE46648.
