# Supplementary figures and images for: PU.1 target genes undergo Tet2-coupled demethylation and DNMT3b-mediated methylation in monocyte-to-osteoclast differentiation
Source: Genome Biol. 2013 Sep 12;14(9):R99. doi: 10.1186/gb-2013-14-9-r99 (PMC4054781; doi:10.1186/gb-2013-14-9-r99)

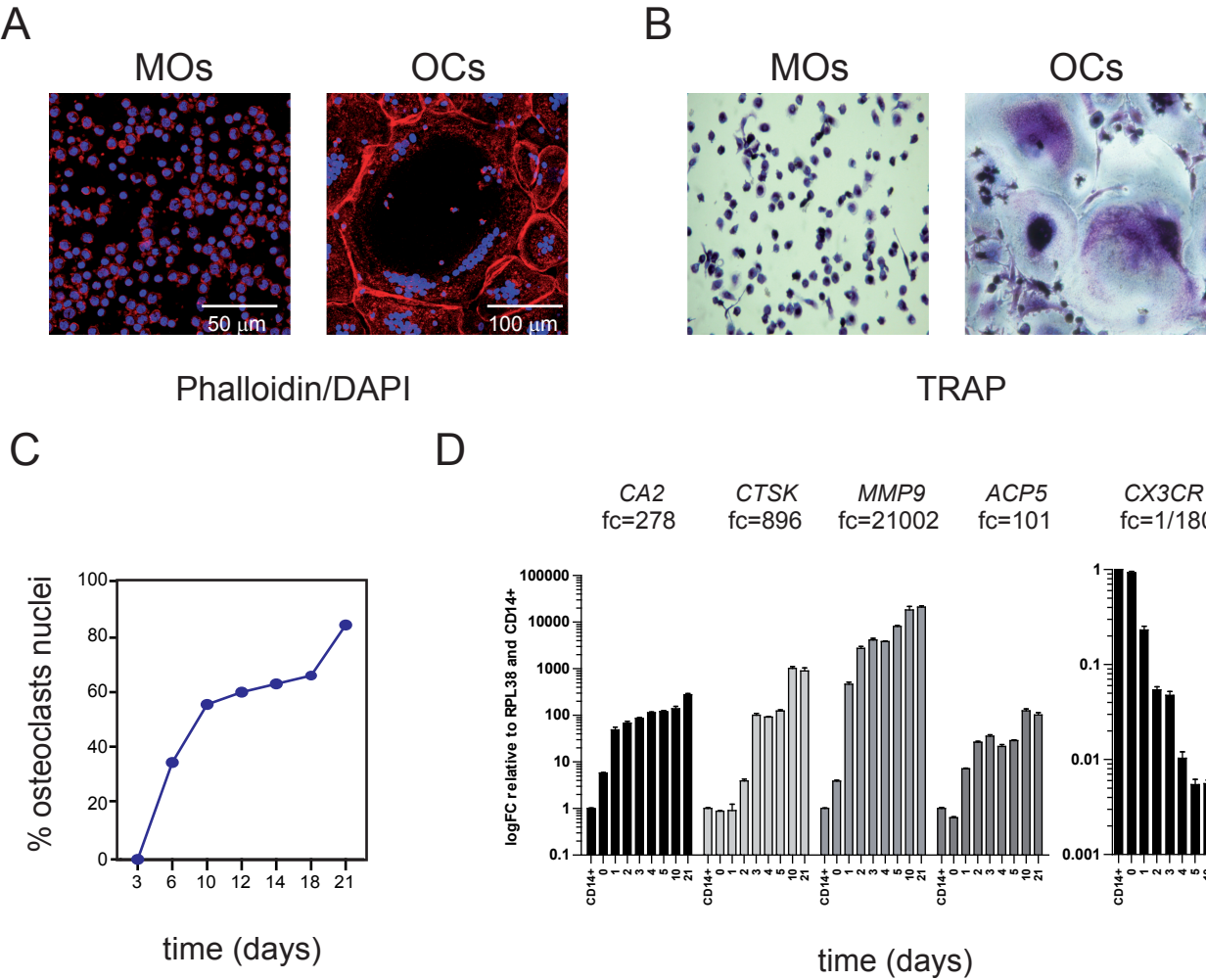

Supplement: Additional file 1 — M-CSF and RANKL-induced monocyte-to-osteoclast differentiation. (A) Visualization of the formation of the actin ring and the generation of polykaryons in monocyte (MO) to osteoclast (OC) differentiation with phalloidin and DAPI staining. (B) TRAP (Tartrate resistant acid phosphatase-OC marker) staining in MO and OC preparations, showing this activity only in OCs. Determination of the typical percentage of osteoclastic nuclei present in the preparations used for the experiments; over 84% efficiency was achieved at 21 days. (C) Upregulation of OC specific markers (CA2, CTSK, MMP9, ACP5) was checked by qPCR; downregulation of a monocyte specific gene (CX3CR1) was also monitored. [file gb-2013-14-9-r99-S1.pdf]

# Additional file 3

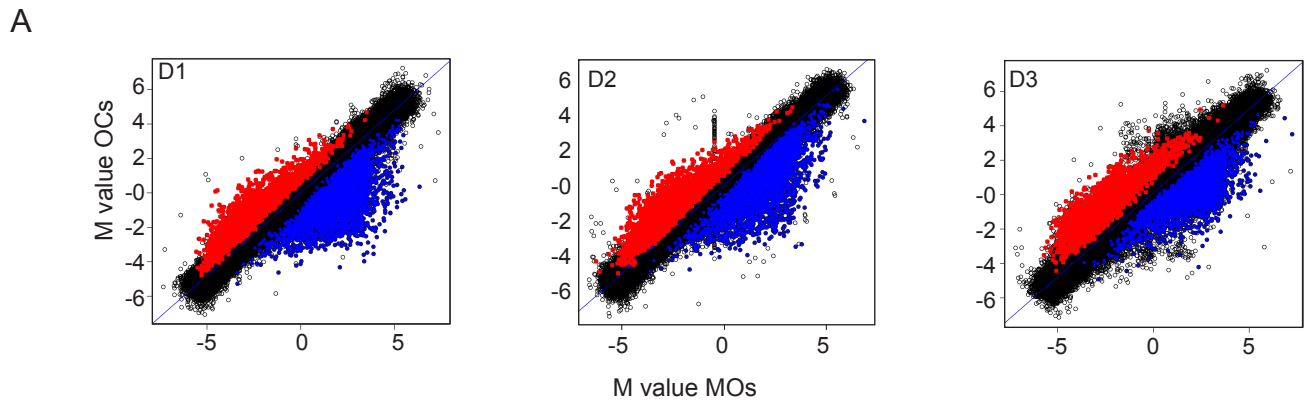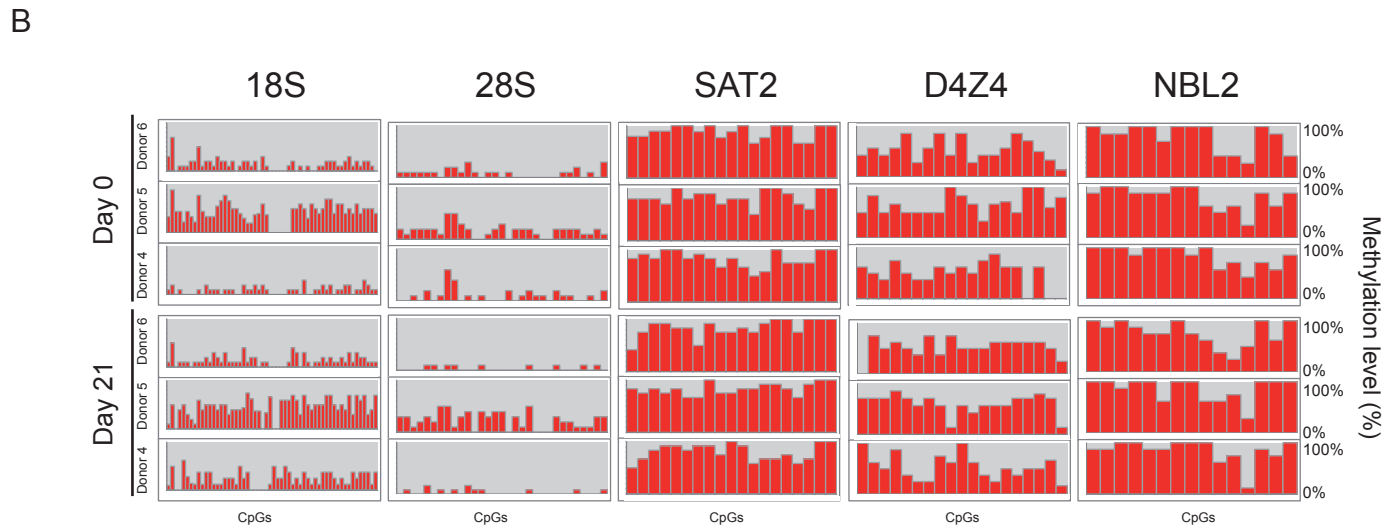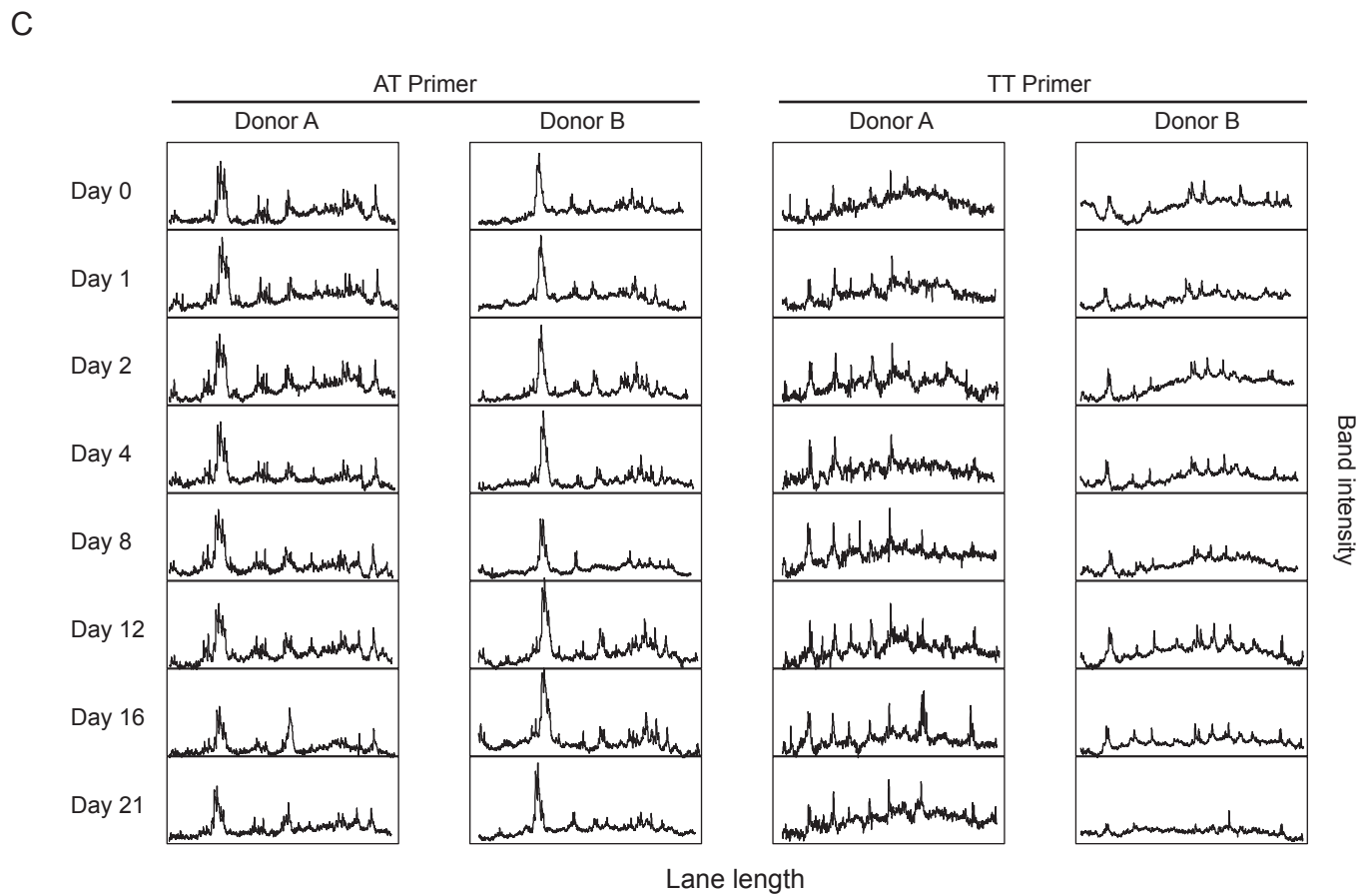

Supplement: Additional file 3 — (A) Scatterplots showing DNA methylation profiles of matching MO/OC pairs. Genes with significant differences (FC >2, FDR <0.05) in averaged results from three samples are highlighted in red (hypermethylated) or blue (hypomethylated). Three panels corresponding for each of the three individual comparisons of MO/OC pairs (D1, D2, and D3) are shown. (B) Bisulfite sequencing analysis of repetitive sequences performed on monocytes (day 0) and osteoclasts (day 21) from three different donors (donor A, donor B, and donor C), showing no relevant differences in the DNA methylation levels. (C) AUMA (amplification of unmethylated Alus) analysis of two independent monocyte-to-osteoclast differentiation experiments. Graphs correspond to the scanned intensities of the bands obtained with two different sets of primers. No significant differences are observed. [file gb-2013-14-9-r99-S3.pdf]

A

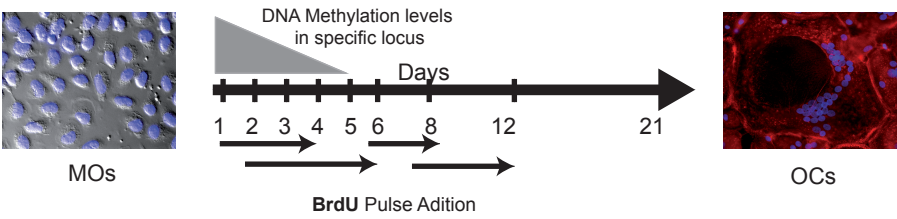

B

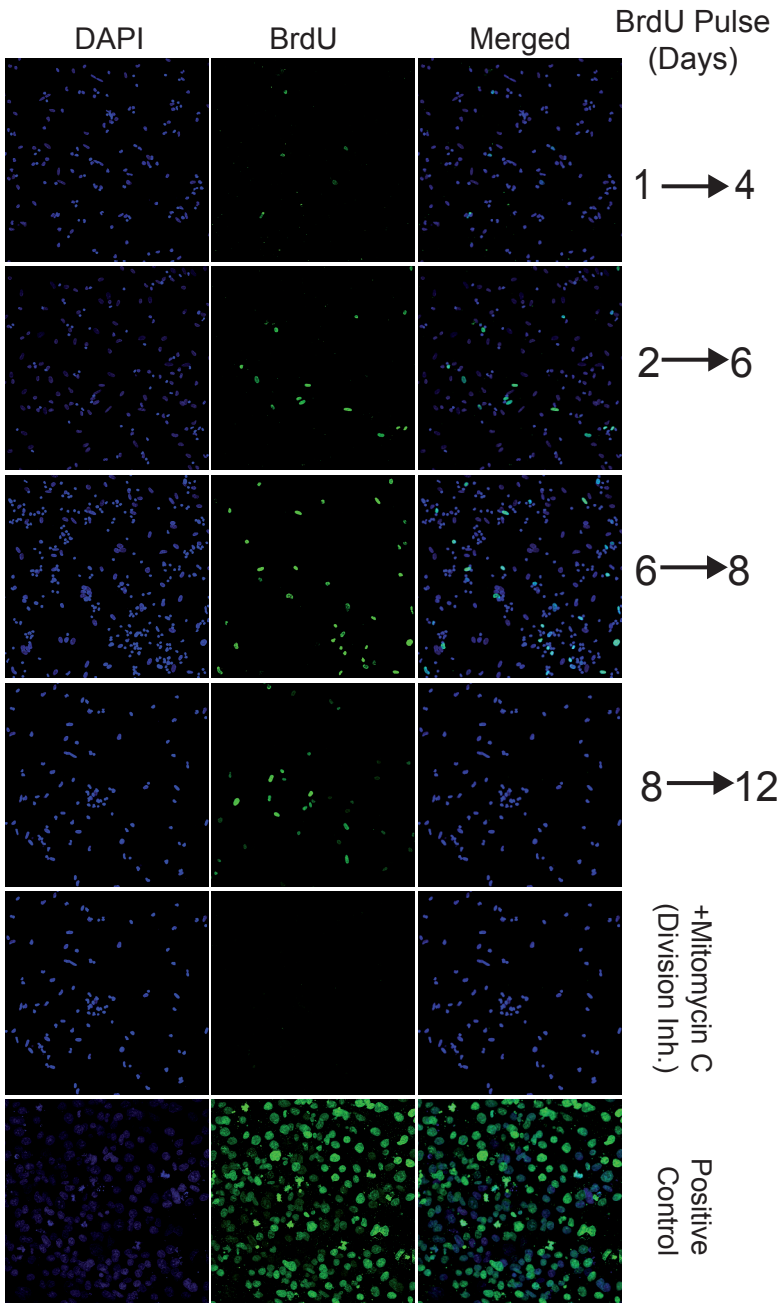

C

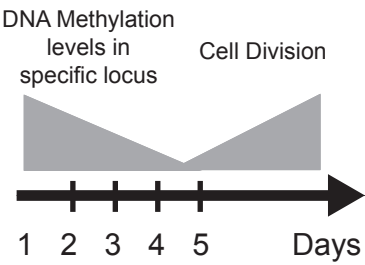

Supplement: Additional file 8 — (A) Scheme showing the BrdU pulses added to monocytes differentiating into osteoclasts. (B) Representative immunofluorescence images at the selected time points showing BrdU positive cells. (C) Representation of the time scale where DNA demethylation occurs during osteoclast differentiation, together with the cell division observed at later time points. [file gb-2013-14-9-r99-S8.pdf]

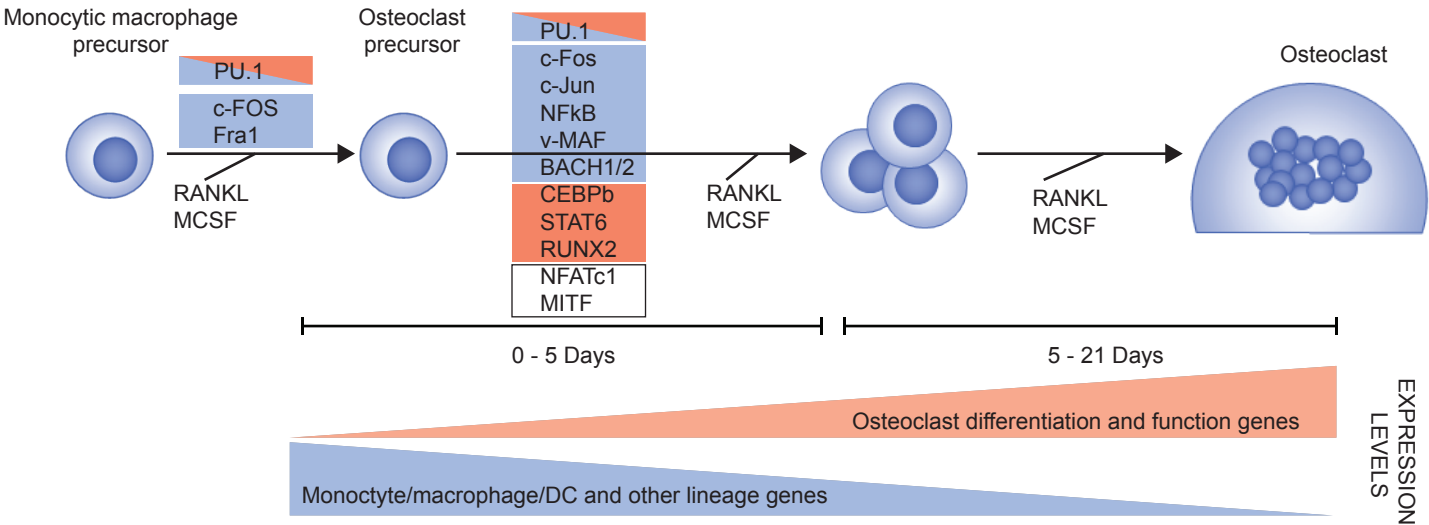

Supplement: Additional file 9 — Osteoclast differentiation scheme showing transcription factors that are known to be involved in monocyte-to-osteoclast differentiation. We have in red or blue the presence of binding motifs for those factors (according to TRANSFAC analysis) among the sequences surrounding the CpGs that become hypo- or hypermethylated. Those arising from our analysis are highlighted in red and blue (associated with hypermethylation and hypomethylation, respectively). [file gb-2013-14-9-r99-S9.pdf]

A

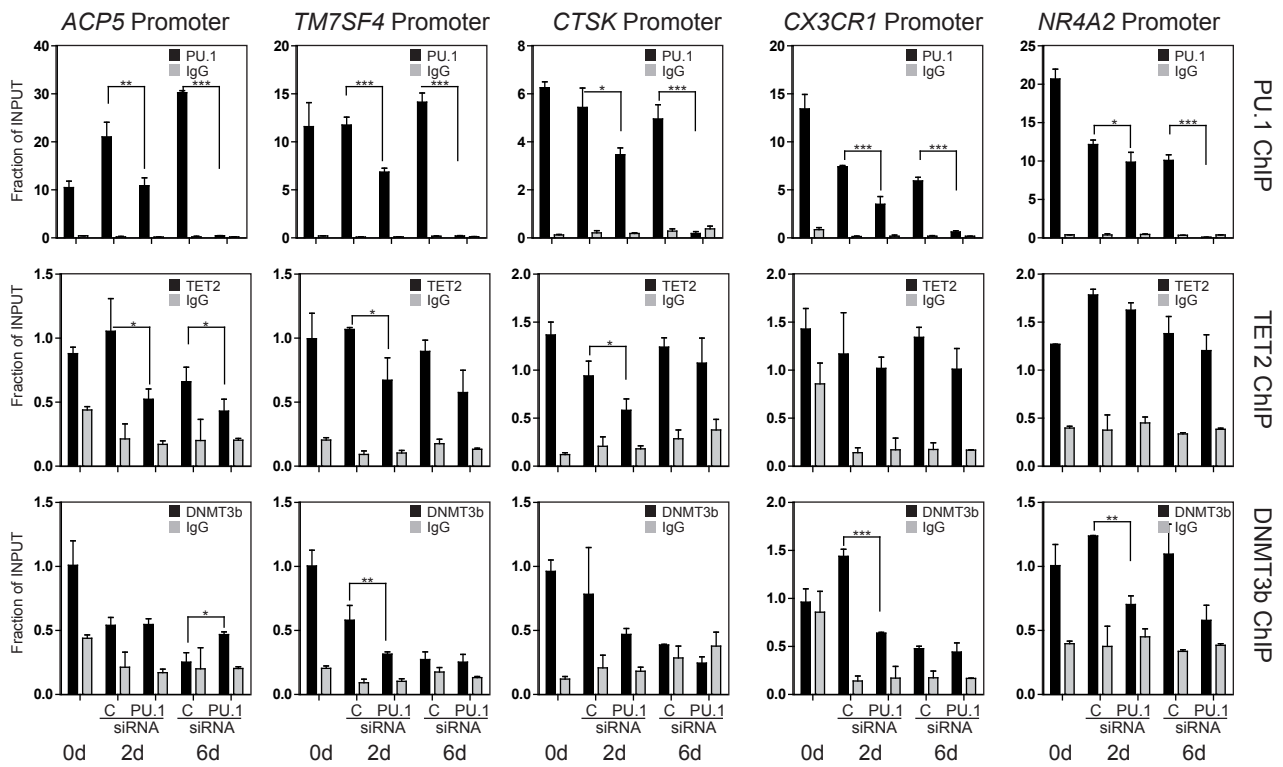

B

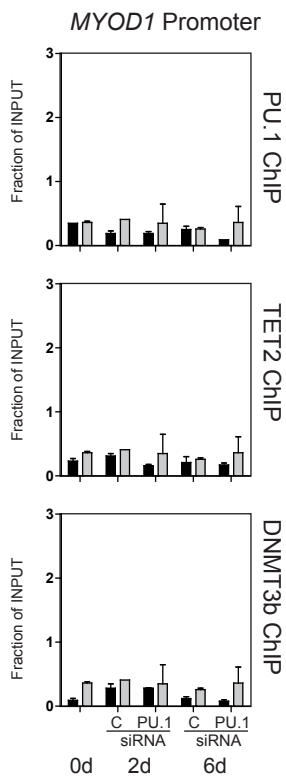

C

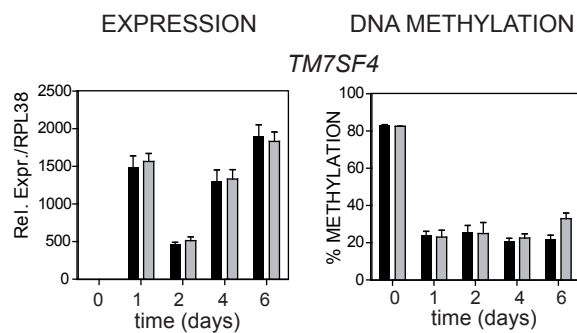

Supplement: Additional file 10 — (A) ChIP assays showing the effects of PU.1 downregulation in its recruitment, together with TET2 and DNMT3b binding to the same genes. Data were obtained at 0, 2, and 6 days after M-CSF/RANL stimulation. (B) We have used the MYOD1 promoter as a negative control. (C) Effects of PU.1 downregulation on expression and methylation of PU.1-target gene TM7SF4. [file gb-2013-14-9-r99-S10.pdf]
