# Supplementary material for: PU.1 target genes undergo Tet2-coupled demethylation and DNMT3b-mediated methylation in monocyte-to-osteoclast differentiation
Source: Genome Biol. 2013 Sep 12;14(9):R99. doi: 10.1186/gb-2013-14-9-r99 (PMC4054781; doi:10.1186/gb-2013-14-9-r99)
Supplement: Additional file 4 — Individual raw data corresponding to bisulfite pyrosequencing and standard bisulfite sequencing of individual MO and OC samples (Figure 1E), time course methylation data (Figure 2D, E) and PU.1 siRNA experiments (Figure 5D). Data are presented as supplied by PyroMark® Assay Design Software 2.0 for PyroMark Q96 MD (Qiagen), which automatically generates methylation percentages in a datasheet format. [file gb-2013-14-9-r99-S4.docx]

**Additional file 4. Individual raw data corresponding to bisulfite pyrosequencing and standard bisulfite sequencing of individual MO and OC samples of individual MO and OC samples (Figure 1E). time course methylation data (Figure 2D. E) and PU.1 siRNA experiments (Figure 5D). Pyrosequencing data are presented as supplied by PyroMark® Assay Design Software 2.0 for PyroMark Q96 MD (Qiagen), which automatically generates methylation percentages in a datasheet format.**

**Bisulfite pyrosequencing of individual MO and OC samples (Figure 1E):**

| **CTSK** | **MOs** | **OCs** |
| --- | --- | --- |
| **D1** | 94 | 6 |
| **D2** | 90 | 9 |
| **D3** | 93 | 26 |
| **D4** | 93 | 18 |
| **D5** | 93 | 11 |

Illumina ID: cg11946165

CpG Sequenced: TCTTTAAAAGTAACCAAAAACAGCAGTCCTGGTTATTTATGACAGCACTTGAATCAATGC[CG]TAAGTTCTGATGGACTCACATGTGACTCTGTTGCTAAAACTCTCAGGTGGTGGGATGCCC

| **ACP5** | **MOs** | **OCs** |  | **MOs** | **OCs** |  | **MOs** | **OCs** |
| --- | --- | --- | --- | --- | --- | --- | --- | --- |
| **D1** | 82 | 45 |  | 85 | 9 |  | 100 | 6 |
| **D2** | 83 | 12 |  | 87 | 7 |  | 100 | 5 |
| **D3** | 81 | 38 |  | 84 | 22 |  | 76 | 23 |
| **D4** | 81 | 35 |  | 89 | 15 |  | 74 | 14 |
| **D5** | 84 | 29 |  | 82 | 10 |  | 72 | 8 |

Illumina ID: cg21207418

CpG Sequenced: CTCGGCCCACACAGCCTCCGGGTGGACCTGCAGGGGCCTGTTTGTGCTGTAGGCTTGACA[CG]TCCAGGTATCTCTGTGTGTCTGTGTATCTCAGTGTGAGTGTGTGTGTGTGTGCACACTTG

| **TM7SF4/DC STAMP** | **MOs** | **OCs** |
| --- | --- | --- |
| **D1** | 87 | 9 |
| **D2** | 88 | 18 |
| **D3** | 82 | 33 |
| **D4** | 85 | 15 |
| **D5** | 87 | 9 |

Illumina ID: cg01136183

CpG Sequenced: AAGGAACCCCATCCAGTCCAGCCGGTTGGCTTGCTCCTCCCCTCCTCCCACTCCAGTTCA[CG]CTCCAGCCCACTGAAGAGTGGTGCCCACCCCTAGGCCCCTGCCTAAATGGCTCTTCCAGA

| **CX3CR1** | **MOs** | **OCs** |
| --- | --- | --- |
| **D1** | 24 | 52 |
| **D2** | 26 | 56 |
| **D3** | 34 | 63 |
| **D4** | 32 | 65 |
| **D5** | 22 | 55 |

Illumina ID: cg04569233

CpG Sequenced:

TAGCTGTCCACTGCTCCACCCCACCCACAGGTACCCAACTAGTCCTGTGCACCTACCTGG[CG]TGGACTGCCAAGGGAACCTCTGGATCTGCCAGTCAGCCACCCTGTCCTGCTCAGACTTTA

| **FOXP1** | **MOs** | **OCs** |
| --- | --- | --- |
| **D1** | 17 | 41 |
| **D2** | 7 | 25 |
| **D3** | 6 | 53 |
| **D4** | 10 | 46 |
| **D5** | 8 | 31 |

Illumina ID: cg02520804

CpG Sequenced:

TTGGGTAGCATTCTCCTCATAAAGAAGGATACATTAAAAAAAATAACTTGTTTCGCGACT[CG]GCATCCATAAGGAACTCAAATGCTGCCCAGAGAGGGGCTGAGTATTTCCTTCCAAGTGAG

**Bisulfite sequencing of Repetitive regions in individual MO and OC samples (Figure 1G):**

| **18S** | MONOCYTES | |  | OSTEOLASTS | |  |
| --- | --- | --- | --- | --- | --- | --- |
| %Meth | MOs_D4 | MOs_D5 | MOs_D6 | OCs_D4 | OCs_D5 | OCs_D6 |
| CpG1 | 30.00 | 33.33 | 10.00 | 10.00 | 10.00 | 10.00 |
| CpG2 | 70.00 | 88.89 | 20.00 | 50.00 | 50.00 | 50.00 |
| CpG3 | 0.00 | 44.44 |  |  |  |  |
| CpG4 | 10.00 | 44.44 | 10.00 | 10.00 | 40.00 |  |
| CpG5 | 10.00 | 22.22 | 0.00 | 10.00 | 50.00 | 62.50 |
| CpG6 | 10.00 | 44.44 | 0.00 | 10.00 | 30.00 | 28.57 |
| CpG7 | 20.00 | 33.33 | 0.00 | 0.00 | 20.00 | 14.29 |
| CpG8 | 20.00 | 22.22 | 0.00 | 10.00 | 10.00 | 12.50 |
| CpG9 | 50.00 | 77.78 | 10.00 | 10.00 | 60.00 | 37.50 |
| CpG10 | 10.00 | 44.44 | 0.00 | 10.00 | 40.00 | 12.50 |
| CpG11 | 20.00 | 33.33 | 20.00 | 20.00 | 50.00 | 37.50 |
| CpG12 | 20.00 | 33.33 | 10.00 | 10.00 | 50.00 | 37.50 |
| CpG13 | 10.00 | 33.33 | 10.00 | 30.00 | 50.00 | 12.50 |
| CpG14 | 30.00 | 55.56 | 10.00 | 20.00 | 40.00 | 12.50 |
| CpG15 | 20.00 | 66.67 | 0.00 | 10.00 | 50.00 | 12.50 |
| CpG16 | 20.00 | 77.78 | 10.00 | 30.00 | 50.00 | 25.00 |
| CpG17 | 10.00 | 66.67 | 10.00 | 10.00 | 30.00 | 25.00 |
| CpG18 | 20.00 | 50.00 | 10.00 | 10.00 | 40.00 | 25.00 |
| CpG19 | 0.00 | 50.00 | 0.00 | 10.00 | 40.00 | 12.50 |
| CpG20 | 10.00 | 40.00 | 0.00 | 10.00 | 40.00 | 50.00 |
| CpG21 | 20.00 | 30.00 | 20.00 | 40.00 | 44.44 | 25.00 |
| CpG22 | 20.00 | 20.00 | 10.00 | 10.00 | 75.00 | 37.50 |
| CpG23 | 10.00 | 20.00 | 10.00 | 20.00 | 62.50 |  |
| CpG24 | 20.00 | 37.50 | 20.00 | 20.00 | 37.50 |  |
| CpG25 | 0.00 | 40.00 | 10.00 | 0.00 | 37.50 |  |
| CpG26 | 30.00 | 60.00 | 20.00 | 10.00 |  |  |
| CpG27 | 10.00 | 37.50 | 10.00 | 10.00 | 33.33 |  |
| CpG28 |  |  |  |  | 66.67 | 12.50 |
| CpG29 |  |  |  |  |  | 50.00 |
| CpG30 |  |  |  |  |  | 25.00 |
| CpG31 |  |  |  |  | 60.00 | 25.00 |
| CpG32 |  |  |  |  | 60.00 | 50.00 |
| CpG33 | 11.11 |  | 10.00 | 10.00 | 60.00 | 37.50 |
| CpG34 | 20.00 | 50.00 | 10.00 | 40.00 | 70.00 | 12.50 |
| CpG35 | 0.00 | 50.00 | 10.00 | 30.00 | 60.00 | 25.00 |
| CpG36 | 10.00 | 60.00 | 0.00 | 0.00 | 30.00 | 12.50 |
| CpG37 | 0.00 | 50.00 | 30.00 | 30.00 | 70.00 | 37.50 |
| CpG38 | 10.00 | 30.00 | 0.00 | 10.00 | 50.00 | 25.00 |
| CpG39 | 0.00 | 60.00 | 0.00 | 0.00 | 40.00 | 12.50 |
| CpG40 | 0.00 | 50.00 | 10.00 | 10.00 | 50.00 | 25.00 |
| CpG41 | 10.00 | 40.00 | 10.00 | 10.00 | 50.00 | 12.50 |
| CpG42 | 10.00 | 50.00 | 20.00 | 20.00 | 50.00 | 37.50 |
| CpG43 | 20.00 | 70.00 | 10.00 | 10.00 | 70.00 | 37.50 |
| CpG44 | 20.00 | 70.00 | 20.00 | 20.00 | 70.00 | 37.50 |
| CpG45 | 20.00 | 40.00 | 0.00 | 10.00 | 50.00 | 25.00 |
| CpG46 | 10.00 | 60.00 | 10.00 | 10.00 | 40.00 | 12.50 |
| CpG47 | 10.00 | 60.00 | 10.00 | 20.00 | 50.00 | 25.00 |
| CpG48 | 20.00 | 40.00 | 20.00 | 10.00 | 40.00 | 12.50 |
| CpG49 | 30.00 | 50.00 | 20.00 | 30.00 | 70.00 | 12.50 |
| CpG50 | 10.00 | 40.00 | 0.00 | 10.00 | 30.00 | 37.50 |
| CpG51 | 20.00 | 60.00 | 10.00 | 30.00 | 70.00 | 25.00 |
| CpG52 | 10.00 | 40.00 | 10.00 | 20.00 | 60.00 | 37.50 |
| CpG53 | 20.00 | 50.00 | 30.00 | 20.00 | 70.00 | 37.50 |
| CpG54 | 20.00 | 50.00 | 20.00 | 10.00 | 30.00 | 37.50 |
| CpG55 | 10.00 | 50.00 | 10.00 | 10.00 | 40.00 | 12.50 |
| CpG56 | 0.00 | 40.00 | 10.00 | 10.00 | 70.00 | 37.50 |

| **28S** | MONOCYTES | |  | OSTEOLASTS | |  |
| --- | --- | --- | --- | --- | --- | --- |
| %Meth | MOs_D4 | MOs_D5 | MOs_D6 | OCs_D4 | OCs_D5 | OCs_D6 |
| CpG1 | 10.00 | 20.00 |  |  | 30.00 |  |
| CpG2 | 10.00 | 10.00 |  |  | 30.00 | 8.33 |
| CpG3 | 10.00 | 20.00 | 10.00 |  | 10.00 |  |
| CpG4 | 10.00 | 20.00 |  |  | 20.00 |  |
| CpG5 | 10.00 | 20.00 | 20.00 | 10.00 | 30.00 | 16.67 |
| CpG6 | 10.00 | 20.00 |  | 10.00 | 20.00 |  |
| CpG7 |  | 10.00 | 10.00 |  | 50.00 | 8.33 |
| CpG8 | 20.00 | 50.00 | 60.00 | 10.00 | 50.00 |  |
| CpG9 | 20.00 | 50.00 | 40.00 | 10.00 | 10.00 | 8.33 |
| CpG10 | 10.00 | 30.00 |  |  | 30.00 |  |
| CpG11 | 30.00 | 20.00 | 10.00 |  | 40.00 | 16.67 |
| CpG12 | 10.00 |  |  |  |  | 8.33 |
| CpG13 |  |  | 10.00 | 10.00 | 40.00 | 8.33 |
| CpG14 | 10.00 | 10.00 |  |  | 30.00 |  |
| CpG15 | 10.00 | 20.00 |  |  | 40.00 |  |
| CpG16 |  | 30.00 |  |  | 40.00 |  |
| CpG17 | 10.00 |  | 20.00 |  |  |  |
| CpG18 |  | 20.00 |  |  | 30.00 |  |
| CpG19 |  | 20.00 | 10.00 |  | 10.00 | 0.00 |
| CpG20 |  | 20.00 | 20.00 | 10.00 | 50.00 | 8.33 |
| CpG21 |  | 10.00 | 10.00 |  |  |  |
| CpG22 |  |  | 10.00 |  |  |  |
| CpG23 |  |  |  |  |  |  |
| CpG24 |  | 20.00 | 20.00 | 10.00 | 30.00 |  |
| CpG25 | 10.00 | 20.00 | 10.00 |  | 20.00 |  |
| CpG26 | 10.00 | 20.00 | 10.00 |  | 20.00 |  |
| CpG27 | 20.00 | 20.00 |  |  | 10.00 | 8.33 |
| CpG28 |  | 10.00 |  | 10.00 | 10.00 |  |
| CpG29 | 10.00 | 10.00 | 10.00 |  | 10.00 |  |
| CpG30 |  | 20.00 |  | 10.00 | 30.00 |  |
| CpG31 | 30.00 | 10.00 | 20.00 |  | 30.00 | 8.33 |

| **SAT2** | MONOCYTES |  |  | OSTEOLASTS |  |  |
| --- | --- | --- | --- | --- | --- | --- |
| %Meth | MOs_D4 | MOs_D5 | MOs_D6 | OCs_D4 | OCs_D5 | OCs_D6 |
| CpG1 | 80.00 | 80.00 | 83.33 | 41.67 | 83.33 | 50.00 |
| CpG2 | 80.00 | 80.00 | 91.67 | 75.00 | 75.00 | 66.67 |
| CpG3 | 90.00 | 80.00 | 83.33 | 91.67 | 83.33 | 83.33 |
| CpG4 | 90.00 | 70.00 | 100.00 | 91.67 | 75.00 | 91.67 |
| CpG5 | 100.00 | 100.00 | 100.00 | 83.33 | 83.33 | 91.67 |
| CpG6 | 100.00 | 80.00 | 83.33 | 83.33 | 66.67 | 83.33 |
| CpG7 | 88.89 | 90.00 | 91.67 | 50.00 | 66.67 | 91.67 |
| CpG8 | 100.00 | 90.00 | 83.33 | 91.67 | 100.00 | 91.67 |
| CpG9 | 77.78 | 70.00 | 66.67 | 75.00 | 75.00 | 75.00 |
| CpG10 | 88.89 | 80.00 | 83.33 | 75.00 | 75.00 | 100.00 |
| CpG11 | 100.00 | 80.00 | 66.67 | 83.33 | 83.33 | 91.67 |
| CpG12 | 66.67 | 50.00 | 50.00 | 75.00 | 83.33 | 58.33 |
| CpG13 | 77.78 | 100.00 | 58.33 | 91.67 | 91.67 | 66.67 |
| CpG14 | 100.00 | 100.00 | 100.00 | 100.00 | 91.67 | 66.67 |
| CpG15 | 100.00 | 90.00 | 75.00 | 100.00 | 83.33 | 75.00 |
| CpG16 | 66.67 | 70.00 | 75.00 | 75.00 | 66.67 | 58.33 |
| CpG17 | 66.67 | 60.00 | 75.00 | 100.00 | 91.67 | 66.67 |
| CpG18 | 100.00 | 100.00 | 100.00 | 100.00 | 100.00 | 100.00 |
| CpG19 | 100.00 | 100.00 | 100.00 | 100.00 | 100.00 | 100.00 |

| **D4Z4** | MONOCYTES |  |  | OSTEOLASTS |  |  |
| --- | --- | --- | --- | --- | --- | --- |
| %Meth | MOs_D4 | MOs_D5 | MOs_D6 | OCs_D4 | OCs_D5 | OCs_D6 |
| CpG1 | 42.86 | 50.00 | 62.50 |  | 71.43 | 100.00 |
| CpG2 | 57.14 | 83.33 | 50.00 | 75.00 | 71.43 | 62.50 |
| CpG3 | 42.86 | 50.00 | 37.50 | 50.00 | 71.43 | 50.00 |
| CpG4 | 57.14 | 66.67 | 75.00 | 62.50 | 85.71 | 87.50 |
| CpG5 | 85.71 | 50.00 | 50.00 | 50.00 | 71.43 | 37.50 |
| CpG6 | 28.57 | 50.00 | 37.50 | 37.50 | 57.14 | 25.00 |
| CpG7 | 57.14 | 50.00 | 37.50 | 75.00 | 71.43 | 25.00 |
| CpG8 | 85.71 | 50.00 | 62.50 | 37.50 | 57.14 | 75.00 |
| CpG9 | 42.86 | 100.00 | 50.00 | 75.00 | 14.29 | 62.50 |
| CpG10 | 85.71 | 83.33 | 62.50 | 50.00 | 57.14 | 100.00 |
| CpG11 | 28.57 | 66.67 | 50.00 | 50.00 | 42.86 | 62.50 |
| CpG12 | 42.86 | 33.33 | 75.00 | 50.00 | 57.14 | 37.50 |
| CpG13 | 42.86 | 66.67 | 87.50 | 62.50 | 57.14 | 25.00 |
| CpG14 | 57.14 | 71.43 | 62.50 | 62.50 | 57.14 | 50.00 |
| CpG15 | 85.71 | 50.00 | 62.50 | 62.50 | 71.43 | 37.50 |
| CpG16 | 71.43 | 100.00 |  | 62.50 | 71.43 | 50.00 |
| CpG17 | 50.00 | 100.00 | 62.50 | 62.50 | 80.00 | 50.00 |
| CpG18 | 33.33 | 60.00 |  | 50.00 | 71.43 | 66.67 |
| CpG19 | 14.29 | 80.00 |  | 25.00 | 14.29 | 16.67 |

| **NBL2** | MONOCYTES |  |  | OSTEOLASTS |  |  |
| --- | --- | --- | --- | --- | --- | --- |
| %Meth | MOs_D4 | MOs_D5 | MOs_D6 | OCs_D4 | OCs_D5 | OCs_D6 |
| CpG1 | 100.00 | 87.50 | 100.00 | 100.00 | 100.00 | 87.50 |
| CpG2 | 85.71 | 100.00 | 100.00 | 87.50 | 100.00 | 87.50 |
| CpG3 | 85.71 | 100.00 | 100.00 | 100.00 | 87.50 | 100.00 |
| CpG4 | 100.00 | 87.50 | 85.71 | 87.50 | 100.00 | 100.00 |
| CpG5 | 100.00 | 87.50 | 100.00 | 75.00 | 100.00 | 87.50 |
| CpG6 | 71.43 | 87.50 | 100.00 | 75.00 | 62.50 | 87.50 |
| CpG7 | 100.00 | 100.00 | 100.00 | 100.00 | 100.00 | 100.00 |
| CpG8 | 100.00 | 100.00 | 85.71 | 75.00 | 100.00 | 100.00 |
| CpG9 | 100.00 | 62.50 | 100.00 | 62.50 | 62.50 | 100.00 |
| CpG10 | 42.86 | 50.00 | 57.14 | 37.50 | 62.50 | 62.50 |
| CpG11 | 42.86 | 62.50 | 71.43 | 25.00 | 75.00 | 75.00 |
| CpG12 | 28.57 | 25.00 | 42.86 | 50.00 | 28.57 | 12.50 |
| CpG13 | 100.00 | 87.50 | 71.43 | 100.00 | 100.00 | 87.50 |
| CpG14 | 85.71 | 62.50 | 57.14 | 62.50 | 100.00 | 75.00 |
| CpG15 | 42.86 | 87.50 | 85.71 | 100.00 | 100.00 | 100.00 |

**Summary Table time course: HYPOMETHYLATED GENES (Figure 2D)**

|  |  | | |  |  |  |  |  |  |  |  |  |  |  |  |  |  |  |  |  |  |  |
| --- | --- | --- | --- | --- | --- | --- | --- | --- | --- | --- | --- | --- | --- | --- | --- | --- | --- | --- | --- | --- | --- | --- |
|  | **ACP5_CPG1** |  | **ACP5_CPG2** |  | **ACP5_CPG3** |  | **CTSK** |  | **TM7SF4/DC STAMP** |  | **TM4SF19** |  |  |  |  |  |  |  | **IL7R** |  | **CD59** |  |
| **DAYS** | **%METH MEAN** | **SEM** | **%METH MEAN** | **SEM** | **%METH MEAN** | **SEM** | **%METH MEAN** | **SEM** | **%METH MEAN** | **SEM** | **%METH MEAN** | **SEM** | **%METH MEAN** | **SEM** | **%METH MEAN** | **SEM** | **%METH MEAN** | **SEM** | **%METH MEAN** | **SEM** | **%METH MEAN** | **SEM** |
| **0** | 80.83 | 2.14 | 85.50 | 7.82 | 70.33 | 2.34 | 92.17 | 0.54 | 80.50 | 0.96 | 81.46 | 1.36 | 87.73 | 5.59 | 84.21 | 2.92 | 63.26 | 2.24 | 34.64 | 4.06 | 61.75 | 6.18 |
| **1** | 80.67 | 0.52 | 68.17 | 8.01 | 52.50 | 5.79 | 90.00 | 1.06 | 56.67 | 6.17 | 65.62 | 1.63 | 78.72 | 6.41 | 64.93 | 8.16 | 42.26 | 9.28 | 36.86 | 1.53 | 38.13 | 8.80 |
| **2** | 75.00 | 10.84 | 32.50 | 7.40 | 27.67 | 4.56 | 85.83 | 1.58 | 37.67 | 6.38 | 56.11 | 5.07 | 70.47 | 12.07 | 54.40 | 12.70 | 28.39 | 12.47 | 31.72 | 3.36 | 31.39 | 10.00 |
| **4** | 60.17 | 5.27 | 16.67 | 3.14 | 13.83 | 2.48 | 57.17 | 4.21 | 25.50 | 4.58 | 39.75 | 1.94 | 53.94 | 3.78 | 37.51 | 3.80 | 19.71 | 2.08 | 27.06 | 3.12 | 16.45 | 3.54 |
| **5** | 58.67 | 8.19 | 17.50 | 5.05 | 18.17 | 5.00 | 42.67 | 3.79 | 24.83 | 2.52 | 35.38 | 2.92 | 45.99 | 8.93 | 30.36 | 8.66 | 16.53 | 7.02 | 26.35 | 3.17 | 18.90 | 2.39 |
| **6** | 52.40 | 13.63 | 10.67 | 4.13 | 9.00 | 4.98 | 38.50 | 5.53 | 24.00 | 5.87 | 27.19 | 2.17 | 34.97 | 4.53 | 22.88 | 2.14 | 10.57 | 2.99 | 24.35 | 4.47 | 16.41 | 8.93 |
| **7** | 47.17 | 4.07 | 8.67 | 1.86 | 6.50 | 2.81 | 31.67 | 6.15 | 19.67 | 5.57 | 28.87 | 3.46 | 47.63 | 8.21 | 30.59 | 9.02 | 14.61 | 4.97 | 22.52 | 3.27 | 9.91 | 3.91 |
| **8** | 46.20 | 7.01 | 7.33 | 2.66 | 5.50 | 2.26 | 26.50 | 5.21 | 24.50 | 3.32 | 24.34 | 3.22 | 40.55 | 5.07 | 23.51 | 2.93 | 10.41 | 4.45 | 22.40 | 2.27 | 9.96 | 4.62 |
| **9** | 43.17 | 5.53 | 11.00 | 3.46 | 7.83 | 3.31 | 20.33 | 4.00 | 15.50 | 2.67 | 22.05 | 1.20 | 33.73 | 4.67 | 19.54 | 4.15 | 8.46 | 3.26 | 23.28 | 2.23 | 9.38 | 2.80 |
| **11** | 40.33 | 4.63 | 7.83 | 1.60 | 4.83 | 1.17 | 18.33 | 3.53 | 13.50 | 1.84 | 17.09 | 0.61 | 30.15 | 3.79 | 14.84 | 3.30 | 5.34 | 2.44 | 22.18 | 2.21 | 6.24 | 2.32 |
| **12** | 38.50 | 5.51 | 9.00 | 1.83 | 7.00 | 0.82 | 20.75 | 4.66 | 8.50 | 3.62 | 18.92 | 3.06 | 28.82 | 5.50 | 13.32 | 4.70 | 6.39 | 3.20 | 22.70 | 2.82 | 10.50 | 3.92 |
| **13** | 42.00 | 7.29 | 5.67 | 2.80 | 3.17 | 2.40 | 15.33 | 3.85 | 9.17 | 2.23 | 22.91 | 4.85 | 33.89 | 11.20 | 13.77 | 3.70 | 5.64 | 2.73 | 24.14 | 2.53 | 6.69 | 2.54 |
| **14** | 42.50 | 1.73 | 6.75 | 1.26 | 5.25 | 2.50 | 15.50 | 3.75 | 7.75 | 1.03 | 25.80 | 5.49 | 26.27 | 5.89 | 18.15 | 12.79 | 8.76 | 7.27 | 26.08 | 2.35 | 7.80 | 3.26 |
| **16** | 36.50 | 4.64 | 6.83 | 2.64 | 3.83 | 1.17 | 10.33 | 1.71 | 10.33 | 1.50 | 17.31 | 2.54 | 27.05 | 5.66 | 16.23 | 8.96 | 7.85 | 7.10 | 23.09 | 2.15 | 4.60 | 2.03 |
| **19** | 36.00 | 4.38 | 4.33 | 1.51 | 4.33 | 3.14 | 7.67 | 1.87 | 10.33 | 2.32 | 12.74 | 1.05 | 24.02 | 4.75 | 9.65 | 3.13 | 2.69 | 2.09 | 24.97 | 3.07 | 4.93 | 4.43 |
| **21** | 33.60 | 5.98 | 4.60 | 1.95 | 1.50 | 1.00 | 6.50 | 2.62 | 5.00 | 1.52 | 11.59 | 6.12 | 21.60 | 9.45 | 13.15 | 9.72 | 3.61 | 2.18 | 16.17 | 4.93 | 4.75 | 6.21 |

**Summary Table time course: HYPERMETHYLATED GENES (Figure 2D)**

|  | **PPP1R16B** |  | **CD6** |  |  |  |  |  | **NR4A2** |  |  |  |  |  |  |  | **CD22** |  | **SNCG** |  | **CX3CR1** |  |
| --- | --- | --- | --- | --- | --- | --- | --- | --- | --- | --- | --- | --- | --- | --- | --- | --- | --- | --- | --- | --- | --- | --- |
| **DAYS** | **%METH MEAN** | **SEM** | **%METH MEAN** | **SEM** | **%METH MEAN** | **SEM** | **%METH MEAN** | **SEM** | **%METH MEAN** | **SEM** | **%METH MEAN** | **SEM** | **%METH MEAN** | **SEM** | **%METH MEAN** | **SEM** | **%METH MEAN** | **SEM** | **%METH MEAN** | **SEM** | **%METH MEAN** | **SEM** |
| **0** | 0.94 | 0.20 | 0.58 | 0.21 | 0.60 | 0.36 | 0.58 | 0.50 | 46.24 | 1.43 | 46.99 | 9.99 | 53.46 | 6.70 | 48.18 | 3.03 | 25.47 | 1.37 | 32.64 | 6.38 | 29.01 | 7.14 |
| **1** | 0.97 | 0.31 | 0.42 | 0.18 | 0.56 | 0.14 | 0.42 | 0.43 | 41.74 | 4.96 | 44.09 | 12.04 | 50.11 | 8.99 | 44.63 | 11.37 | 16.50 | 3.23 | 26.41 | 13.27 | 38.87 | 3.36 |
| **2** | 4.82 | 2.48 | 0.50 | 0.20 | 0.59 | 0.17 | 0.50 | 0.49 | 38.54 | 2.77 | 38.24 | 9.46 | 46.92 | 8.38 | 37.96 | 10.16 | 17.37 | 3.85 | 17.54 | 6.42 | 37.04 | 4.04 |
| **4** | 5.59 | 1.26 | 1.85 | 0.64 | 1.87 | 1.44 | 1.85 | 1.56 | 53.64 | 2.22 | 47.27 | 9.19 | 54.44 | 5.98 | 46.93 | 5.21 | 15.85 | 0.54 | 25.14 | 3.65 | 40.59 | 5.65 |
| **5** | 9.64 | 1.83 | 5.95 | 1.28 | 5.35 | 2.18 | 5.95 | 3.13 | 54.17 | 3.31 | 53.24 | 8.15 | 56.16 | 11.60 | 52.21 | 7.00 | 15.55 | 3.03 | 24.42 | 4.50 | 39.94 | 7.15 |
| **6** | 17.12 | 2.79 | 8.68 | 2.15 | 4.38 | 2.86 | 8.68 | 5.27 | 67.46 | 7.51 | 50.31 | 8.29 | 67.66 | 16.70 | 53.26 | 4.76 | 22.76 | 1.85 | 29.39 | 9.51 | 38.14 | 2.87 |
| **7** | 18.70 | 3.78 | 16.22 | 1.45 | 9.08 | 3.78 | 16.22 | 3.55 | 61.48 | 6.82 | 56.95 | 11.75 | 68.45 | 10.81 | 58.71 | 9.15 | 24.59 | 2.57 | 38.17 | 11.53 | 40.36 | 1.80 |
| **8** | 26.57 | 5.34 | 22.23 | 2.34 | 12.19 | 2.26 | 22.23 | 5.23 | 65.15 | 3.98 | 63.07 | 12.10 | 70.08 | 7.00 | 64.30 | 11.14 | 30.37 | 2.12 | 47.10 | 4.93 | 45.73 | 3.71 |
| **9** | 23.94 | 2.07 | 22.36 | 1.84 | 13.70 | 2.14 | 22.36 | 4.51 | 63.64 | 3.13 | 62.89 | 8.38 | 70.85 | 8.88 | 60.86 | 8.24 | 31.26 | 2.23 | 37.56 | 9.06 | 52.67 | 7.88 |
| **11** | 28.38 | 2.80 | 28.82 | 4.77 | 16.82 | 8.99 | 28.82 | 11.68 | 64.34 | 5.71 | 68.02 | 8.38 | 73.13 | 10.45 | 63.15 | 8.20 | 33.82 | 1.95 | 45.51 | 8.84 | 50.74 | 6.66 |
| **12** | 27.69 | 6.61 | 28.43 | 4.01 | 20.15 | 6.82 | 28.43 | 8.01 | 64.55 | 2.68 | 61.22 | 4.36 | 66.57 | 4.69 | 58.02 | 5.00 | 32.27 | 2.21 | 41.74 | 6.04 | 51.07 | 8.04 |
| **13** | 26.06 | 2.81 | 29.44 | 2.37 | 20.34 | 8.68 | 29.44 | 5.80 | 72.86 | 7.60 | 64.32 | 9.73 | 76.99 | 8.79 | 64.26 | 9.17 | 29.56 | 4.35 | 42.95 | 19.34 | 52.90 | 4.26 |
| **14** | 21.08 | 1.20 | 27.80 | 1.33 | 19.57 | 4.81 | 27.80 | 2.65 | 63.00 | 2.98 | 63.77 | 4.49 | 70.71 | 7.94 | 58.17 | 2.26 | 34.72 | 1.95 | 45.07 | 7.20 | 56.50 | 7.73 |
| **16** | 32.86 | 1.44 | 32.92 | 1.04 | 23.19 | 7.17 | 32.92 | 2.32 | 71.25 | 3.68 | 71.55 | 8.96 | 69.82 | 13.53 | 65.44 | 10.25 | 35.27 | 3.02 | 48.82 | 14.30 | 51.79 | 6.64 |
| **19** | 30.72 | 4.12 | 41.72 | 3.77 | 22.05 | 6.47 | 41.72 | 9.23 | 67.31 | 5.02 | 69.73 | 6.74 | 72.84 | 6.76 | 68.91 | 9.14 | 39.33 | 2.52 | 55.04 | 11.57 | 54.97 | 5.42 |
| **21** | 45.90 | 9.65 | 51.17 | 5.96 | 26.22 | 7.33 | 51.17 | 13.33 | 70.33 | 6.33 | 65.96 | 15.90 | 66.09 | 11.37 | 63.41 | 6.80 | 41.97 | 4.88 | 59.97 | 9.01 | 61.50 | 7.97 |

**Pyroseq. PU.1 siRNA (Fig 5D)**

|  | **siRNA control** |  | **siRNA PU.1** |  |  |  |
| --- | --- | --- | --- | --- | --- | --- |
| **ACP5** | **Mean** | **SEM** | **Mean** | **SEM** |  | **P-VALUE** |
| **Day 0** | 92.46 | 1.07 |  |  |  |  |
| **Day 1** | 72.34 | 2.22 | 76.87 | 1.10 |  | 0.069 |
| **Day 2** | 55.17 | 1.27 | 65.81 | 3.97 |  | 0.026 |
| **Day 4** | 31.90 | 4.52 | 58.33 | 2.50 |  | 0.006 |
| **Day 6** | 37.45 | 1.78 | 54.06 | 3.21 |  | 0.033 |
|  | **siRNA control** |  | **siRNA PU.1** |  |  |  |
| **CTSK** | **Mean** | **SEM** | **Mean** | **SEM** |  | **P-VALUE** |
| **Day 0** | 92.32 | 2.34 |  |  |  |  |
| **Day 1** | 87.14 | 2.19 | 85.80 | 2.39 |  | 0.21241 |
| **Day 2** | 60.98 | 4.23 | 82.96 | 1.43 |  | 0.03224 |
| **Day 4** | 59.11 | 5.37 | 82.33 | 0.30 |  | 0.01315 |
| **Day 6** | 45.33 | 5.55 | 77.18 | 1.52 |  | 0.01145 |
|  | **siRNA control** |  | **siRNA PU.1** |  |  |  |
| **CX3CR1** | **Mean** | **SEM** | **Mean** | **SEM** |  | **P-VALUE** |
| **Day 0** | 29.42 | 2.45 |  |  |  |  |
| **Day 1** | 37.40 | 0.90 | 33.43 | 2.62 |  | 0.20493 |
| **Day 2** | 40.98 | 0.28 | 29.50 | 0.89 |  | 0.00328 |
| **Day 4** | 42.18 | 0.03 | 30.38 | 0.35 |  | 0.00006 |
| **Day 6** | 42.36 | 5.29 | 34.89 | 0.35 |  | 0.17717 |
|  | **siRNA control** |  | **siRNA PU.1** |  |  |  |
| **NR4A2** | **Mean** | **SEM** | **Mean** | **SEM** |  | **P-VALUE** |
| **Day 0** | 28.46 | 1.24 |  |  |  |  |
| **Day 1** | 27.46 | 1.31 | 25.05 | 1.03 |  | 0.0925 |
| **Day 2** | 27.32 | 0.49 | 27.71 | 1.47 |  | 0.4123 |
| **Day 4** | 43.46 | 1.10 | 29.14 | 1.37 |  | 0.0009 |
| **Day 6** | 46.61 | 1.81 | 34.21 | 0.58 |  | 0.0053 |
|  | **siRNA control** |  | **siRNA PU.1** |  |  |  |
| **PLA2G4E** | **Mean** | **SEM** | **Mean** | **SEM** |  | **P-VALUE** |
| **Day 0** | 88.30 | 0.13 |  |  |  |  |
| **Day 1** | 70.97 | 9.22 | 70.42 | 1.93 |  | 0.4722 |
| **Day 2** | 68.82 | 3.03 | 64.05 | 2.42 |  | 0.1518 |
| **Day 4** | 57.25 | 10.39 | 53.64 | 5.59 |  | 0.3772 |
| **Day 6** | 36.12 | 8.06 | 36.65 | 0.78 |  | 0.4838 |
