# Supplementary material for: PU.1 target genes undergo Tet2-coupled demethylation and DNMT3b-mediated methylation in monocyte-to-osteoclast differentiation
Source: Genome Biol. 2013 Sep 12;14(9):R99. doi: 10.1186/gb-2013-14-9-r99 (PMC4054781; doi:10.1186/gb-2013-14-9-r99)
Supplement: Additional file 5 — Clusters of consecutive CpGs hypomethylated (-) or hypermethylated (+) in OC vs. MO. [file gb-2013-14-9-r99-S5.docx]

| **Additional file 5. Clusters of consecutive CpGs hypomethylated (-) or hypermethylated (+) in OC vs MO** | | | | | | |  |  |
| --- | --- | --- | --- | --- | --- | --- | --- | --- |
| NUMCLUSTER | FREQ | PERCENTAGE HYPO | PERCENTAGE HYPER | SIGN | NUM CPGS WINDOWS | GENOMIC LOC. aprox. | CHR | GENE |
| 1274 | 16 | 0 | 1 | '+' | 48 | 2321770-2322808 | 11 | TSPAN32, C11orf21 |
| 2318 | 10 | 0 | 1 | '+' | 30 | 24641021-24641852 | 14 | REC8 |
| 6204 | 10 | 1 | 0 | '-' | 30 | 33287809-33288599 | 6 | DAXX |
| 5397 | 9 | 1 | 0 | '-' | 27 | 196065106-196065688 | 3 | TM4SF19 |
| 3743 | 8 | 0 | 1 | '+' | 24 | 3178742-3180035 | 19 | S1PR4 |
| 3959 | 8 | 0 | 1 | '+' | 24 | 46318439-46319398 | 19 | RSPH6A, SYMPK |
| 4665 | 8 | 0 | 1 | '+' | 24 | 23066944-23067159 | 20 | CD93 |
| 5086 | 8 | 0 | 1 | '+' | 24 | 52528955-52529393 | 3 | STAB1 |
| 6521 | 8 | 0 | 1 | '+' | 24 | 1094737-1095720 | 7 | C7orf50, GPR146 |
| 858 | 7 | 1 | 0 | '-' | 21 | 3823844-3824687 | 10 | KLF6 |
| 1256 | 7 | 1 | 0 | '-' | 21 | 914849-915440 | 11 | CHID1 |
| 1431 | 7 | 0 | 1 | '+' | 21 | 60738971-60739183 | 11 | CD6 |
| 2023 | 7 | 0 | 1 | '+' | 21 | 111618925-111619455 | 12 | CUX2 |
| 3447 | 7 | 1 | 0 | '-' | 21 | 57917589-57918682 | 17 | TMEM49, MIR21 |
| 6116 | 7 | 1 | 0 | '-' | 21 | 30297174-30297565 | 6 | TRIM39 |
| 6570 | 7 | 0 | 1 | '+' | 21 | 2773782-2774654 | 7 | GNA12 |
| 972 | 6 | 1 | 0 | '-' | 18 | 63809073-63809170 | 10 | ARID5B |
| 1473 | 6 | 0 | 1 | '+' | 18 | 64981081-64981596 | 11 | SLC22A20 |
| 1760 | 6 | 1 | 0 | '-' | 18 | 7167584-7168545 | 12 | C1S |
| 2311 | 6 | 1 | 0 | '-' | 18 | 23623663-23624377 | 14 | SLC7A8 |
| 3389 | 6 | 0 | 1 | '+' | 18 | 46667587-46667812 | 17 | LOC404266 |
| 3599 | 6 | 0 | 1 | '+' | 18 | 79881468-79882042 | 17 | MAFG |
| 3899 | 6 | 0 | 1 | '+' | 18 | 35819775-35820181 | 19 | CD22 |
| 4908 | 6 | 0 | 1 | '+' | 18 | 45072491-45073165 | 22 | PRR5 |
| 4911 | 6 | 1 | 0 | '-' | 18 | 45608345-45608516 | 22 | C22orf9 |
| 5222 | 6 | 0 | 1 | '+' | 18 | 128779498-128779601 | 3 | GP9 |
| 5361 | 6 | 1 | 0 | '-' | 18 | 188664632-188664993 | 3 |  |
| 5649 | 6 | 0 | 1 | '+' | 18 | 174429263-174430141 | 4 |  |
| 5758 | 6 | 0 | 1 | '+' | 18 | 76115810-76116535 | 5 | F2RL1 |
| 5961 | 6 | 1 | 0 | '-' | 18 | 172305889-172306136 | 5 | ERGIC1 |
| 6372 | 6 | 0 | 1 | '+' | 18 | 111888446-111888683 | 6 | TRAF3IP2 |
| 31 | 5 | 0 | 1 | '+' | 15 | 3568210-3568245 | 1 | TP73 |
| 1006 | 5 | 0 | 1 | '+' | 15 | 73848458-73848915 | 10 | SPOCK2 |
| 1366 | 5 | 0 | 1 | '+' | 15 | 36422377-36422615 | 11 | PRR5L |
| 1381 | 5 | 0 | 1 | '+' | 15 | 46366813-46367100 | 11 | DGKZ |
| 1510 | 5 | 0 | 1 | '+' | 15 | 67176914-67177296 | 11 | TBC1D10C |
| 1526 | 5 | 0 | 1 | '+' | 15 | 68081207-68081686 | 11 | LRP5 |
| 2132 | 5 | 0 | 1 | '+' | 15 | 20805391-20805895 | 13 | GJB6 |
| 3263 | 5 | 0 | 1 | '+' | 15 | 19290353-19291120 | 17 | MFAP4 |
| 3351 | 5 | 1 | 0 | '-' | 15 | 40715222-40715281 | 17 | COASY |
| 3390 | 5 | 0 | 1 | '+' | 15 | 46669455-46669781 | 17 | HOXB5, LOC404266 |
| 3894 | 5 | 0 | 1 | '+' | 15 | 35630106-35630355 | 19 | FXYD1 |
| 3898 | 5 | 0 | 1 | '+' | 15 | 35786580-35787193 | 19 | MAG |
| 4412 | 5 | 0 | 1 | '+' | 15 | 157184416-157184978 | 2 | NR4A2 |
| 4634 | 5 | 0 | 1 | '+' | 15 | 825050-825415 | 20 | FAM110A |
| 5447 | 5 | 0 | 1 | '+' | 15 | 7657070-7657708 | 4 | SORCS2 |
| 5500 | 5 | 1 | 0 | '-' | 15 | 40517938-40518143 | 4 | RBM47 |
| 5511 | 5 | 0 | 1 | '+' | 15 | 54959419-54960034 | 4 |  |
| 5998 | 5 | 0 | 1 | '+' | 15 | 179220545-179221090 | 5 | LTC4S |
| 6106 | 5 | 1 | 0 | '-' | 15 | 30080393-30080968 | 6 | TRIM31 |
| 6395 | 5 | 1 | 0 | '-' | 15 | 134491143-134491531 | 6 | SGK1 |
| 6490 | 5 | 0 | 1 | '+' | 15 | 166876736-166877038 | 6 | RPS6KA2 |
| 6694 | 5 | 1 | 0 | '-' | 15 | 48129797-48129992 | 7 | UPP1 |
| 7196 | 5 | 1 | 0 | '-' | 15 | 141599141-141599436 | 8 | EIF2C2 |
| 7220 | 5 | 1 | 0 | '-' | 15 | 145018928-145019116 | 8 | PLEC1 |
| 122 | 4 | 0 | 1 | '+' | 12 | 17634543-17634716 | 1 | PADI4 |
| 167 | 4 | 0 | 1 | '+' | 12 | 24239820-24240069 | 1 | CNR2 |
| 342 | 4 | 0 | 1 | '+' | 12 | 54821853-54822503 | 1 | SSBP3 |
| 380 | 4 | 0 | 1 | '+' | 12 | 67772972-67773073 | 1 | IL12RB2 |
| 486 | 4 | 0 | 1 | '+' | 12 | 147012684-147013020 | 1 | BCL9 |
| 545 | 4 | 0 | 1 | '+' | 12 | 156211434-156211896 | 1 | BGLAP |
| 959 | 4 | 0 | 1 | '+' | 12 | 49892930-49893463 | 10 | WDFY4 |
| 1126 | 4 | 0 | 1 | '+' | 12 | 100174853-100175106 | 10 | PYROXD2 |
| 1139 | 4 | 0 | 1 | '+' | 12 | 102821427-102821684 | 10 | KAZALD1 |
| 1322 | 4 | 1 | 0 | '-' | 12 | 10476494-10476620 | 11 | AMPD3 |
| 1350 | 4 | 1 | 0 | '-' | 12 | 33744366-33744977 | 11 | CD59 |
| 1352 | 4 | 0 | 1 | '+' | 12 | 33913538-33914088 | 11 | LMO2 |
| 1477 | 4 | 1 | 0 | '-' | 12 | 65194933-65195039 | 11 |  |
| 1515 | 4 | 1 | 0 | '-' | 12 | 67205869-67206418 | 11 | PTPRCAP, CORO1B |
| 1551 | 4 | 1 | 0 | '-' | 12 | 71710614-71710982 | 11 | IL18BP |
| 1683 | 4 | 0 | 1 | '+' | 12 | 124767455-124767948 | 11 | ROBO4 |
| 2040 | 4 | 0 | 1 | '+' | 12 | 116996871-116997185 | 12 | MAP1LC3B2 |
| 2083 | 4 | 0 | 1 | '+' | 12 | 124864528-124865130 | 12 | NCOR2 |
| 2233 | 4 | 1 | 0 | '-' | 12 | 99135543-99135711 | 13 | STK24 |
| 2251 | 4 | 1 | 0 | '-' | 12 | 111522651-111522932 | 13 | C13orf29 |
| 2416 | 4 | 0 | 1 | '+' | 12 | 73706367-73706676 | 14 | PAPLN |
| 2527 | 4 | 1 | 0 | '-' | 12 | 104355829-104356172 | 14 |  |
| 3068 | 4 | 0 | 1 | '+' | 12 | 85116332-85116513 | 16 | KIAA0513 |
| 3106 | 4 | 1 | 0 | '-' | 12 | 88832476-88832572 | 16 | FAM38A |
| 3121 | 4 | 0 | 1 | '+' | 12 | 89042948-89043547 | 16 | CBFA2T3 |
| 3139 | 4 | 0 | 1 | '+' | 12 | 202588-203087 | 17 | RPH3AL |
| 3162 | 4 | 0 | 1 | '+' | 12 | 1548881-1549108 | 17 | SCARF1 |
| 3191 | 4 | 0 | 1 | '+' | 12 | 3819339-3819429 | 17 | P2RX1 |
| 3271 | 4 | 0 | 1 | '+' | 12 | 27045043-27045176 | 17 | RAB34 |
| 3304 | 4 | 0 | 1 | '+' | 12 | 33775917-33775961 | 17 | SLFN13 |
| 3438 | 4 | 0 | 1 | '+' | 12 | 56355299-56355431 | 17 | MPO |
| 3529 | 4 | 1 | 0 | '-' | 12 | 75446549-75446661 | 17 | SEPT9 |
| 3783 | 4 | 0 | 1 | '+' | 12 | 5074224-5074758 | 19 | KDM4B |
| 3871 | 4 | 1 | 0 | '-' | 12 | 18385244-18385930 | 19 | KIAA1683 |
| 3878 | 4 | 1 | 0 | '-' | 12 | 18771079-18771327 | 19 | KLHL26 |
| 3904 | 4 | 0 | 1 | '+' | 12 | 36247526-36248020 | 19 | HSPB6, C19orf55 |
| 4152 | 4 | 0 | 1 | '+' | 12 | 43398079-43398171 | 2 |  |
| 4317 | 4 | 0 | 1 | '+' | 12 | 108994311-108994528 | 2 | SULT1C4 |
| 4346 | 4 | 1 | 0 | '-' | 12 | 113875226-113875512 | 2 | IL1RN |
| 4614 | 4 | 0 | 1 | '+' | 12 | 241562085-241562650 | 2 |  |
| 4697 | 4 | 0 | 1 | '+' | 12 | 37433247-37433803 | 20 | PPP1R16B |
| 4712 | 4 | 0 | 1 | '+' | 12 | 45179157-45179413 | 20 | C20orf123 |
| 4840 | 4 | 0 | 1 | '+' | 12 | 24823509-24823554 | 22 | ADORA2A |
| 4902 | 4 | 0 | 1 | '+' | 12 | 44568337-44568775 | 22 | PARVG |
| 4917 | 4 | 0 | 1 | '+' | 12 | 46770063-46770440 | 22 | CELSR1 |
| 4951 | 4 | 0 | 1 | '+' | 12 | 9943828-9943937 | 3 | IL17RE |
| 4962 | 4 | 1 | 0 | '-' | 12 | 11610138-11610338 | 3 | VGLL4 |
| 5059 | 4 | 0 | 1 | '+' | 12 | 48471575-48471771 | 3 | PLXNB1 |
| 5271 | 4 | 1 | 0 | '-' | 12 | 149530133-149530341 | 3 | RNF13 |
| 5468 | 4 | 0 | 1 | '+' | 12 | 15964913-15965425 | 4 | FGFBP2 |
| 5838 | 4 | 0 | 1 | '+' | 12 | 134366914-134367394 | 5 | PITX1 |
| 6086 | 4 | 0 | 1 | '+' | 12 | 25042090-25042548 | 6 |  |
| 6115 | 4 | 1 | 0 | '-' | 12 | 30290408-30290603 | 6 | HCG18 |
| 6117 | 4 | 1 | 0 | '-' | 12 | 30301427-30301942 | 6 | TRIM39 |
| 6151 | 4 | 0 | 1 | '+' | 12 | 31690998-31691252 | 6 | C6orf25, LY6G6C |
| 6189 | 4 | 1 | 0 | '-' | 12 | 32917065-32917240 | 6 | HLA-DMA |
| 6197 | 4 | 1 | 0 | '-' | 12 | 33173307-33173581 | 6 | HSD17B8 |
| 6212 | 4 | 0 | 1 | '+' | 12 | 33400021-33400528 | 6 | SYNGAP1 |
| 6271 | 4 | 0 | 1 | '+' | 12 | 41168911-41168972 | 6 | TREML2 |
| 6350 | 4 | 0 | 1 | '+' | 12 | 106441441-106441506 | 6 |  |
| 6557 | 4 | 0 | 1 | '+' | 12 | 2653651-2654120 | 7 | IQCE |
| 6562 | 4 | 1 | 0 | '-' | 12 | 2679067-2679726 | 7 | TTYH3 |
| 6572 | 4 | 1 | 0 | '-' | 12 | 2802697-2802976 | 7 | GNA12 |
| 7201 | 4 | 0 | 1 | '+' | 12 | 142192468-142192718 | 8 | DENND3 |
| 68 | 3 | 1 | 0 | '-' | 9 | 9788767-9789174 | 1 | PIK3CD, CLSTN1 |
| 185 | 3 | 0 | 1 | '+' | 9 | 26097918-26098214 | 1 | MAN1C1 |
| 187 | 3 | 1 | 0 | '-' | 9 | 26347088-26347541 | 1 | EXTL1 |
| 203 | 3 | 0 | 1 | '+' | 9 | 27901805-27902555 | 1 | AHDC1 |
| 261 | 3 | 0 | 1 | '+' | 9 | 36948570-36948981 | 1 | CSF3R |
| 262 | 3 | 1 | 0 | '-' | 9 | 37941263-37941854 | 1 | ZC3H12A |
| 393 | 3 | 0 | 1 | '+' | 9 | 87617142-87617350 | 1 | LOC339524 |
| 429 | 3 | 0 | 1 | '+' | 9 | 110166563-110166664 | 1 | AMPD2 |
| 547 | 3 | 0 | 1 | '+' | 9 | 156357871-156358059 | 1 |  |
| 582 | 3 | 0 | 1 | '+' | 9 | 161068039-161068387 | 1 | KLHDC9 |
| 712 | 3 | 1 | 0 | '-' | 9 | 206643301-206643377 | 1 | IKBKE |
| 716 | 3 | 1 | 0 | '-' | 9 | 206729034-206729099 | 1 | RASSF5 |
| 834 | 3 | 0 | 1 | '+' | 9 | 243646235-243646395 | 1 | SDCCAG8 |
| 835 | 3 | 0 | 1 | '+' | 9 | 244213618-244214206 | 1 | ZNF238 |
| 974 | 3 | 1 | 0 | '-' | 9 | 64565750-64565772 | 10 | ADO |
| 1021 | 3 | 0 | 1 | '+' | 9 | 77164747-77165025 | 10 | C10orf41 |
| 1046 | 3 | 0 | 1 | '+' | 9 | 80853937-80854035 | 10 | ZMIZ1 |
| 1070 | 3 | 0 | 1 | '+' | 9 | 88717364-88717494 | 10 | SNCG, MMRN2 |
| 1101 | 3 | 0 | 1 | '+' | 9 | 94451736-94452554 | 10 | HHEX |
| 1105 | 3 | 1 | 0 | '-' | 9 | 95198200-95198374 | 10 | MYOF |
| 1169 | 3 | 0 | 1 | '+' | 9 | 105978651-105978956 | 10 | C10orf79, MIR609 |
| 1207 | 3 | 0 | 1 | '+' | 9 | 126222996-126223401 | 10 | LHPP |
| 1222 | 3 | 0 | 1 | '+' | 9 | 134211857-134211908 | 10 | PWWP2B |
| 1248 | 3 | 0 | 1 | '+' | 9 | 640446-641042 | 11 | DRD4, |
| 1259 | 3 | 1 | 0 | '-' | 9 | 1325718-1325852 | 11 | TOLLIP |
| 1275 | 3 | 0 | 1 | '+' | 9 | 2323801-2323938 | 11 | C11orf21, TSPAN32 |
| 1289 | 3 | 0 | 1 | '+' | 9 | 2908036-2908116 | 11 | CDKN1C |
| 1297 | 3 | 0 | 1 | '+' | 9 | 3177622-3178024 | 11 | OSBPL5 |
| 1316 | 3 | 0 | 1 | '+' | 9 | 8832203-8832283 | 11 | ST5 |
| 1393 | 3 | 0 | 1 | '+' | 9 | 47399813-47400330 | 11 | SPI1 |
| 1423 | 3 | 0 | 1 | '+' | 9 | 59823993-59824161 | 11 | MS4A3 |
| 1439 | 3 | 1 | 0 | '-' | 9 | 61449737-61449898 | 11 | DAGLA |
| 1470 | 3 | 1 | 0 | '-' | 9 | 64642144-64642558 | 11 | EHD1 |
| 1491 | 3 | 0 | 1 | '+' | 9 | 65816463-65816809 | 11 | GAL3ST3 |
| 1524 | 3 | 1 | 0 | '-' | 9 | 67811114-67811265 | 11 | TCIRG1 |
| 1545 | 3 | 0 | 1 | '+' | 9 | 69264259-69264657 | 11 |  |
| 1571 | 3 | 0 | 1 | '+' | 9 | 74178761-74178800 | 11 | KCNE3 |
| 1598 | 3 | 0 | 1 | '+' | 9 | 87908785-87908817 | 11 | RAB38 |
| 1682 | 3 | 0 | 1 | '+' | 9 | 124629580-124629925 | 11 | ESAM |
| 1695 | 3 | 0 | 1 | '+' | 9 | 128555317-128556152 | 11 |  |
| 1725 | 3 | 0 | 1 | '+' | 9 | 2030178-2030223 | 12 |  |
| 1745 | 3 | 0 | 1 | '+' | 9 | 6486639-6487080 | 12 | SCNN1A |
| 1821 | 3 | 0 | 1 | '+' | 9 | 29303160-29303219 | 12 |  |
| 1915 | 3 | 0 | 1 | '+' | 9 | 56414442-56414533 | 12 | IKZF4 |
| 1940 | 3 | 0 | 1 | '+' | 9 | 65671664-65672052 | 12 | MSRB3 |
| 2014 | 3 | 1 | 0 | '-' | 9 | 109221232-109221424 | 12 | SSH1 |
| 2022 | 3 | 0 | 1 | '+' | 9 | 111537057-111537195 | 12 | CUX2 |
| 2239 | 3 | 0 | 1 | '+' | 9 | 100310162-100310311 | 13 | CLYBL |
| 2249 | 3 | 1 | 0 | '-' | 9 | 111281324-111281479 | 13 | CARKD |
| 2451 | 3 | 1 | 0 | '-' | 9 | 88621424-88621579 | 14 |  |
| 2478 | 3 | 1 | 0 | '-' | 9 | 93419016-93419209 | 14 | ITPK1 |
| 2486 | 3 | 1 | 0 | '-' | 9 | 94423943-94424156 | 14 | ASB2 |
| 2491 | 3 | 1 | 0 | '-' | 9 | 94855034-94855344 | 14 | SERPINA1 |
| 2512 | 3 | 1 | 0 | '-' | 9 | 102394448-102394778 | 14 |  |
| 2531 | 3 | 1 | 0 | '-' | 9 | 105255251-105255361 | 14 | AKT1 |
| 2562 | 3 | 0 | 1 | '+' | 9 | 38988533-38988860 | 15 | C15orf53 |
| 2567 | 3 | 1 | 0 | '-' | 9 | 39871808-39871923 | 15 | THBS1 |
| 2576 | 3 | 0 | 1 | '+' | 9 | 41061384-41061527 | 15 | DNAJC17, C15orf62 |
| 2592 | 3 | 0 | 1 | '+' | 9 | 43531947-43532243 | 15 | TGM5 |
| 2600 | 3 | 1 | 0 | '-' | 9 | 45028083-45028270 | 15 | TRIM69 |
| 2697 | 3 | 0 | 1 | '+' | 9 | 70767183-70767649 | 15 |  |
| 2719 | 3 | 0 | 1 | '+' | 9 | 75470777-75471194 | 15 |  |
| 2772 | 3 | 0 | 1 | '+' | 9 | 91427184-91427363 | 15 | FES |
| 2799 | 3 | 0 | 1 | '+' | 9 | 101991512-101991828 | 15 | PCSK6 |
| 2812 | 3 | 1 | 0 | '-' | 9 | 1518592-1518751 | 16 | CLCN7 |
| 2833 | 3 | 1 | 0 | '-' | 9 | 3115223-3115552 | 16 | IL32 |
| 2852 | 3 | 1 | 0 | '-' | 9 | 5079023-5079174 | 16 | NAGPA |
| 2894 | 3 | 0 | 1 | '+' | 9 | 21171067-21171097 | 16 | TMEM159, DNAH3 |
| 2987 | 3 | 0 | 1 | '+' | 9 | 57576285-57576862 | 16 | GPR114 |
| 2996 | 3 | 0 | 1 | '+' | 9 | 66400404-66400569 | 16 | CDH5 |
| 3071 | 3 | 0 | 1 | '+' | 9 | 85343281-85343650 | 16 |  |
| 3088 | 3 | 0 | 1 | '+' | 9 | 86011615-86012305 | 16 |  |
| 3107 | 3 | 0 | 1 | '+' | 9 | 88844415-88844726 | 16 | FAM38A |
| 3120 | 3 | 0 | 1 | '+' | 9 | 89041793-89042185 | 16 | CBFA2T3 |
| 3122 | 3 | 0 | 1 | '+' | 9 | 89044523-89044882 | 16 | CBFA2T3 |
| 3125 | 3 | 1 | 0 | '-' | 9 | 89183643-89183728 | 16 | ACSF3 |
| 3128 | 3 | 1 | 0 | '-' | 9 | 89390609-89390968 | 16 | ANKRD11 |
| 3129 | 3 | 1 | 0 | '-' | 9 | 89408322-89408403 | 16 | ANKRD11 |
| 3203 | 3 | 0 | 1 | '+' | 9 | 5138259-5138645 | 17 | C17orf87 |
| 3215 | 3 | 0 | 1 | '+' | 9 | 7348379-7348438 | 17 | CHRNB1 |
| 3223 | 3 | 1 | 0 | '-' | 9 | 7792059-7792063 | 17 | CHD3 |
| 3300 | 3 | 1 | 0 | '-' | 9 | 32683418-32683481 | 17 | CCL13 |
| 3310 | 3 | 1 | 0 | '-' | 9 | 34207332-34207454 | 17 | CCL5 |
| 3321 | 3 | 0 | 1 | '+' | 9 | 37323484-37324234 | 17 | ARL5C, |
| 3349 | 3 | 0 | 1 | '+' | 9 | 40464625-40464935 | 17 |  |
| 3393 | 3 | 1 | 0 | '-' | 9 | 47287492-47287526 | 17 | ABI3, GNGT2 |
| 3411 | 3 | 0 | 1 | '+' | 9 | 48546258-48546620 | 17 | CHAD, ACSF2 |
| 3460 | 3 | 0 | 1 | '+' | 9 | 62009628-62009835 | 17 | CD79B |
| 3468 | 3 | 0 | 1 | '+' | 9 | 62774590-62775188 | 17 | LOC146880 |
| 3489 | 3 | 0 | 1 | '+' | 9 | 72443018-72443179 | 17 | GPRC5C |
| 3499 | 3 | 0 | 1 | '+' | 9 | 72732432-72732823 | 17 | RAB37 |
| 3507 | 3 | 0 | 1 | '+' | 9 | 73642486-73642607 | 17 | LOC100130933, RECQL5 |
| 3538 | 3 | 0 | 1 | '+' | 9 | 76128481-76128683 | 17 | TMC8, TMC6 |
| 3566 | 3 | 0 | 1 | '+' | 9 | 78735324-78735596 | 17 | RPTOR |
| 3570 | 3 | 0 | 1 | '+' | 9 | 78800767-78800806 | 17 | RPTOR |
| 3585 | 3 | 0 | 1 | '+' | 9 | 79297435-79297618 | 17 | TMEM105 |
| 3587 | 3 | 0 | 1 | '+' | 9 | 79316841-79317339 | 17 |  |
| 3614 | 3 | 1 | 0 | '-' | 9 | 80541549-80541737 | 17 | FOXK2 |
| 3647 | 3 | 0 | 1 | '+' | 9 | 20714332-20714496 | 18 | CABLES1 |
| 3692 | 3 | 0 | 1 | '+' | 9 | 827739-827843 | 19 | AZU1 |
| 3694 | 3 | 0 | 1 | '+' | 9 | 840873-841082 | 19 | PRTN3 |
| 3698 | 3 | 0 | 1 | '+' | 9 | 852114-852311 | 19 | ELANE |
| 3699 | 3 | 0 | 1 | '+' | 9 | 859330-859680 | 19 | CFD |
| 3707 | 3 | 0 | 1 | '+' | 9 | 947612-947765 | 19 | ARID3A |
| 3821 | 3 | 0 | 1 | '+' | 9 | 13215041-13215729 | 19 | LYL1, , TRMT1 |
| 3857 | 3 | 0 | 1 | '+' | 9 | 17357587-17357641 | 19 | NR2F6 |
| 3891 | 3 | 0 | 1 | '+' | 9 | 35531403-35531417 | 19 | HPN |
| 3993 | 3 | 0 | 1 | '+' | 9 | 51875946-51876470 | 19 | NKG7 |
| 4142 | 3 | 0 | 1 | '+' | 9 | 43188839-43188940 | 2 |  |
| 4280 | 3 | 1 | 0 | '-' | 9 | 97171137-97171243 | 2 | NEURL3 |
| 4478 | 3 | 0 | 1 | '+' | 9 | 203036147-203036235 | 2 |  |
| 4542 | 3 | 0 | 1 | '+' | 9 | 225265963-225266346 | 2 | FAM124B |
| 4587 | 3 | 0 | 1 | '+' | 9 | 238599734-238599858 | 2 | LRRFIP1 |
| 4691 | 3 | 1 | 0 | '-' | 9 | 36011999-36012016 | 20 | SRC |
| 4724 | 3 | 0 | 1 | '+' | 9 | 50108912-50109375 | 20 | NFATC2 |
| 4748 | 3 | 0 | 1 | '+' | 9 | 62492074-62492233 | 20 | C20orf135 |
| 4797 | 3 | 1 | 0 | '-' | 9 | 45575559-45575832 | 21 |  |
| 4803 | 3 | 0 | 1 | '+' | 9 | 45773569-45774294 | 21 | TRPM2 |
| 4805 | 3 | 1 | 0 | '-' | 9 | 46348306-46348691 | 21 | ITGB2 |
| 4821 | 3 | 0 | 1 | '+' | 9 | 19879320-19879787 | 22 | TXNRD2 |
| 4841 | 3 | 0 | 1 | '+' | 9 | 24890794-24890814 | 22 | C22orf45, UPB1 |
| 4846 | 3 | 0 | 1 | '+' | 9 | 27013978-27014116 | 22 | CRYBB1 |
| 4872 | 3 | 0 | 1 | '+' | 9 | 38092643-38092830 | 22 | TRIOBP |
| 4956 | 3 | 0 | 1 | '+' | 9 | 10334717-10334760 | 3 | GHRLOS, GHRL |
| 4980 | 3 | 1 | 0 | '-' | 9 | 15382723-15383022 | 3 | SH3BP5 |
| 5018 | 3 | 0 | 1 | '+' | 9 | 39323202-39323221 | 3 | CX3CR1 |
| 5113 | 3 | 0 | 1 | '+' | 9 | 69435510-69436045 | 3 | FRMD4B |
| 5180 | 3 | 1 | 0 | '-' | 9 | 114343673-114343825 | 3 | ZBTB20 |
| 5247 | 3 | 0 | 1 | '+' | 9 | 138048926-138049011 | 3 | TXNDC6 |
| 5286 | 3 | 0 | 1 | '+' | 9 | 155421970-155422145 | 3 | PLCH1 |
| 5346 | 3 | 1 | 0 | '-' | 9 | 187453721-187453973 | 3 | BCL6 |
| 5416 | 3 | 1 | 0 | '-' | 9 | 1304972-1305425 | 4 | MAEA |
| 5428 | 3 | 1 | 0 | '-' | 9 | 3387499-3387765 | 4 | RGS12 |
| 5544 | 3 | 0 | 1 | '+' | 9 | 84035837-84035953 | 4 | PLAC8 |
| 5568 | 3 | 1 | 0 | '-' | 9 | 99851060-99851211 | 4 | EIF4E |
| 5614 | 3 | 0 | 1 | '+' | 9 | 146856745-146857246 | 4 | ZNF827 |
| 5669 | 3 | 1 | 0 | '-' | 9 | 912712-912860 | 5 | TRIP13 |
| 5761 | 3 | 0 | 1 | '+' | 9 | 76248637-76248923 | 5 | CRHBP |
| 5868 | 3 | 0 | 1 | '+' | 9 | 139040546-139040849 | 5 | CXXC5 |
| 6007 | 3 | 1 | 0 | '-' | 9 | 209712-209809 | 6 |  |
| 6026 | 3 | 0 | 1 | '+' | 9 | 4890079-4890278 | 6 | CDYL |
| 6109 | 3 | 1 | 0 | '-' | 9 | 30124744-30124804 | 6 | TRIM10 |
| 6111 | 3 | 1 | 0 | '-' | 9 | 30131361-30131467 | 6 | TRIM15 |
| 6122 | 3 | 1 | 0 | '-' | 9 | 30558319-30558470 | 6 | ABCF1 |
| 6137 | 3 | 1 | 0 | '-' | 9 | 31540456-31540461 | 6 | LTA |
| 6139 | 3 | 1 | 0 | '-' | 9 | 31544931-31545321 | 6 | TNF |
| 6160 | 3 | 1 | 0 | '-' | 9 | 31913275-31913809 | 6 | CFB, C2 |
| 6183 | 3 | 1 | 0 | '-' | 9 | 32819964-32820355 | 6 | TAP1 |
| 6187 | 3 | 0 | 1 | '+' | 9 | 32905114-32905320 | 6 | HLA-DMB |
| 6195 | 3 | 1 | 0 | '-' | 9 | 33163689-33163824 | 6 | RXRB |
| 6201 | 3 | 1 | 0 | '-' | 9 | 33219335-33219410 | 6 | VPS52 |
| 6207 | 3 | 1 | 0 | '-' | 9 | 33386967-33387003 | 6 | CUTA, SYNGAP1 |
| 6209 | 3 | 0 | 1 | '+' | 9 | 33396295-33396407 | 6 | SYNGAP1 |
| 6249 | 3 | 0 | 1 | '+' | 9 | 36665554-36665620 | 6 |  |
| 6349 | 3 | 0 | 1 | '+' | 9 | 106434131-106434623 | 6 |  |
| 6353 | 3 | 1 | 0 | '-' | 9 | 106583161-106583218 | 6 |  |
| 6445 | 3 | 0 | 1 | '+' | 9 | 152128411-152128528 | 6 | ESR1 |
| 6487 | 3 | 0 | 1 | '+' | 9 | 163756931-163757048 | 6 |  |
| 6503 | 3 | 1 | 0 | '-' | 9 | 194719-194798 | 7 | FAM20C |
| 6506 | 3 | 1 | 0 | '-' | 9 | 213487-213848 | 7 | FAM20C |
| 6526 | 3 | 1 | 0 | '-' | 9 | 1560248-1560598 | 7 |  |
| 6542 | 3 | 0 | 1 | '+' | 9 | 2152625-2152821 | 7 | MAD1L1 |
| 6579 | 3 | 1 | 0 | '-' | 9 | 5270512-5270567 | 7 | WIPI2 |
| 6611 | 3 | 1 | 0 | '-' | 9 | 23286319-23286539 | 7 | GPNMB |
| 6623 | 3 | 0 | 1 | '+' | 9 | 27138396-27138751 | 7 |  |
| 6636 | 3 | 0 | 1 | '+' | 9 | 28220576-28220645 | 7 | JAZF1 |
| 6647 | 3 | 0 | 1 | '+' | 9 | 30951272-30951392 | 7 | AQP1 |
| 6761 | 3 | 0 | 1 | '+' | 9 | 92466386-92466842 | 7 | CDK6 |
| 6798 | 3 | 1 | 0 | '-' | 9 | 101768676-101768874 | 7 | CUX1 |
| 6867 | 3 | 0 | 1 | '+' | 9 | 140103671-140103737 | 7 | RAB19 |
| 6917 | 3 | 1 | 0 | '-' | 9 | 1725119-1725179 | 8 | CLN8 |
| 6967 | 3 | 0 | 1 | '+' | 9 | 22422657-22423091 | 8 | SORBS3 |
| 6989 | 3 | 0 | 1 | '+' | 9 | 27469186-27469338 | 8 | CLU |
| 7006 | 3 | 0 | 1 | '+' | 9 | 37556386-37557348 | 8 | ZNF703, |
| 7131 | 3 | 0 | 1 | '+' | 9 | 108510286-108510314 | 8 | ANGPT1 |
| 7198 | 3 | 0 | 1 | '+' | 9 | 141609338-141609470 | 8 | EIF2C2 |
| 7309 | 3 | 0 | 1 | '+' | 9 | 125796809-125797284 | 9 | GPR21, RABGAP1 |
| 7403 | 3 | 0 | 1 | '+' | 9 | 39948097-39948256 | X | BCOR |
| 5 | 2 | 0 | 1 | '+' | 6 | 1093940-1094080 | 1 |  |
| 13 | 2 | 0 | 1 | '+' | 6 | 1369793-1369934 | 1 | VWA1 |
| 28 | 2 | 1 | 0 | '-' | 6 | 3420815-3420848 | 1 | MEGF6 |
| 34 | 2 | 1 | 0 | '-' | 6 | 6341287-6341327 | 1 | ACOT7 |
| 50 | 2 | 0 | 1 | '+' | 6 | 9129646-9129648 | 1 | SLC2A5 |
| 53 | 2 | 0 | 1 | '+' | 6 | 9154250-9154254 | 1 |  |
| 56 | 2 | 1 | 0 | '-' | 6 | 9212514-9212754 | 1 | MIR34A |
| 58 | 2 | 1 | 0 | '-' | 6 | 9339380-9339683 | 1 |  |
| 82 | 2 | 0 | 1 | '+' | 6 | 11795897-11795937 | 1 | AGTRAP |
| 92 | 2 | 1 | 0 | '-' | 6 | 12238390-12238546 | 1 | TNFRSF1B |
| 94 | 2 | 0 | 1 | '+' | 6 | 12270775-12270887 | 1 |  |
| 114 | 2 | 1 | 0 | '-' | 6 | 16346317-16346434 | 1 | HSPB7 |
| 125 | 2 | 1 | 0 | '-' | 6 | 17951184-17951330 | 1 | ARHGEF10L |
| 139 | 2 | 1 | 0 | '-' | 6 | 19764480-19764686 | 1 | CAPZB |
| 153 | 2 | 1 | 0 | '-' | 6 | 22970072-22970132 | 1 | C1QC |
| 154 | 2 | 1 | 0 | '-' | 6 | 22972401-22972756 | 1 | C1QC |
| 156 | 2 | 1 | 0 | '-' | 6 | 22979495-22979739 | 1 | C1QB |
| 162 | 2 | 0 | 1 | '+' | 6 | 23855119-23855149 | 1 | E2F2 |
| 176 | 2 | 1 | 0 | '-' | 6 | 25254088-25254129 | 1 | RUNX3 |
| 213 | 2 | 0 | 1 | '+' | 6 | 28261552-28261706 | 1 | SMPDL3B |
| 229 | 2 | 0 | 1 | '+' | 6 | 32169568-32169868 | 1 | COL16A1 |
| 230 | 2 | 1 | 0 | '-' | 6 | 32264223-32264315 | 1 | SPOCD1 |
| 234 | 2 | 0 | 1 | '+' | 6 | 32802218-32802228 | 1 | MARCKSL1 |
| 250 | 2 | 0 | 1 | '+' | 6 | 36574795-36574854 | 1 |  |
| 254 | 2 | 1 | 0 | '-' | 6 | 36771781-36771793 | 1 | C1orf113 |
| 264 | 2 | 1 | 0 | '-' | 6 | 37947276-37947371 | 1 | ZC3H12A |
| 327 | 2 | 0 | 1 | '+' | 6 | 53019727-53019814 | 1 | ZCCHC11 |
| 338 | 2 | 0 | 1 | '+' | 6 | 54562040-54562121 | 1 | C1orf83 |
| 347 | 2 | 1 | 0 | '-' | 6 | 55316481-55316769 | 1 | DHCR24 |
| 354 | 2 | 0 | 1 | '+' | 6 | 59043576-59043873 | 1 | TACSTD2 |
| 364 | 2 | 0 | 1 | '+' | 6 | 61542211-61542329 | 1 | NFIA |
| 375 | 2 | 0 | 1 | '+' | 6 | 65731836-65731886 | 1 | DNAJC6 |
| 389 | 2 | 1 | 0 | '-' | 6 | 85085313-85085337 | 1 |  |
| 390 | 2 | 0 | 1 | '+' | 6 | 85665883-85665902 | 1 | SYDE2 |
| 394 | 2 | 0 | 1 | '+' | 6 | 87617972-87618313 | 1 | LOC339524 |
| 447 | 2 | 1 | 0 | '-' | 6 | 111743202-111743537 | 1 | DENND2D |
| 452 | 2 | 0 | 1 | '+' | 6 | 112057879-112058236 | 1 | ADORA3 |
| 458 | 2 | 1 | 0 | '-' | 6 | 113500329-113500384 | 1 | SLC16A1 |
| 466 | 2 | 1 | 0 | '-' | 6 | 116925613-116925753 | 1 | ATP1A1 |
| 483 | 2 | 0 | 1 | '+' | 6 | 145474469-145474701 | 1 | ANKRD34A |
| 506 | 2 | 0 | 1 | '+' | 6 | 151812435-151812710 | 1 | LOC100132111, C2CD4D |
| 512 | 2 | 0 | 1 | '+' | 6 | 153329781-153330068 | 1 | S100A9 |
| 516 | 2 | 1 | 0 | '-' | 6 | 153514264-153514376 | 1 | S100A5 |
| 517 | 2 | 1 | 0 | '-' | 6 | 153515502-153515539 | 1 | S100A5 |
| 527 | 2 | 0 | 1 | '+' | 6 | 154300117-154300241 | 1 | ATP8B2 |
| 531 | 2 | 1 | 0 | '-' | 6 | 154492947-154492952 | 1 | TDRD10 |
| 533 | 2 | 0 | 1 | '+' | 6 | 154943300-154943349 | 1 | SHC1 |
| 540 | 2 | 0 | 1 | '+' | 6 | 155290535-155290542 | 1 | C1orf104, RUSC1 |
| 554 | 2 | 0 | 1 | '+' | 6 | 156631235-156631242 | 1 |  |
| 563 | 2 | 1 | 0 | '-' | 6 | 159046391-159046451 | 1 | AIM2 |
| 567 | 2 | 0 | 1 | '+' | 6 | 159770136-159770209 | 1 |  |
| 590 | 2 | 1 | 0 | '-' | 6 | 161676469-161676581 | 1 | FCRLA |
| 598 | 2 | 1 | 0 | '-' | 6 | 167487295-167487633 | 1 | CD247 |
| 600 | 2 | 0 | 1 | '+' | 6 | 167598958-167598993 | 1 | RCSD1 |
| 645 | 2 | 1 | 0 | '-' | 6 | 182760143-182760430 | 1 | NPL |
| 647 | 2 | 1 | 0 | '-' | 6 | 183560913-183560923 | 1 | NCF2 |
| 662 | 2 | 0 | 1 | '+' | 6 | 200842890-200843156 | 1 | GPR25 |
| 683 | 2 | 1 | 0 | '-' | 6 | 203146223-203146346 | 1 | MYBPH |
| 691 | 2 | 0 | 1 | '+' | 6 | 203595145-203595283 | 1 | ATP2B4 |
| 725 | 2 | 0 | 1 | '+' | 6 | 206946166-206946187 | 1 | IL10 |
| 748 | 2 | 0 | 1 | '+' | 6 | 212588848-212589072 | 1 | TMEM206 |
| 759 | 2 | 0 | 1 | '+' | 6 | 221055790-221055964 | 1 | HLX |
| 766 | 2 | 1 | 0 | '-' | 6 | 223888869-223889141 | 1 | CAPN2 |
| 798 | 2 | 1 | 0 | '-' | 6 | 229480001-229480105 | 1 | C1orf96 |
| 847 | 2 | 0 | 1 | '+' | 6 | 247580074-247580106 | 1 | NLRP3 |
| 871 | 2 | 0 | 1 | '+' | 6 | 6020369-6020387 | 10 | IL15RA |
| 874 | 2 | 1 | 0 | '-' | 6 | 6183455-6183575 | 10 |  |
| 913 | 2 | 1 | 0 | '-' | 6 | 29923920-29924258 | 10 | SVIL |
| 923 | 2 | 0 | 1 | '+' | 6 | 31074367-31074432 | 10 |  |
| 944 | 2 | 0 | 1 | '+' | 6 | 45495971-45495981 | 10 | C10orf25, ZNF22 |
| 955 | 2 | 0 | 1 | '+' | 6 | 49731563-49731678 | 10 | ARHGAP22 |
| 958 | 2 | 1 | 0 | '-' | 6 | 49863421-49863809 | 10 |  |
| 996 | 2 | 0 | 1 | '+' | 6 | 73472315-73472344 | 10 | CDH23, C10orf105 |
| 1002 | 2 | 0 | 1 | '+' | 6 | 73534286-73534338 | 10 | C10orf54, CDH23 |
| 1015 | 2 | 0 | 1 | '+' | 6 | 75670435-75670653 | 10 | PLAU, C10orf55 |
| 1024 | 2 | 0 | 1 | '+' | 6 | 78647640-78647708 | 10 | KCNMA1 |
| 1054 | 2 | 0 | 1 | '+' | 6 | 81370414-81370678 | 10 | SFTPA1 |
| 1116 | 2 | 0 | 1 | '+' | 6 | 99259721-99259738 | 10 | MMS19, UBTD1 |
| 1124 | 2 | 1 | 0 | '-' | 6 | 99894013-99894087 | 10 | C10orf28 |
| 1138 | 2 | 0 | 1 | '+' | 6 | 102760724-102760784 | 10 | LZTS2 |
| 1181 | 2 | 1 | 0 | '-' | 6 | 112260509-112260867 | 10 | DUSP5 |
| 1215 | 2 | 0 | 1 | '+' | 6 | 129794994-129795003 | 10 | PTPRE |
| 1217 | 2 | 1 | 0 | '-' | 6 | 129845375-129845509 | 10 | PTPRE |
| 1219 | 2 | 1 | 0 | '-' | 6 | 134068039-134068305 | 10 | STK32C |
| 1223 | 2 | 0 | 1 | '+' | 6 | 134231487-134231517 | 10 |  |
| 1224 | 2 | 0 | 1 | '+' | 6 | 134258602-134258819 | 10 | C10orf91 |
| 1228 | 2 | 0 | 1 | '+' | 6 | 134362126-134362164 | 10 | INPP5A |
| 1229 | 2 | 0 | 1 | '+' | 6 | 134385407-134385698 | 10 | INPP5A |
| 1231 | 2 | 0 | 1 | '+' | 6 | 134404914-134404945 | 10 | INPP5A |
| 1236 | 2 | 0 | 1 | '+' | 6 | 134836370-134836426 | 10 |  |
| 1239 | 2 | 1 | 0 | '-' | 6 | 135118537-135118810 | 10 | TUBGCP2 |
| 1241 | 2 | 0 | 1 | '+' | 6 | 314341-314493 | 11 | IFITM1 |
| 1245 | 2 | 0 | 1 | '+' | 6 | 457278-457304 | 11 | PTDSS2 |
| 1258 | 2 | 1 | 0 | '-' | 6 | 977931-977965 | 11 | AP2A2 |
| 1262 | 2 | 0 | 1 | '+' | 6 | 1606344-1606664 | 11 | LOC338651, HCCA2, KRTAP5-1 |
| 1269 | 2 | 1 | 0 | '-' | 6 | 1886783-1886799 | 11 | LSP1 |
| 1270 | 2 | 0 | 1 | '+' | 6 | 1911511-1911547 | 11 | LSP1 |
| 1281 | 2 | 0 | 1 | '+' | 6 | 2677341-2677768 | 11 | KCNQ1, KCNQ1OT1 |
| 1290 | 2 | 0 | 1 | '+' | 6 | 2920765-2920789 | 11 | SLC22A18, SLC22A18AS |
| 1292 | 2 | 1 | 0 | '-' | 6 | 3071167-3071269 | 11 | CARS |
| 1296 | 2 | 0 | 1 | '+' | 6 | 3175625-3175636 | 11 | OSBPL5 |
| 1313 | 2 | 0 | 1 | '+' | 6 | 8228022-8228147 | 11 |  |
| 1345 | 2 | 0 | 1 | '+' | 6 | 19736333-19736648 | 11 | LOC100126784, NAV2 |
| 1351 | 2 | 0 | 1 | '+' | 6 | 33758413-33758505 | 11 | CD59 |
| 1406 | 2 | 1 | 0 | '-' | 6 | 57089821-57090226 | 11 | TNKS1BP1 |
| 1417 | 2 | 0 | 1 | '+' | 6 | 57529400-57529614 | 11 | CTNND1 |
| 1418 | 2 | 1 | 0 | '-' | 6 | 57559063-57559066 | 11 | CTNND1 |
| 1421 | 2 | 1 | 0 | '-' | 6 | 58981043-58981095 | 11 | MPEG1 |
| 1428 | 2 | 0 | 1 | '+' | 6 | 60623415-60623639 | 11 | GPR44 |
| 1434 | 2 | 0 | 1 | '+' | 6 | 60869760-60869910 | 11 | CD5 |
| 1451 | 2 | 0 | 1 | '+' | 6 | 63274030-63274230 | 11 | LGALS12 |
| 1455 | 2 | 0 | 1 | '+' | 6 | 63537378-63537459 | 11 | C11orf95 |
| 1469 | 2 | 1 | 0 | '-' | 6 | 64635728-64635774 | 11 | EHD1 |
| 1481 | 2 | 1 | 0 | '-' | 6 | 65318624-65318683 | 11 | LTBP3 |
| 1482 | 2 | 0 | 1 | '+' | 6 | 65325158-65325249 | 11 | LTBP3 |
| 1494 | 2 | 0 | 1 | '+' | 6 | 66084599-66084631 | 11 | CD248 |
| 1496 | 2 | 1 | 0 | '-' | 6 | 66104468-66104485 | 11 | RIN1 |
| 1502 | 2 | 0 | 1 | '+' | 6 | 66885276-66885281 | 11 | KDM2A |
| 1514 | 2 | 1 | 0 | '-' | 6 | 67203434-67203661 | 11 | PTPRCAP |
| 1529 | 2 | 0 | 1 | '+' | 6 | 68096138-68096165 | 11 | LRP5 |
| 1532 | 2 | 0 | 1 | '+' | 6 | 68147880-68148079 | 11 | LRP5 |
| 1537 | 2 | 0 | 1 | '+' | 6 | 68607622-68607737 | 11 | CPT1A |
| 1539 | 2 | 1 | 0 | '-' | 6 | 68972651-68972906 | 11 |  |
| 1543 | 2 | 1 | 0 | '-' | 6 | 69240844-69241075 | 11 |  |
| 1546 | 2 | 0 | 1 | '+' | 6 | 70211408-70211531 | 11 | PPFIA1 |
| 1548 | 2 | 0 | 1 | '+' | 6 | 70257059-70257149 | 11 | CTTN |
| 1553 | 2 | 0 | 1 | '+' | 6 | 71846733-71846788 | 11 | FOLR3 |
| 1554 | 2 | 1 | 0 | '-' | 6 | 71903119-71903227 | 11 | FOLR1 |
| 1564 | 2 | 1 | 0 | '-' | 6 | 72974126-72974155 | 11 | P2RY6 |
| 1577 | 2 | 1 | 0 | '-' | 6 | 75047111-75047180 | 11 | MIR326, ARRB1 |
| 1632 | 2 | 0 | 1 | '+' | 6 | 113953462-113953621 | 11 | ZBTB16 |
| 1638 | 2 | 0 | 1 | '+' | 6 | 117801332-117801492 | 11 | TMPRSS13 |
| 1647 | 2 | 0 | 1 | '+' | 6 | 118402280-118402342 | 11 | TMEM25 |
| 1709 | 2 | 1 | 0 | '-' | 6 | 322831-322888 | 12 | SLC6A12 |
| 1722 | 2 | 0 | 1 | '+' | 6 | 1949503-1949586 | 12 | CACNA2D4 |
| 1734 | 2 | 0 | 1 | '+' | 6 | 4919081-4919230 | 12 | KCNA6 |
| 1737 | 2 | 0 | 1 | '+' | 6 | 6234257-6234313 | 12 | VWF |
| 1746 | 2 | 0 | 1 | '+' | 6 | 6492948-6493003 | 12 | LTBR |
| 1751 | 2 | 0 | 1 | '+' | 6 | 6658164-6658625 | 12 | IFFO1 |
| 1832 | 2 | 1 | 0 | '-' | 6 | 46320890-46320944 | 12 | SFRS2IP |
| 1858 | 2 | 0 | 1 | '+' | 6 | 50445058-50445403 | 12 |  |
| 1887 | 2 | 0 | 1 | '+' | 6 | 53730072-53730163 | 12 | SP7 |
| 1891 | 2 | 0 | 1 | '+' | 6 | 54071165-54071168 | 12 | ATP5G2 |
| 1894 | 2 | 0 | 1 | '+' | 6 | 54689278-54689504 | 12 | NFE2 |
| 1896 | 2 | 0 | 1 | '+' | 6 | 54695016-54695036 | 12 | NFE2 |
| 1899 | 2 | 0 | 1 | '+' | 6 | 54779077-54779175 | 12 | ZNF385A |
| 1904 | 2 | 0 | 1 | '+' | 6 | 55378912-55379100 | 12 | KIAA0748 |
| 1925 | 2 | 0 | 1 | '+' | 6 | 57873704-57873732 | 12 | ARHGAP9 |
| 1926 | 2 | 1 | 0 | '-' | 6 | 57883279-57883315 | 12 | MARS |
| 1933 | 2 | 0 | 1 | '+' | 6 | 63025490-63025589 | 12 |  |
| 1976 | 2 | 0 | 1 | '+' | 6 | 93967580-93967711 | 12 | SOCS2 |
| 1986 | 2 | 0 | 1 | '+' | 6 | 96390960-96391123 | 12 | HAL |
| 1988 | 2 | 0 | 1 | '+' | 6 | 96587734-96587840 | 12 | ELK3 |
| 2031 | 2 | 0 | 1 | '+' | 6 | 113587513-113587581 | 12 | CCDC42B |
| 2052 | 2 | 1 | 0 | '-' | 6 | 121615078-121615201 | 12 | P2RX7 |
| 2056 | 2 | 1 | 0 | '-' | 6 | 121890311-121890328 | 12 | KDM2B |
| 2075 | 2 | 0 | 1 | '+' | 6 | 123518833-123518888 | 12 | PITPNM2 |
| 2079 | 2 | 0 | 1 | '+' | 6 | 123754071-123754328 | 12 | CDK2AP1 |
| 2087 | 2 | 0 | 1 | '+' | 6 | 124990897-124990942 | 12 | NCOR2 |
| 2089 | 2 | 0 | 1 | '+' | 6 | 125003064-125003379 | 12 | NCOR2 |
| 2097 | 2 | 0 | 1 | '+' | 6 | 125039219-125039343 | 12 |  |
| 2112 | 2 | 1 | 0 | '-' | 6 | 129329752-129329790 | 12 |  |
| 2122 | 2 | 0 | 1 | '+' | 6 | 133016214-133016265 | 12 |  |
| 2131 | 2 | 0 | 1 | '+' | 6 | 20768536-20768560 | 13 | GJB2 |
| 2164 | 2 | 0 | 1 | '+' | 6 | 33780283-33780307 | 13 | STARD13 |
| 2255 | 2 | 1 | 0 | '-' | 6 | 113425098-113425199 | 13 | ATP11A |
| 2257 | 2 | 0 | 1 | '+' | 6 | 113528375-113528751 | 13 | ATP11A |
| 2268 | 2 | 0 | 1 | '+' | 6 | 114301996-114302021 | 13 |  |
| 2272 | 2 | 0 | 1 | '+' | 6 | 114828264-114828455 | 13 | RASA3 |
| 2289 | 2 | 1 | 0 | '-' | 6 | 21467273-21467316 | 14 | SLC39A2 |
| 2301 | 2 | 0 | 1 | '+' | 6 | 23305941-23305957 | 14 | MMP14 |
| 2305 | 2 | 0 | 1 | '+' | 6 | 23351842-23351876 | 14 | REM2 |
| 2310 | 2 | 0 | 1 | '+' | 6 | 23589255-23589419 | 14 | CEBPE |
| 2324 | 2 | 0 | 1 | '+' | 6 | 24867472-24867954 | 14 | NYNRIN |
| 2329 | 2 | 1 | 0 | '-' | 6 | 31858067-31858202 | 14 | HEATR5A |
| 2334 | 2 | 1 | 0 | '-' | 6 | 35871954-35872393 | 14 | NFKBIA |
| 2337 | 2 | 0 | 1 | '+' | 6 | 39734964-39735211 | 14 | CTAGE5 |
| 2403 | 2 | 1 | 0 | '-' | 6 | 69522003-69522200 | 14 | DCAF5 |
| 2427 | 2 | 1 | 0 | '-' | 6 | 75747047-75747332 | 14 | FOS |
| 2428 | 2 | 1 | 0 | '-' | 6 | 75747961-75748252 | 14 | FOS |
| 2432 | 2 | 0 | 1 | '+' | 6 | 75894136-75894209 | 14 | JDP2 |
| 2446 | 2 | 0 | 1 | '+' | 6 | 81893864-81893935 | 14 |  |
| 2470 | 2 | 1 | 0 | '-' | 6 | 92994057-92994158 | 14 | RIN3 |
| 2485 | 2 | 1 | 0 | '-' | 6 | 94408121-94408232 | 14 | ASB2 |
| 2526 | 2 | 0 | 1 | '+' | 6 | 104171695-104171944 | 14 | XRCC3 |
| 2536 | 2 | 0 | 1 | '+' | 6 | 105779910-105779952 | 14 | PACS2 |
| 2544 | 2 | 1 | 0 | '-' | 6 | 22954791-22954817 | 15 | CYFIP1 |
| 2550 | 2 | 0 | 1 | '+' | 6 | 29395932-29396200 | 15 | APBA2 |
| 2558 | 2 | 1 | 0 | '-' | 6 | 34628731-34628769 | 15 | SLC12A6 |
| 2571 | 2 | 0 | 1 | '+' | 6 | 40642998-40643142 | 15 | PHGR1 |
| 2594 | 2 | 0 | 1 | '+' | 6 | 43809689-43809865 | 15 | MAP1A |
| 2631 | 2 | 1 | 0 | '-' | 6 | 58430539-58430682 | 15 | AQP9 |
| 2645 | 2 | 1 | 0 | '-' | 6 | 60688622-60688948 | 15 | ANXA2 |
| 2678 | 2 | 1 | 0 | '-' | 6 | 68564546-68564635 | 15 |  |
| 2690 | 2 | 0 | 1 | '+' | 6 | 70387217-70387268 | 15 | TLE3 |
| 2696 | 2 | 0 | 1 | '+' | 6 | 70740429-70740451 | 15 |  |
| 2721 | 2 | 0 | 1 | '+' | 6 | 75639339-75639433 | 15 | NEIL1 |
| 2726 | 2 | 0 | 1 | '+' | 6 | 76633627-76633817 | 15 | ISL2 |
| 2727 | 2 | 0 | 1 | '+' | 6 | 76635638-76635953 | 15 |  |
| 2752 | 2 | 1 | 0 | '-' | 6 | 86222553-86222754 | 15 | AKAP13 |
| 2760 | 2 | 0 | 1 | '+' | 6 | 90293274-90293408 | 15 | MESP1 |
| 2765 | 2 | 1 | 0 | '-' | 6 | 90727560-90727570 | 15 | SEMA4B |
| 2773 | 2 | 0 | 1 | '+' | 6 | 91427965-91428056 | 15 | FES |
| 2783 | 2 | 0 | 1 | '+' | 6 | 94840953-94841112 | 15 | MCTP2 |
| 2792 | 2 | 0 | 1 | '+' | 6 | 100890963-100890996 | 15 | FLJ42289 |
| 2797 | 2 | 0 | 1 | '+' | 6 | 101777761-101777800 | 15 | CHSY1 |
| 2801 | 2 | 0 | 1 | '+' | 6 | 126397-126855 | 16 | MPG |
| 2803 | 2 | 0 | 1 | '+' | 6 | 374988-375327 | 16 | AXIN1 |
| 2807 | 2 | 1 | 0 | '-' | 6 | 1014818-1014935 | 16 | LMF1 |
| 2818 | 2 | 1 | 0 | '-' | 6 | 1742245-1742281 | 16 | HN1L |
| 2822 | 2 | 0 | 1 | '+' | 6 | 2198075-2198080 | 16 | RAB26 |
| 2824 | 2 | 1 | 0 | '-' | 6 | 2569920-2569925 | 16 | AMDHD2, ATP6V0C |
| 2829 | 2 | 0 | 1 | '+' | 6 | 3068467-3068475 | 16 | CLDN6 |
| 2880 | 2 | 1 | 0 | '-' | 6 | 15596369-15596423 | 16 | C16orf45 |
| 2899 | 2 | 1 | 0 | '-' | 6 | 21665138-21665238 | 16 | IGSF6, METTL9 |
| 2926 | 2 | 0 | 1 | '+' | 6 | 29165553-29165632 | 16 |  |
| 2929 | 2 | 0 | 1 | '+' | 6 | 29690271-29690524 | 16 | QPRT |
| 2932 | 2 | 1 | 0 | '-' | 6 | 30198505-30198509 | 16 | CORO1A, LOC606724 |
| 2955 | 2 | 0 | 1 | '+' | 6 | 46782760-46783018 | 16 | MYLK3 |
| 2968 | 2 | 0 | 1 | '+' | 6 | 50347849-50347895 | 16 | ADCY7 |
| 2999 | 2 | 0 | 1 | '+' | 6 | 66613053-66613096 | 16 | CMTM2 |
| 3006 | 2 | 0 | 1 | '+' | 6 | 67196462-67196699 | 16 | HSF4, FBXL8 |
| 3058 | 2 | 1 | 0 | '-' | 6 | 84628969-84629008 | 16 | COTL1 |
| 3063 | 2 | 0 | 1 | '+' | 6 | 84860871-84860918 | 16 | CRISPLD2 |
| 3080 | 2 | 0 | 1 | '+' | 6 | 85881502-85881624 | 16 |  |
| 3083 | 2 | 0 | 1 | '+' | 6 | 85964073-85964200 | 16 |  |
| 3089 | 2 | 0 | 1 | '+' | 6 | 86016317-86016387 | 16 |  |
| 3090 | 2 | 0 | 1 | '+' | 6 | 86018680-86018936 | 16 |  |
| 3118 | 2 | 0 | 1 | '+' | 6 | 89033785-89033895 | 16 | CBFA2T3 |
| 3126 | 2 | 0 | 1 | '+' | 6 | 89185916-89186403 | 16 | ACSF3 |
| 3132 | 2 | 0 | 1 | '+' | 6 | 89778301-89778660 | 16 | C16orf7 |
| 3137 | 2 | 0 | 1 | '+' | 6 | 179611-179838 | 17 | RPH3AL |
| 3142 | 2 | 0 | 1 | '+' | 6 | 398066-398090 | 17 |  |
| 3160 | 2 | 1 | 0 | '-' | 6 | 1510000-1510041 | 17 | SLC43A2 |
| 3165 | 2 | 1 | 0 | '-' | 6 | 1639375-1639402 | 17 | WDR81 |
| 3171 | 2 | 0 | 1 | '+' | 6 | 1959596-1959620 | 17 | HIC1 |
| 3172 | 2 | 0 | 1 | '+' | 6 | 1962132-1962236 | 17 | HIC1 |
| 3175 | 2 | 0 | 1 | '+' | 6 | 2300100-2300514 | 17 | MNT |
| 3182 | 2 | 0 | 1 | '+' | 6 | 2839005-2839082 | 17 | RAP1GAP2 |
| 3183 | 2 | 0 | 1 | '+' | 6 | 2907895-2908023 | 17 | RAP1GAP2 |
| 3192 | 2 | 0 | 1 | '+' | 6 | 3820044-3820150 | 17 | P2RX1 |
| 3193 | 2 | 0 | 1 | '+' | 6 | 3820796-3820910 | 17 | P2RX1 |
| 3198 | 2 | 0 | 1 | '+' | 6 | 4648566-4648580 | 17 | ZMYND15 |
| 3218 | 2 | 1 | 0 | '-' | 6 | 7477972-7478131 | 17 | EIF4A1, SNORA48 |
| 3225 | 2 | 0 | 1 | '+' | 6 | 7959907-7960092 | 17 |  |
| 3227 | 2 | 1 | 0 | '-' | 6 | 8384595-8384607 | 17 | MYH10 |
| 3257 | 2 | 0 | 1 | '+' | 6 | 17696044-17696392 | 17 | RAI1 |
| 3283 | 2 | 0 | 1 | '+' | 6 | 27918316-27918338 | 17 |  |
| 3305 | 2 | 0 | 1 | '+' | 6 | 33776554-33776683 | 17 | SLFN13 |
| 3307 | 2 | 0 | 1 | '+' | 6 | 33825172-33825300 | 17 | SLFN12L |
| 3325 | 2 | 0 | 1 | '+' | 6 | 37894413-37894636 | 17 | GRB7 |
| 3333 | 2 | 0 | 1 | '+' | 6 | 38501519-38501677 | 17 | RARA |
| 3339 | 2 | 1 | 0 | '-' | 6 | 39624076-39624088 | 17 | KRT32 |
| 3350 | 2 | 1 | 0 | '-' | 6 | 40489513-40489569 | 17 | STAT3 |
| 3357 | 2 | 0 | 1 | '+' | 6 | 41857603-41857647 | 17 | DUSP3, C17orf105 |
| 3372 | 2 | 0 | 1 | '+' | 6 | 43325657-43325791 | 17 | LOC100133991 |
| 3388 | 2 | 0 | 1 | '+' | 6 | 46666926-46666958 | 17 | LOC404266 |
| 3440 | 2 | 0 | 1 | '+' | 6 | 56357994-56358318 | 17 | MPO |
| 3443 | 2 | 1 | 0 | '-' | 6 | 56605391-56605468 | 17 | SEPT4 |
| 3462 | 2 | 0 | 1 | '+' | 6 | 62075103-62075324 | 17 | C17orf72 |
| 3464 | 2 | 1 | 0 | '-' | 6 | 62208374-62208434 | 17 | ERN1 |
| 3467 | 2 | 0 | 1 | '+' | 6 | 62773704-62774079 | 17 | LOC146880 |
| 3472 | 2 | 1 | 0 | '-' | 6 | 63053929-63053996 | 17 | GNA13 |
| 3486 | 2 | 0 | 1 | '+' | 6 | 71948506-71948613 | 17 |  |
| 3491 | 2 | 0 | 1 | '+' | 6 | 72462559-72462984 | 17 | CD300A |
| 3498 | 2 | 0 | 1 | '+' | 6 | 72709026-72709096 | 17 | CD300LF, RAB37 |
| 3516 | 2 | 1 | 0 | '-' | 6 | 74494213-74494286 | 17 | RHBDF2 |
| 3525 | 2 | 0 | 1 | '+' | 6 | 75385278-75385432 | 17 | SEPT9 |
| 3531 | 2 | 0 | 1 | '+' | 6 | 75471193-75471246 | 17 | SEPT9 |
| 3537 | 2 | 0 | 1 | '+' | 6 | 76126301-76126702 | 17 | TMC8, TMC6 |
| 3552 | 2 | 1 | 0 | '-' | 6 | 76899257-76899379 | 17 | TIMP2 |
| 3553 | 2 | 0 | 1 | '+' | 6 | 77752742-77753199 | 17 | CBX2 |
| 3554 | 2 | 0 | 1 | '+' | 6 | 77755547-77755965 | 17 | CBX2 |
| 3567 | 2 | 0 | 1 | '+' | 6 | 78748077-78748494 | 17 | RPTOR |
| 3575 | 2 | 0 | 1 | '+' | 6 | 79004947-79005047 | 17 | FLJ90757 |
| 3578 | 2 | 1 | 0 | '-' | 6 | 79031091-79031162 | 17 | BAIAP2 |
| 3580 | 2 | 1 | 0 | '-' | 6 | 79107197-79107259 | 17 | AATK, MIR1250 |
| 3581 | 2 | 1 | 0 | '-' | 6 | 79127372-79127459 | 17 | AATK |
| 3588 | 2 | 0 | 1 | '+' | 6 | 79360405-79360456 | 17 |  |
| 3590 | 2 | 0 | 1 | '+' | 6 | 79366851-79366853 | 17 |  |
| 3600 | 2 | 0 | 1 | '+' | 6 | 79924542-79924772 | 17 |  |
| 3615 | 2 | 0 | 1 | '+' | 6 | 80581701-80581805 | 17 | WDR45L |
| 3619 | 2 | 1 | 0 | '-' | 6 | 80829261-80829309 | 17 | TBCD |
| 3625 | 2 | 0 | 1 | '+' | 6 | 80944090-80944193 | 17 | B3GNTL1 |
| 3626 | 2 | 1 | 0 | '-' | 6 | 81014009-81014091 | 17 |  |
| 3629 | 2 | 1 | 0 | '-' | 6 | 81040724-81040906 | 17 | METRNL |
| 3631 | 2 | 1 | 0 | '-' | 6 | 81047784-81047941 | 17 | METRNL |
| 3637 | 2 | 1 | 0 | '-' | 6 | 10524509-10524910 | 18 | NAPG |
| 3645 | 2 | 1 | 0 | '-' | 6 | 13375474-13375540 | 18 | C18orf1 |
| 3646 | 2 | 0 | 1 | '+' | 6 | 13641735-13641872 | 18 | C18orf1 |
| 3648 | 2 | 0 | 1 | '+' | 6 | 20716991-20717411 | 18 | CABLES1 |
| 3653 | 2 | 1 | 0 | '-' | 6 | 21452788-21452819 | 18 | LAMA3 |
| 3658 | 2 | 1 | 0 | '-' | 6 | 32289474-32289550 | 18 | DTNA |
| 3679 | 2 | 1 | 0 | '-' | 6 | 74824154-74824280 | 18 | MBP |
| 3714 | 2 | 1 | 0 | '-' | 6 | 1140909-1140926 | 19 | SBNO2 |
| 3717 | 2 | 1 | 0 | '-' | 6 | 1168987-1169138 | 19 | SBNO2 |
| 3732 | 2 | 0 | 1 | '+' | 6 | 2446487-2446633 | 19 | LMNB2 |
| 3735 | 2 | 1 | 0 | '-' | 6 | 2546844-2546938 | 19 | GNG7 |
| 3744 | 2 | 1 | 0 | '-' | 6 | 3201528-3201535 | 19 | NCLN |
| 3751 | 2 | 1 | 0 | '-' | 6 | 3464991-3465071 | 19 |  |
| 3760 | 2 | 0 | 1 | '+' | 6 | 3688176-3688477 | 19 | PIP5K1C |
| 3770 | 2 | 0 | 1 | '+' | 6 | 4302448-4302579 | 19 | TMIGD2 |
| 3772 | 2 | 1 | 0 | '-' | 6 | 4391454-4391533 | 19 | SH3GL1 |
| 3789 | 2 | 1 | 0 | '-' | 6 | 6721016-6721027 | 19 | C3 |
| 3810 | 2 | 0 | 1 | '+' | 6 | 11305913-11305924 | 19 | KANK2 |
| 3816 | 2 | 1 | 0 | '-' | 6 | 12776725-12777225 | 19 | MAN2B1, MORG1 |
| 3819 | 2 | 0 | 1 | '+' | 6 | 13211225-13211261 | 19 | LYL1 |
| 3831 | 2 | 0 | 1 | '+' | 6 | 14089284-14089630 | 19 | RFX1 |
| 3833 | 2 | 0 | 1 | '+' | 6 | 14260591-14260651 | 19 | LPHN1 |
| 3841 | 2 | 0 | 1 | '+' | 6 | 14693742-14693814 | 19 | CLEC17A |
| 3844 | 2 | 1 | 0 | '-' | 6 | 15375283-15375319 | 19 | BRD4 |
| 3845 | 2 | 0 | 1 | '+' | 6 | 15575612-15575652 | 19 | RASAL3 |
| 3849 | 2 | 0 | 1 | '+' | 6 | 16254489-16254516 | 19 | HSH2D |
| 3896 | 2 | 1 | 0 | '-' | 6 | 35719621-35719778 | 19 | FAM187B |
| 3897 | 2 | 1 | 0 | '-' | 6 | 35782015-35782257 | 19 | MAG |
| 3901 | 2 | 1 | 0 | '-' | 6 | 35940425-35940461 | 19 | FFAR2 |
| 3905 | 2 | 0 | 1 | '+' | 6 | 36400425-36400437 | 19 | TYROBP |
| 3918 | 2 | 1 | 0 | '-' | 6 | 40169839-40169912 | 19 | LOC400696 |
| 3942 | 2 | 1 | 0 | '-' | 6 | 44285594-44285940 | 19 | KCNN4 |
| 3947 | 2 | 1 | 0 | '-' | 6 | 45417793-45417814 | 19 | APOC1 |
| 3948 | 2 | 1 | 0 | '-' | 6 | 45445437-45445693 | 19 | APOC4 |
| 3949 | 2 | 1 | 0 | '-' | 6 | 45449006-45449099 | 19 | APOC2 |
| 3955 | 2 | 0 | 1 | '+' | 6 | 46001735-46001746 | 19 | FLJ40125, RTN2 |
| 3984 | 2 | 0 | 1 | '+' | 6 | 50015780-50015975 | 19 | FCGRT |
| 3986 | 2 | 1 | 0 | '-' | 6 | 50117794-50117948 | 19 | PRR12 |
| 3991 | 2 | 0 | 1 | '+' | 6 | 51628115-51628121 | 19 | SIGLEC9 |
| 4010 | 2 | 1 | 0 | '-' | 6 | 54761090-54761178 | 19 | LILRB5 |
| 4020 | 2 | 0 | 1 | '+' | 6 | 7171963-7171998 | 2 | RNF144A |
| 4033 | 2 | 0 | 1 | '+' | 6 | 8818142-8818257 | 2 |  |
| 4044 | 2 | 1 | 0 | '-' | 6 | 10302454-10302462 | 2 | C2orf48 |
| 4049 | 2 | 0 | 1 | '+' | 6 | 11885266-11885560 | 2 | LPIN1 |
| 4072 | 2 | 0 | 1 | '+' | 6 | 25499619-25499764 | 2 | DNMT3A |
| 4096 | 2 | 1 | 0 | '-' | 6 | 29343915-29344035 | 2 | CLIP4 |
| 4144 | 2 | 0 | 1 | '+' | 6 | 43202474-43202481 | 2 |  |
| 4177 | 2 | 1 | 0 | '-' | 6 | 46768030-46768111 | 2 |  |
| 4243 | 2 | 0 | 1 | '+' | 6 | 71132518-71132993 | 2 | VAX2 |
| 4247 | 2 | 1 | 0 | '-' | 6 | 73118624-73119097 | 2 | SPR |
| 4250 | 2 | 0 | 1 | '+' | 6 | 74208602-74208655 | 2 |  |
| 4269 | 2 | 0 | 1 | '+' | 6 | 85921036-85921252 | 2 | GNLY |
| 4273 | 2 | 0 | 1 | '+' | 6 | 86155886-86155952 | 2 |  |
| 4282 | 2 | 0 | 1 | '+' | 6 | 97359879-97359926 | 2 | FER1L5 |
| 4294 | 2 | 1 | 0 | '-' | 6 | 101922427-101922922 | 2 | RNF149 |
| 4299 | 2 | 0 | 1 | '+' | 6 | 102313069-102313242 | 2 | MAP4K4 |
| 4309 | 2 | 0 | 1 | '+' | 6 | 102972758-102972792 | 2 |  |
| 4315 | 2 | 0 | 1 | '+' | 6 | 106776775-106777038 | 2 | UXS1 |
| 4352 | 2 | 0 | 1 | '+' | 6 | 118982661-118982679 | 2 |  |
| 4354 | 2 | 0 | 1 | '+' | 6 | 119699682-119699789 | 2 | MARCO |
| 4361 | 2 | 0 | 1 | '+' | 6 | 127414883-127415063 | 2 | GYPC |
| 4372 | 2 | 1 | 0 | '-' | 6 | 129248896-129249087 | 2 |  |
| 4411 | 2 | 0 | 1 | '+' | 6 | 157183291-157183755 | 2 | NR4A2 |
| 4430 | 2 | 0 | 1 | '+' | 6 | 169967580-169967583 | 2 |  |
| 4435 | 2 | 0 | 1 | '+' | 6 | 172962700-172963125 | 2 |  |
| 4439 | 2 | 0 | 1 | '+' | 6 | 173293627-173294093 | 2 | ITGA6 |
| 4444 | 2 | 0 | 1 | '+' | 6 | 173940203-173940277 | 2 | ZAK |
| 4500 | 2 | 1 | 0 | '-' | 6 | 216708370-216708378 | 2 |  |
| 4553 | 2 | 0 | 1 | '+' | 6 | 231731972-231732249 | 2 | ITM2C |
| 4554 | 2 | 0 | 1 | '+' | 6 | 231735037-231735434 | 2 | ITM2C |
| 4583 | 2 | 1 | 0 | '-' | 6 | 238406432-238406478 | 2 | MLPH |
| 4588 | 2 | 1 | 0 | '-' | 6 | 238647857-238647913 | 2 | LRRFIP1 |
| 4608 | 2 | 0 | 1 | '+' | 6 | 240499800-240499816 | 2 |  |
| 4612 | 2 | 0 | 1 | '+' | 6 | 240884831-240884925 | 2 |  |
| 4615 | 2 | 1 | 0 | '-' | 6 | 241901865-241901988 | 2 |  |
| 4620 | 2 | 1 | 0 | '-' | 6 | 242605566-242605751 | 2 | ATG4B |
| 4632 | 2 | 0 | 1 | '+' | 6 | 821854-822198 | 20 | FAM110A |
| 4644 | 2 | 0 | 1 | '+' | 6 | 3693158-3693179 | 20 |  |
| 4655 | 2 | 1 | 0 | '-' | 6 | 13620031-13620048 | 20 | TASP1 |
| 4659 | 2 | 0 | 1 | '+' | 6 | 19955806-19955868 | 20 | RIN2 |
| 4666 | 2 | 0 | 1 | '+' | 6 | 23067691-23067771 | 20 | CD93 |
| 4678 | 2 | 0 | 1 | '+' | 6 | 31352399-31352874 | 20 | DNMT3B |
| 4690 | 2 | 1 | 0 | '-' | 6 | 35833182-35833240 | 20 | RPN2 |
| 4692 | 2 | 1 | 0 | '-' | 6 | 36152813-36152923 | 20 | BLCAP |
| 4693 | 2 | 1 | 0 | '-' | 6 | 36157405-36157760 | 20 | BLCAP |
| 4696 | 2 | 1 | 0 | '-' | 6 | 37054880-37054900 | 20 | LOC388796, SNORA71B |
| 4698 | 2 | 0 | 1 | '+' | 6 | 39320532-39320926 | 20 |  |
| 4705 | 2 | 0 | 1 | '+' | 6 | 44518802-44518824 | 20 | CTSA, NEURL2 |
| 4756 | 2 | 0 | 1 | '+' | 6 | 34185927-34186122 | 21 | C21orf62 |
| 4779 | 2 | 0 | 1 | '+' | 6 | 39870397-39870704 | 21 | ERG |
| 4780 | 2 | 0 | 1 | '+' | 6 | 39871287-39871301 | 21 | ERG |
| 4783 | 2 | 1 | 0 | '-' | 6 | 40145361-40145404 | 21 | NCRNA00114 |
| 4795 | 2 | 0 | 1 | '+' | 6 | 45232232-45232601 | 21 | LOC284837 |
| 4802 | 2 | 1 | 0 | '-' | 6 | 45713704-45713719 | 21 | AIRE |
| 4810 | 2 | 1 | 0 | '-' | 6 | 46890907-46890997 | 21 | COL18A1 |
| 4818 | 2 | 1 | 0 | '-' | 6 | 17680477-17680706 | 22 | CECR1 |
| 4832 | 2 | 0 | 1 | '+' | 6 | 23523942-23524136 | 22 | BCR |
| 4834 | 2 | 0 | 1 | '+' | 6 | 23922454-23922551 | 22 | IGLL1 |
| 4835 | 2 | 0 | 1 | '+' | 6 | 24105087-24105147 | 22 | C22orf15 |
| 4856 | 2 | 1 | 0 | '-' | 6 | 30877800-30877907 | 22 | SDC4P |
| 4857 | 2 | 0 | 1 | '+' | 6 | 31644160-31644190 | 22 | LIMK2 |
| 4867 | 2 | 0 | 1 | '+' | 6 | 37404577-37404888 | 22 | C22orf33 |
| 4918 | 2 | 0 | 1 | '+' | 6 | 47081634-47081751 | 22 | CERK |
| 4924 | 2 | 1 | 0 | '-' | 6 | 50356763-50357214 | 22 | PIM3 |
| 4933 | 2 | 0 | 1 | '+' | 6 | 50747014-50747040 | 22 | PLXNB2 |
|  |  |  |  |  |  |  |  |  |
